# Laterally substituted symmetric and nonsymmetric salicylideneimine-based bent-core mesogens

**DOI:** 10.3762/bjoc.8.15

**Published:** 2012-01-24

**Authors:** Sonja Findeisen-Tandel, Wolfgang Weissflog, Ute Baumeister, Gerhard Pelzl, H N Shreenivasa Murthy, Channabasaveshwar V Yelamaggad

**Affiliations:** 1 Martin-Luther-Universität Halle-Wittenberg, Institut für Chemie, Physikalische Chemie, von-Danckelmann-Platz 4, 06120 Halle, Germany; 2 Centre for Soft Matter Research, Jalahalli, Bangalore 560 013, India

**Keywords:** antiferroelectric smectic phases, bent-core mesogens, electro-optical behaviour, liquid crystals, salicylideneanilines

## Abstract

Bent-core mesogens have gained considerable importance due to their ability to form new mesophases with unusual properties. Relationships between the chemical structure of bent-core molecules and the type and physical properties of the formed mesophases are relatively unknown in detail and differ strongly from those known for calamitic liquid crystals. In this paper symmetric and nonsymmetric five-ring salicylideneaniline-based bent-core mesogens are presented, and the effect of lateral substituents attached at the outer phenyl rings (F, Cl, Br) or the central phenyl ring (CH_3_) on the liquid-crystalline behaviour and on the physical properties is studied. Corresponding benzylideneaniline-based compounds were additionally prepared in order to study the influence of the intramolecular hydrogen bond. The occurring mesophases were investigated by differential scanning calorimetry, polarising microscopy, X-ray diffraction and dielectric and electro-optical measurements. The paper reports on new findings with respect to the structure–property relationships of bent-core mesogens. On one hand, the disruptive effect of laterally substituted halogen atoms, F, Cl and Br, on the mesophase behaviour of three isomeric series was much lower than expected. On the other hand, an increase of the clearing temperature by 34 K was observed, caused by small lateral substituents. The electro-optical behaviour, especially the type of polar switching and corresponding molecular movements, is sensitive to variations in the molecular structure.

## Introduction

For a long time there was a general perception that molecules capable of exhibiting liquid crystal (LC) phases have to be rodlike, so-called calamitic LCs. As is well known, such compounds exhibit nematic and smectic phases, which are commonly referred to as “calamitic phases”. However, the inherent fallacy of this notion was pointed out in the middle of the seventies when columnar (Col) phases were discovered in disklike (discotic) mesogens; such mesogens also exhibit nematic behaviour like that of calamitics. In fact, a survey of the literature shows clearly that molecules with a bent (banana) shape prepared earlier than discotics by Vorlaender (in 1929) possess the ability to exhibit mesomorphism. Interest in such nonconventional mesogens increased remarkably in the mid-1990s following the report by Niori and co-workers [1] that the mesophases (banana phases) formed by 1,3-disubstituted benzenes hold unusual properties. As a result, a large number of banana-shaped compounds have been synthesized with various combinations of structural fragments, and thus, they represent a new subfield of liquid crystals. Generally speaking, these mesogens prefer to pack in layers so as to yield smectic phases. Due to their bent shape there is a lateral correlation of molecular dipoles yielding a polar order within the layers, which can be switched on application of an electric field. The molecular arrangement of bent-core molecules, with respect to the tilt and the polarity in adjacent layers of a SmCP phase, is shown in Supporting Information File 1, Figure S1. An explanation of the suffixes **P**, **A**, **F**, **s**, and **a**, used for the characterization of banana phases, is also given there. In the SmCP phase the molecular long axis is tilted with respect to the layer normal, which corresponds to a *C*_2_ symmetry of the smectic layers. The combination of director tilt and polar order leads to a chirality of the smectic layers, although the constituent molecules are achiral [2]. In this context it should be noted that there is a tilted smectic “banana phase” designated as SmCG, which exhibits chiral smectic layers with *C*_1_ symmetry [3–4].

In order to avoid a macroscopic polar order, in most cases the polar axes in adjacent layers are antiparallel such that, in general, antiferroelectric SmCP_A_ phases occur, which can be switched into the corresponding ferroelectric state. This switching, and also the switching between opposite ferroelectric states, usually takes place by the collective rotation of the molecules around the tilt cone. It is seen from Supporting Information File 1, Figure S1 that during this switching, the chirality of the layers does not change. However, there are SmCP phases where the switching is based on a collective rotation of the molecules around their long molecular axes. In this case the chirality of the smectic layers is changed by the switching process, which is a prominent feature observed in the present investigation.

The formation of simple layer structures is disrupted if the amount of space required by the rigid cores and the flexible terminal chains is too different. Together with certain polar forces, such connection can result in undulating tilted smectic phases designated as USmCP, or columnar phases of different structure. The columns are represented by polar-layer fragments. Two examples are given: In the B_1_ phase, the columns form a 2D rectangular cell. However, the structural feature of B_7_ phases is a splay of polarization, which gives rise to an undulation of smectic layers or two-dimensionally ordered layer fragments [5].

Due to such unusual behaviour banana-shaped liquid crystals have emerged as a special topic in the field of liquid crystals. It should be pointed out here, that polar banana phases have only been formed by bent molecules up until now. Although a bent conformation is also exhibited by dimesogens due to the presence of odd-parity central spacer, they do not spontaneously form polar banana phases [6–7].

In general, banana-shaped molecules consist of five or more aromatic rings, in which two wings (rodlike arms) are attached to the 1,3-positions of a central phenyl ring. The vast majority of the banana-shaped mesogens contain ester and/or azomethine linking groups. It is remarkable that derivatives containing an azomethine group (Schiff bases) often show interesting polymorphism. Relationships between molecular structure and the formation of banana phases have been summarized, together with the structure and the behaviour of these phases, in several reviews, see e.g., [8–11].

It is well-known that a hydroxy group in ortho-position to the CH=N (azomethine) unit not only enhances the photochemical and hydrolytic stability due to intramolecular H-bonding, but also increases the clearing temperature of liquid crystals. Therefore, the introduction of salicylideneimine fragment(s) in the LC molecular architecture has attracted significant attention. Several examples demonstrate this, as many salicylideneanilines exhibiting mesophases are listed, see e.g., [12–13]. These salicylideneanilines, but also salicylideneimines, can form metallomesogens by complexation with d- and f-block elements, [14–17]. Tris(salicylideneanilines) having a discotic shape exhibit a room-temperature columnar mesophase [18–19]. Dendromesogens containing up to 64 peripheral mesogenic salicylideneimine fragments have also been reported [20]. Antiferroelectric behaviour has been claimed by Yablonski et al. to be exhibited by achiral liquid-crystalline monomer–polymer mixtures containing salicylideneaniline moieties [21–22].

**Scheme 1:**
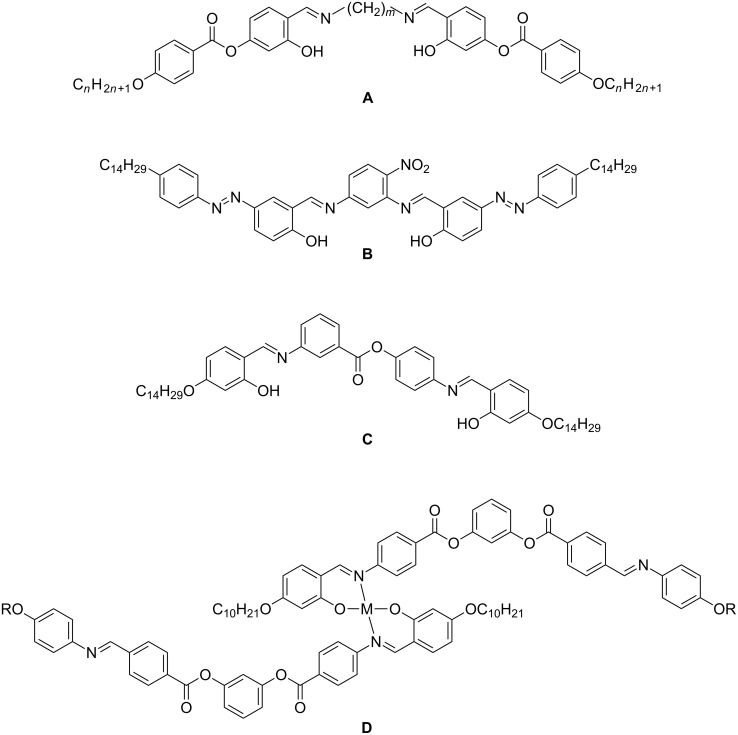
Examples for bent mesogens containing salicylideneimine moieties.

Interesting examples of bent molecules are given by the following: Two salicylideneimine moieties can be connected to each other to form twin molecules (Scheme 1A). In the case of an odd-numbered spacer these dimers exhibit a bent shape and form nematic, columnar nematic, smectic and columnar phases. Columnar phases were not found for the dimers having even-numbered spacers, because these molecules are more linear [23–27]. Interestingly, for a nonsymmetric chiral dimer the reentrant-phase behaviour SmA–SmA_b_–SmA was reported [28].

Rao et al. postulated a new chiral smectic phase formed by W-shaped achiral molecules that contain two salicylideneaniline units (Scheme 1B) [29–30]. Recently, new achiral four-ring molecules were reported by Deb et al., in which two substituted salicylideneaniline moieties are linked by an ester group (Scheme 1C). These compounds, which show a strong photoluminescence, exhibit typical banana phases B_1_ and B_7_ [31]. Yelamaggad et al. reported V-shaped five-ring mesogens: Two salicylideneaniline moieties are linked by ester groups to the 1,2-positions of a phenyl ring. Due to the low bending angle of about 70°, the molecules form a partially intercalated SmA phase instead of banana phases [32].

Banana phases can be expected when the bending angle is opened to about 120°. Already several bent-core mesogens with salicylideneaniline moieties have been reported. The molecules can have a symmetric or nonsymmetric structure. If the aromatic core consists of five phenyl rings containing one and two salicylideneaniline moieties we speak about mono- or disalicylideneaniline compounds.

Monosalicylideneaniline compounds with different chain lengths in the terminal positions have been reported. Additionally, the effect of a nitro group neighbouring the hydroxy group has been studied. A mixture of one of these derivatives with a chiral twin compound was investigated by dielectric methods [33–34]. Yelamaggad et al. [35] reported nonsymmetric molecules having two fluorine atoms in the 2,3-position of one outer phenyl ring. SmCP phases with an unusual electro-optical switching behaviour were observed. The SmCP phase of bidendate bent-core ligands disappears in the corresponding Cu(II) and Pd(II) metal complexes (Scheme 1D) [36].

In 2001, homologues of a series of symmetric compounds bearing both azomethine groups between the outer and the next neighbouring inner phenyl rings were reported for the first time by Yelamaggad et al. [37–39] who also investigated the phase behaviour under pressure and the effect of light on the polarization of a mixture of these salicylideneanilines with a photoactive azo compound. The observation of a B_7_ mesophase on the hexadecyloxy compound is surprising, because the central phenyl ring was not substituted by a nitro or cyano group as is the case for most of the other banana-shaped liquid crystals that form this phase type [40]. The nonyloxy homologue was studied in detail by Walba et al. For this compound, also abbreviated as NORABOW, the existence of the very rare SmCG mesophase was claimed [41]. Achten et al. compared the phase behaviour of the complete homologous series with that of the corresponding 1,5-pentylene connected dimers [27]. In 2007, we reported systematic investigations on the influence on the mesophase behaviour of lateral groups attached to the central phenyl ring of several homologous series [42].

Isomeric compounds, symmetric in the aromatic core, result when both azomethine groups are linked to the central phenyl ring. The influence on the mesomorphism of a fluoro substituent at the central phenyl ring was studied by Rao et al. [43]. Three years ago, we reported the synthesis and a remarkable electro-optical behaviour of three bent-core compounds derived from 2-methyl-1,3-phenylenediamine. The unusual current response showed five peaks per half period. Such multistage switching was measured for the first time on banana-shaped liquid crystals and has been proved by different electro-optical methods and optically observed by a high-speed camera [44–45].

It is not surprising that bent-core compounds having six phenyl rings can also form banana phases. Recently, the central phenyl ring of a salicylideneaniline based five-ring bent-core compound was replaced by a biphenyl moiety, leading to SmCP phases [46].

In continuation of our work on salicylideneaniline-based banana-shaped mesogens, we herein report a study of the effect of lateral substituents on the mesophase behaviour of symmetric and nonsymmetric five-ring bent-core mesogens. Up to now there have only been systematic investigations on the effect of atoms and groups introduced at the central phenyl ring. In our work, we now attach halogen atoms at the outer phenyl rings. When nonsymmetric compounds are laterally substituted with one halogen atom, this can be introduced in either one of both of the molecular legs, giving isomeric compounds, which will allow a detailed comparison. A further goal of the present work is the synthesis of bent-core salicylideneaniline compounds bearing a lateral methyl group at the central phenyl ring, in search of further materials that may also show the unusual multistage switching as mentioned above. To assess the effect of the intramolecular hydrogen bond on the mesophase behaviour, corresponding compounds without hydroxy groups were additionally prepared. In order to focus the study on the effect of lateral substituents, the hydrocarbon chains at both terminal phenyl rings were fixed with dodecyloxy groups.

## Results and Discussion

### Monosalicylideneaniline compounds **OH 1** bearing the azomethine group between an outer phenyl ring and its neighbour

#### Synthesis of the compounds **OH 1**

**Scheme 2:**
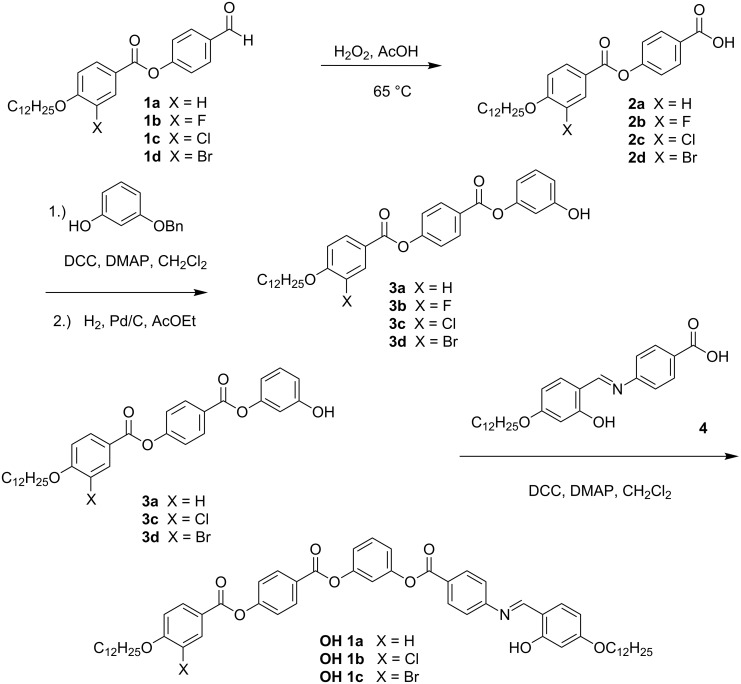
Synthetic pathway to prepare compounds **OH 1**.

The synthesis of the compounds **OH 1**, which contain the azomethine connection group between an outer phenyl ring and the adjacent ring of the five-ring bent-core mesogens, is sketched in Scheme 2. The 4-(4-dodecyloxy-3-halogenobenzoyloxy)benzoic acids **2** were prepared by acylation of 4-hydroxybenzaldehyde with the corresponding 4-dodecyloxy-3-halogenobenzoic acids following oxidation of the formyl compounds **1**. The phenolic intermediates **3** were obtained by reaction of 3-benzyloxyphenol with the substituted benzoic acids **2** and hydrogenolytic deprotection of the hydroxy group according to [47]. The final products **OH 1a–c** were obtained by esterification of the phenols **3** with 4-(4-dodecyloxy-2-hydroxybenzylideneamino)benzoic acid (**4**), which exhibits a liquid-crystalline phase itself (Cr 204 N 269 I) [42]. It should be mentioned that Yelamaggad et al. [36] preferred a condensation step between the 4-*n*-alkyloxy-2-hydroxybenzaldehyde and the related four-ring amino compound for the synthesis of short-chain homologues of the laterally unsubstituted compound **OH 1a** to study the complexation with Cu(II) and Pd(II).

#### Mesophase behaviour of the compounds **OH 1**

The transition temperatures and associated enthalpies of the compounds **OH 1** are summarized in Table 1. The introduction of the hydroxy group causes an increase of the clearing temperature by approximately 30 K (compare, e.g., compounds **H 1a** and **OH 1a**). This stabilization effect is already well-known from calamitic Schiff bases and is caused by the intramolecular hydrogen bond, which could also reduce the flexibility of this molecular leg. Halogen substituents in position X have only a modest effect on the mesophase stability. A slight decrease in the clearing temperature with increasing size of the substituent is observed. We had expected a stronger effect caused by a lateral chlorine or bromine atom.

**Table 1:** Mesophase behaviour, transition temperatures (°C) and enthalpies [kJ/mol], and *P_S_* values (nC/cm^2^) of compounds **OH 1** and **H 1a**.

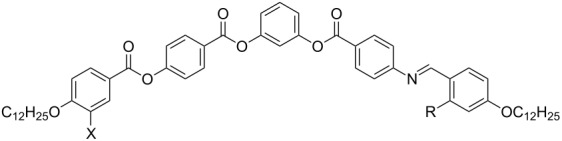								

No.	R	X	Cr		SmCP_A_		I	*P*_S_

**H 1a**	H	H	•	92 [62.3]	•	111 [19.8]	•	750
**OH 1a**	OH	H	•	80 [44.7]	•	142 [22.4]	•	900
**OH 1b**	OH	Cl	•	82 [22.5]	•	140 [19.2]	•	390
**OH 1c**	OH	Br	•	85 [22.1]	•	134 [16.9]	•	250

**Figure 1:**
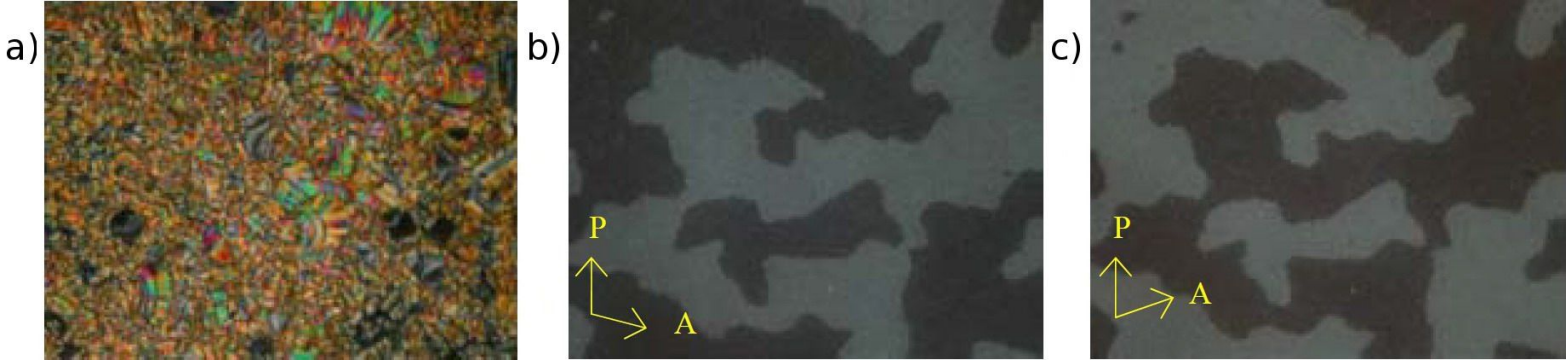
Polarising optical microscopy images. a) Compound **OH 1a**: Grainy texture with smectic Schlieren areas at 130 °C; b), c) compound **OH 1c**: Chiral domains of opposite handedness at 125 °C; polarisers decrossed from 90° by about +/-5°.

By cooling the isotropic liquid of **H 1a** and **OH 1a** a birefringent grainy texture together with areas showing a Schlieren texture were observed by polarising optical microscopy (Figure 1a), which is a hint for a layer structure. In contrast, the mesophases of the halogen-substituted compounds **OH 1b** and **OH 1c** appeared optically isotropic with crossed polarisers. By rotation of one of the polarisers clockwise from the crossed (90°) position, bright and dark domains became visible (Figure 1b). Rotating the polariser anticlockwise by the same angle made the dark domains bright, and vice versa (Figure 1c). Obviously domains of opposite handedness occur. One explanation is the bookshelf geometry of smectic layers in which an anticlinic arrangement with a tilt angle of 45° leads to an orthoconic structure. This state is nonbirefringent [48–49].

**Figure 2:**
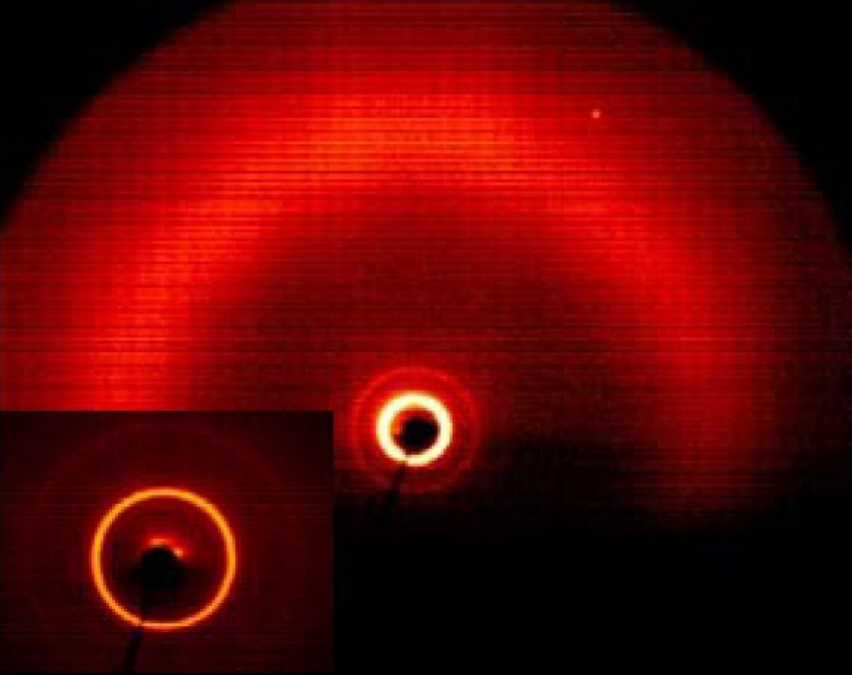
X-ray diffraction pattern of a powderlike sample of **OH 1b** at 131 °C (inset: Small angle region; lower part of the patterns shadowed by the heating stage).

X-ray diffraction experiments on powderlike samples of **OH 1a** and **OH 1b** showed first and second order layer reflections and a diffuse outer scattering, revealing a simple layer structure having a liquidlike state within the layers (Figure 2). The layer thicknesses amount to *d* = 4.0 nm (**OH 1a**) and *d* = 4.2 (**OH 1b**). Based on a calculated molecular length of *L* ~ 5.4 nm, the molecular long axes are tilted with respect to the layer normal by about 42°, excluding an intercalation of the terminal chains.

**Figure 3:**
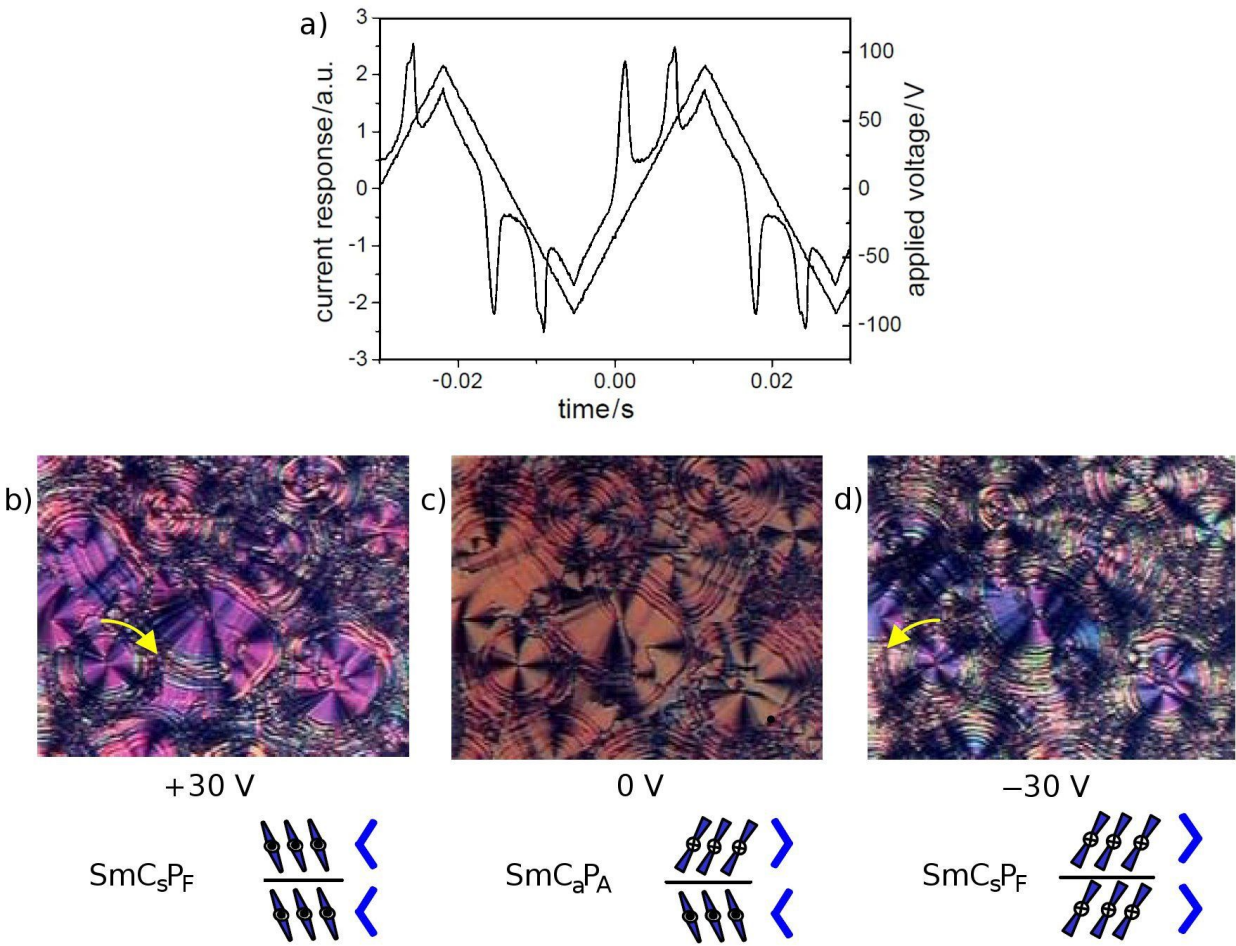
Electro-optical behaviour of compound **OH 1a**: a) current response curve (*U* = 182 V_pp_, *f* = 30 Hz, *T* = 112 °C, *d*_cell_ = 6 μm, *P*_S_ = 900 nC/cm^2^); b–d) tristable switching of circular domains by a D.C. voltage. *T* = 112 °C, *d*_cell_ = 6 μm; c) 0 V, extinction crosses parallel to the crossed polariser; b) +30 V, clockwise rotation of the extinction crosses (see yellow arrow); d) −30 V, anticlockwise rotation of extinction crosses (see yellow arrow).

EHC cells consisting of two thin glass plates with a conductive surface (ITO) were used for electro-optical investigations. After the cell is filled with the material under study, the application of an electric field gives information on the dielectric properties and polar switching processes. The current response curve of a low-frequency triangular wave voltage shown in Figure 3a gives evidence that the tilted smectic phase exhibits an antiferroelectric switching behaviour. Cooling the isotropic liquid in the presence of a D.C. field leads to the formation of circular domains, in which the layers are arranged cylindrically. In the ground state the extinction brushes are parallel to polariser and analyser, see Figure 3c. From this observation it follows that the antiferroelectric SmCP phase exhibits an anticlinic arrangement (SmC_a_P_A_). By application of an electric field the extinction brushes rotate clockwise or anticlockwise depending on the polarity of the field, see Figure 3b and Figure 3d. Under the field the molecules are arranged with the polar axes in the field direction such that a synclinic and ferroelectric state results. When the field is switched off, the molecules rotate back to the antiferroelectric ground state. The arrangement of the molecular long axes in two adjacent layers is sketched below the textures in Figure 3. This behaviour was found for all compounds **OH 1a–c**. The values of the spontaneous polarization are given in Table 1.

**Figure 4:**
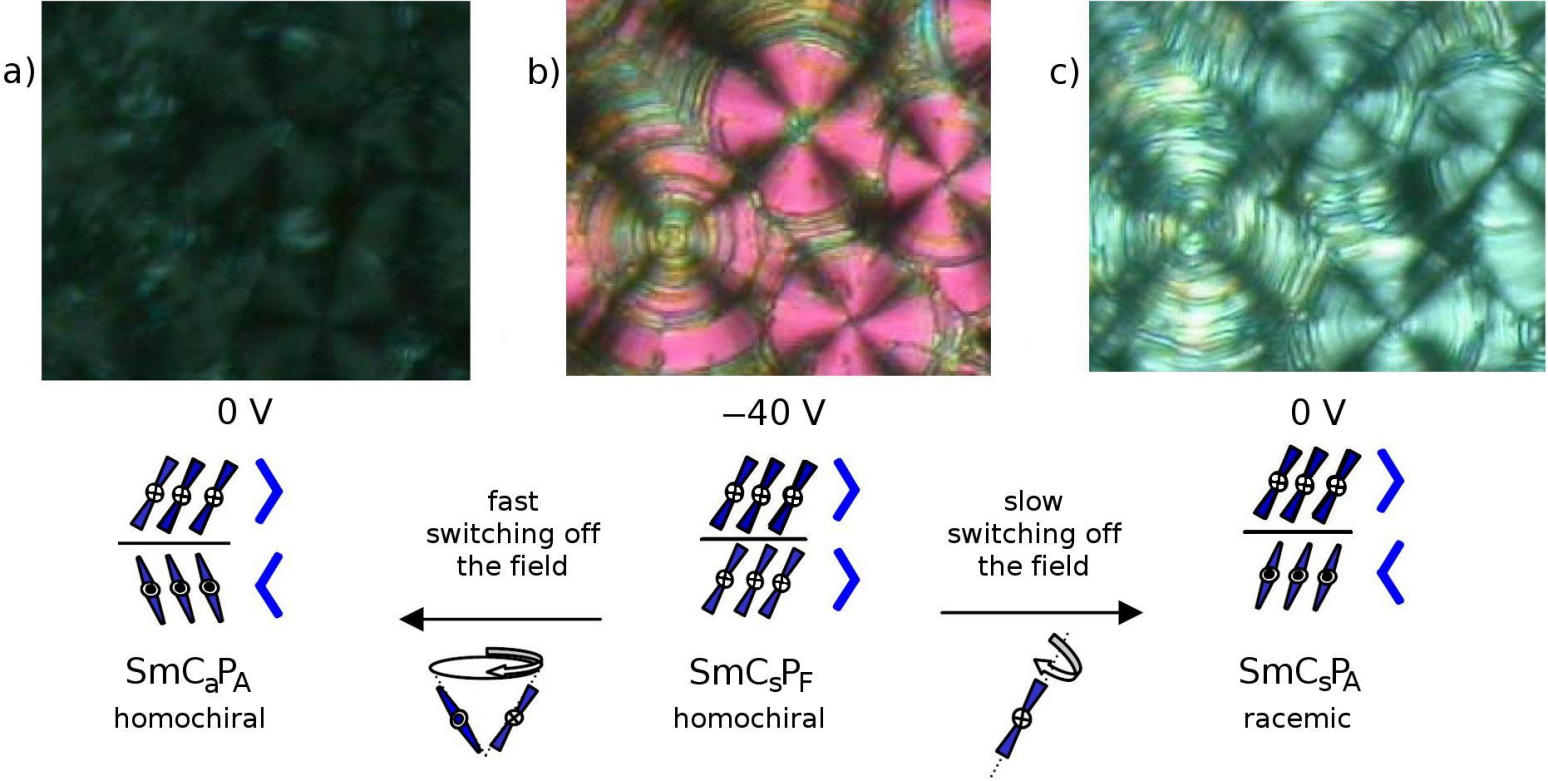
Switching behaviour of compound **H 1a**, which depends on how quickly the electric field is switched off.

The switching behaviour of the compound **H 1a** without the hydroxy group is more complicated (Figure 4). When an electric field is applied, circular domains are formed (Figure 4b). In the circular domains the smectic layers are cylindrically arranged around the centre of the domains. This arrangement is indicated by the occurrence of extinction crosses. For tilted smectic phases the angle between the extinction crosses and the crossed polarisers corresponds to the tilt angle of the smectic phase. As shown in Figure 4b, circular domains appear under application of a sufficiently high electric field. After removal of the electric field, the relaxation of the field-induced circular domains strongly depends on the experimental conditions. When the electric field is removed quickly, the extinction crosses rotate back to the crossed polariser position. The texture becomes dark and the crosses are difficult to recognize, as shown in Figure 4a. The reason for this is that for smectic layers perpendicular to the substrate an antiferroelectric arrangement with a tilt angle of about 45° leads to an orthoconic structure. This state is nonbirefringent in the direction perpendicular to the substrate and therefore dark between crossed polarisers [49]. Upon reapplication of the electric field, the crosses rotate back to the 45° positions (Figure 4a and Figure 4b). When the field is removed very slowly (about 2 V/s) the circular domains do not change, and only a slight decrease in the birefringence is seen (Figure 4c). When the electric field is applied again, the extinction crosses do not rotate. Different movements of the molecular long axis are responsible for such distinct behaviour: In most cases the molecular long axes rotate on a cone, as sketched in Figure 4 below the texture. In the other case, the crosses do not change when the molecules of each second layer rotate by 180° about their long axes (sketch on the right below the textures in Figure 4).

In this case, of rotation about the long axes, the chirality changes from the chiral SmC_s_P_F_ phase to the racemic SmC_s_P_A_ phase. This means that the chirality is changed in every second layer. Such flipping of chirality has been reported by Schröder et al. in 2004 [48] for the SmCP phases and by Szydlowska et al. in 2003 [50] for the B_1_ phase. Both processes of molecular movement require different energies, which correspond to different coefficients of viscosity for both molecular movements [51].

### Monosalicylideneaniline compounds **OH 2** bearing the azomethine group between the central phenyl ring and one neighbouring ring

#### Synthesis of the compounds **OH 2**

**Scheme 3:**
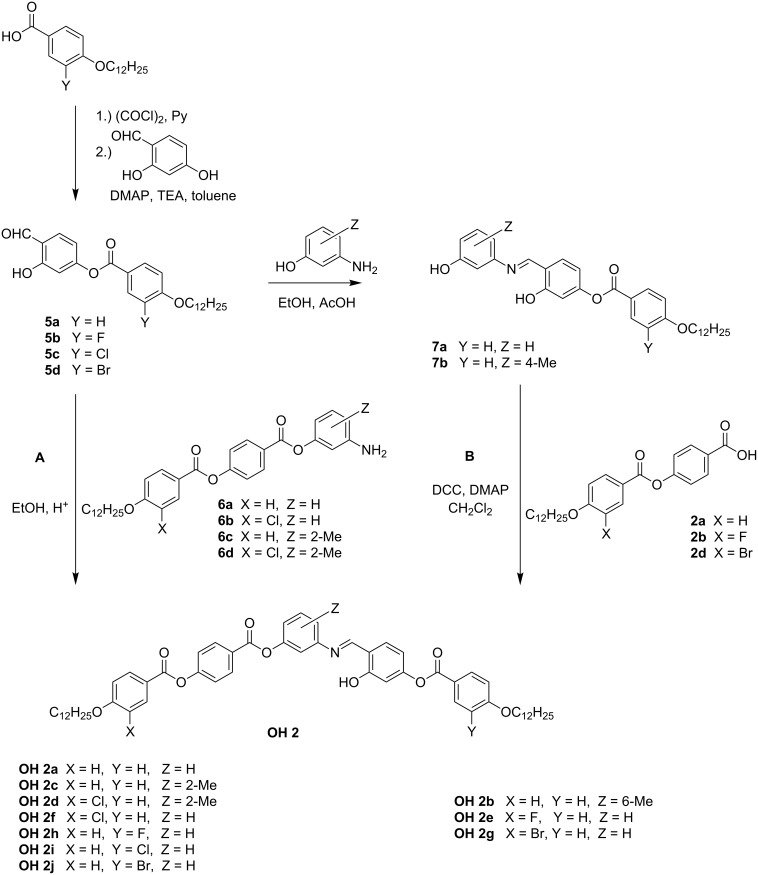
Reaction pathways to prepare the monosalicylideneaniline compounds **OH 2a–j**.

The compounds **OH 2** have one azomethine linking group between the central phenyl ring and the adjacent ring bearing a hydroxy group. Two reaction pathways to prepare these compounds are sketched in Scheme 3. The basic intermediates were the 4-formyl-3-hydroxyphenyl 4-*n*-dodecyloxy-3-substituted-benzoates **5a–d**, which were synthesized by acylation of 2,4-dihydroxybenzaldehyde with the corresponding 4-*n*-dodecyloxy-3-substituted-benzoyl chlorides prepared by use of oxalyl chloride. Following pathway **A** the condensation of the salicylaldehydes **5a–d** with 3-aminophenyl 4-(4-*n*-dodecyloxy-3-substituted-benzoyloxy)benzoates **6** yielded the final compounds according to [33].

Following pathway **B**, the salicylidene intermediates **5a–d** were reacted with 3-aminophenols to give the phenols **7a**,**b**. Subsequently, their esterification with 4-(4-*n*-dodecyloxy-3-substituted-benzoyloxy)benzoic acids **2** was the final step needed to afford the compounds **OH 2b**, **2e**, **2g**. The selection of route **A** or **B** was determined by the better purity of the final products, but short reaction paths for the expensive intermediates, e.g., the fluoro-containing compounds, were also an important consideration.

#### Mesophase behaviour of the compounds **OH 2**

Transition temperatures and corresponding enthalpies from calorimetric measurements are summarized in Table 2. For comparison, the compound **H 2a** without the hydroxy group is added.

**Table 2:** Mesophase behaviour, transition temperatures (°C), transition enthalpies [kJ/mol] and *P*_S_ [nC/cm^2^] values of the compounds **OH 2** and **H 2a**.

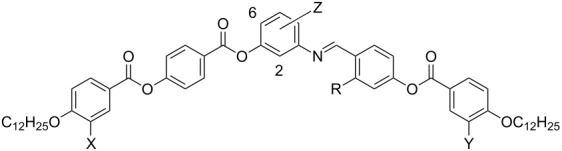										

No.	R	X	Z	Y	Cr		SmCP_A_		I	*P*_S_

**H 2a**	H	H	H	H	•	111 [65.5]	•	(107) [16.9]	•	920
**OH 2a** [33]	OH	H	H	H	•	105 [63.1]	•	136 [22.4]	•	670
**OH 2b**	OH	H	6-CH_3_	H	•	83 [32.0]	•	105 [16.3]	•	630
**OH 2c**	OH	H	2-CH_3_	H	•	125 [27.4]	•	132 [19.5]	•	—^a^
**OH 2d**	OH	Cl	2-CH_3_	H	•	125 [11.7]	•	133 [18.9]	•	420
**OH 2e**	OH	F	H	H	•	107 [47.4]	•	142 [21.2]	•	730
**OH 2f**	OH	Cl	H	H	•	68 [22.1]	•	138 [20.7]	•	730
**OH 2g**	OH	Br	H	H	•	66 [24.8]	•	132 [18.9]	•	470
**OH 2h**	OH	H	H	F	•	107 [30.4]	•	143 [21.8]	•	660
**OH 2i**	OH	H	H	Cl	•	79 [56.9]	•	135 [21.7]	•	640
**OH 2j**	OH	H	H	Br	•	56 [24.3]	•	133 [20.1]	•	590

^a^Despite many purification procedures, *P*_S_ could not be reproducibly measured due to the high conductivity of the compound.

All compounds **OH 2** form a SmCP phase. The clearing temperature of the benzylideneaniline **H 2a** is increased by 29 K by introduction of the hydroxy group in the ortho position of the azomethine linking group, to give **OH 2a**. The effect of a methyl group on the mesophase stability depends on the position on the central phenyl ring. The lowering of the smectic–isotropic phase-transition temperature by introduction of the lateral methyl group in the 6-position, **OH 2b**, amounts to 31 K. The influence of a methyl group in the obtuse angle of **OH 2c**, that is in position 2, can be virtually ignored, and surprisingly also the combination with a chlorine atom laterally attached to one of the outer rings in **OH 2d** has little effect. Comparing the different sizes and polarities of the halogen atoms in the compounds **OH 2e–j**, a fluorine atom increases the clearing temperatures by about 6 K (**2e**, **2h** in comparison to the laterally nonsubstituted compound **OH 2a**). The chlorine- and bromine-substituted compounds have transition temperatures that are only a little lower. Although the bromine atom is a relatively large substituent, the clearing temperatures are, surprisingly, depressed by only few degrees. Furthermore, the disrupting effect is comparable for the both series **OH 2e–g** and **OH 2h–j**, which are isomeric to each other. Here, the halogen atoms are attached to the “left” or “right” arms, which are chemically different. It should also be mentioned that by introduction of one halogen atom in these nonsymmetric molecules the melting temperatures can strongly decrease. Therefore, relatively broad enantiotropic mesophase ranges result, for example, 77 K for compound **OH 2j**.

**Figure 5:**
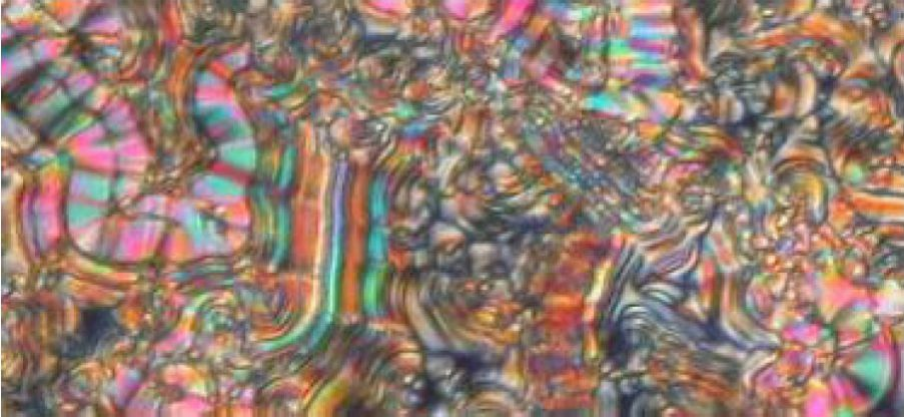
Compound **OH 2a**: Texture of the SmCP phase at 126 °C.

Observation by polarising optical microscopy shows textures typical for SmCP phases, for example fan-shaped, ribbonlike and Schlieren textures (Figure 5).

**Figure 6:**
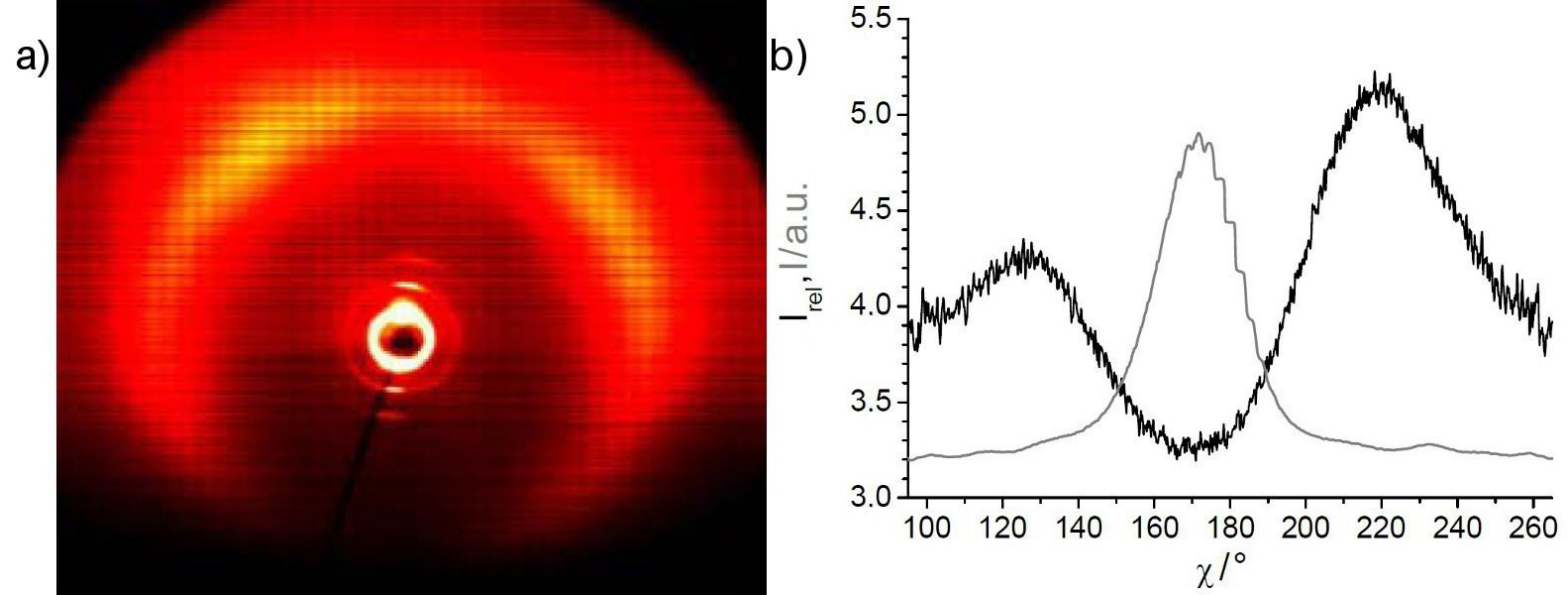
a) 2D X-ray pattern of a surface-aligned sample of compound **OH 2a** at 128 °C (lower part of the pattern shadowed by the heating stage); b) χ scan for the outer diffuse scattering with maxima at χ ≈ 125 and 220° [I_rel_ = I(128 °C)/I(140 °C, isotropic liquid], and the position of the layer reflections for comparison (gray line).

X-ray diffraction studies on **OH 2a** give evidence for a layer structure with a liquidlike order within the layers. The layer distance, with *d* = 3.8 nm, is significantly smaller than the calculated molecular length (*L* ~ 5.4 nm). According to cos θ = *d*/*L*, a tilt angle of the molecular long axis with respect to the layer normal of 45° results at a temperature of 130 °C. A similar tilt angle of 42° was determined from the mutual positions of the maxima of the outer diffuse scattering and of the small-angle reflections in the 2D diffraction patterns (Figure 6). The significant difference in the intensities of the two maxima of the outer diffuse scattering is a hint to a synclinic arrangement of the molecules in adjacent layers.

**Figure 7:**
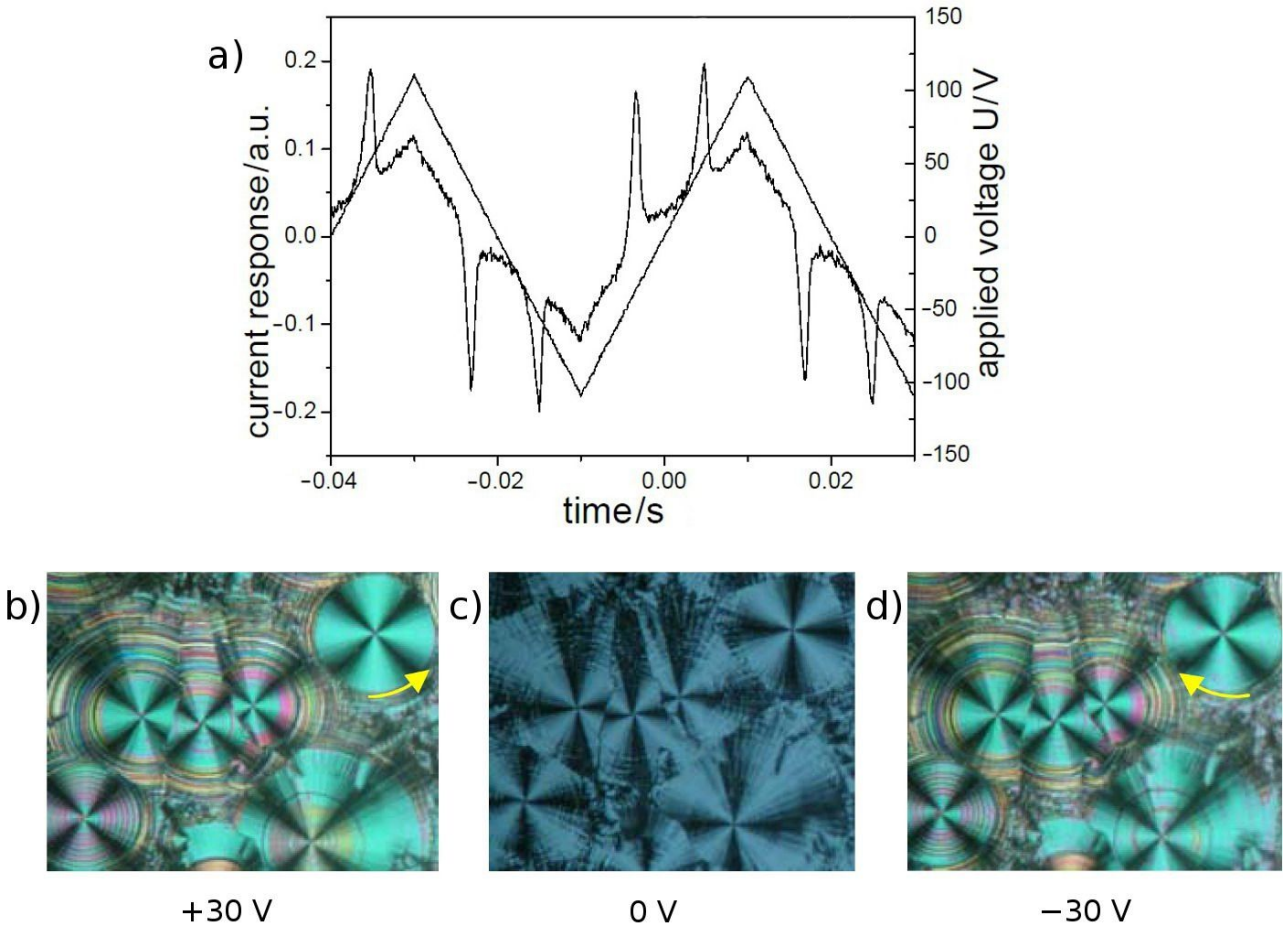
Electro-optical switching behaviour of compound **OH 2a**: a) current response (*U* = 230 V_pp_, *f* = 25 Hz, *T* = 106 °C, *d*_cell_ = 6 μm; *P*_S_ = 660 nC/cm^2^); b–d) tristable switching on homogeneous chiral domains; starting from texture c) where the extinction crosses are parallel to the crossed polariser, the extinction crosses rotate anticlockwise in b) and clockwise in d) in dependence on the polarity of the D.C. electric field.

Electro-optical measurements on **OH 2a** by using a triangular wave voltage show a current response consisting of two repolarization peaks, which proves an antiferroelectric ground state. Figure 7 shows the switching behaviour and corresponding textures. The extinction crosses rotate to the right or left in response to the polarity of the electric field. This behaviour is characteristic of a tristable switching process between the tilted anticlinic antiferroelectric (SmC_a_P_A_) and the synclinic ferroelectric (SmC_s_P_F_) states. The value of the spontaneous polarization was calculated from the area under the repolarization peaks to be 660 nC/cm^2^. The switching behaviour of **OH 2a** is representative for all compounds **OH 2a–j**. The *P*_S_ values are given in Table 2.

### Disalicylideneaniline compounds **OH 3** and **OH 4** bearing the azomethine groups between the outer and neighbouring phenyl rings

#### Synthesis of the compounds **OH 3** and **OH 4**

**Scheme 4:**
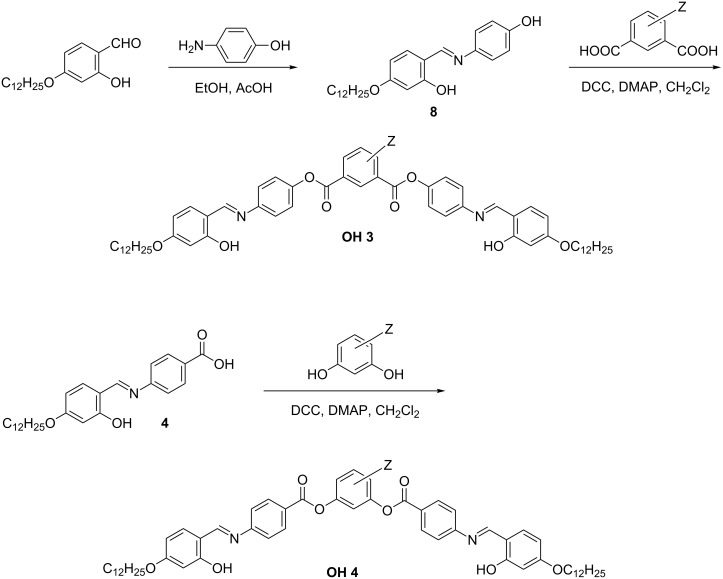
Synthetic steps followed to prepare the compounds **OH 3** and **OH 4**.

Compounds **OH 3** and **OH 4** consist of two 4-*n*-dodecyloxysalicylideneaniline moieties, which are connected to the central phenyl ring by ester groups. These linking groups have different directions in the compounds **OH 3** and **OH 4**. Esterification of the carboxylic acid **4** or of the phenolic intermediate **8** with the corresponding resorcinols and isophthalic acids, yielded the compounds **OH 3** and **OH 4**, respectively, as sketched in Scheme 4.

#### Mesophase behaviour of the compounds **OH 3**

Table 3 shows the transition temperatures of the isophthalic acid derivatives **OH 3a–c** together with those of the corresponding esters **H 3a–c**, which do not exhibit an ortho-hydroxy group, for comparison.

**Table 3:** Mesophase behaviour, transition temperatures (°C) and enthalpies [kJ/mol] of compounds **OH 3** (on the right) and compounds **H 3** (on the left).

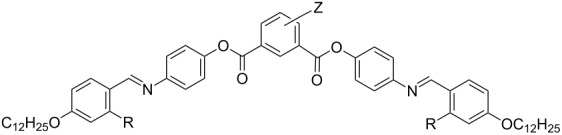				

Mesophase behaviour	R = H	Z	R = OH	Mesophase behaviour

Cr 190 [98.0] I	**H 3a**	H	**OH 3a**	Cr 179 [63.5] SmCP_A_ 223 [22.5] I
Cr 163 [84.2] (SmCP_A_ 155 [28.2]) I	**H 3b**	4-Br	**OH 3b**	Cr 136 [40.8] SmCP_A_ 189 [21.6] I
Cr 193 [57.5] I	**H 3c**	5-NO_2_	**OH 3c**	Cr 224 [61.9] I

Liquid-crystalline phases were not observed for the benzylidene derivative **H 3a**. The introduction of the hydroxy groups enables the salicylideneaniline compound **OH 3a** to form a mesophase. The clearing temperature of the bromine-substituted compound **OH 3b** increases due to the presence of hydroxy groups, in comparison to **H 3b**, by 34 K. Liquid-crystalline behaviour was not observed for either of the nitro derivatives **H 3c** or **OH 3c**, probably because the 5-position of the nitro group, which is at the top of the bent molecules, is sterically unfavourable in most cases. Generally, melting temperatures and phase-transition temperatures of isophthalic acid derivatives are much higher in comparison to those of the isomeric resorcinol derivatives (see compounds **OH 4**), as also proved for two other series of isomeric five-ring mesogens containing only ester linking groups [47].

**Figure 8:**
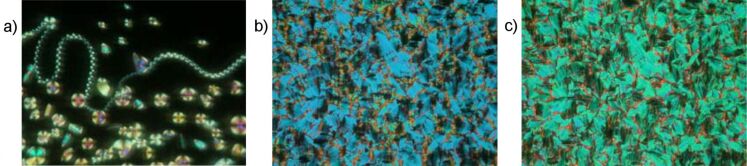
Optical photomicrographs of compound **OH 3a**: a) On cooling of the isotropic liquid; c) *U* = 0 V; b) *U*_D.C._ = ±67 V, T = 172 °C, *d*_cell_ = 6 μm.

Helical filaments and circular domains grow upon cooling of the isotropic liquid of compound **OH 3a** (Figure 8a), which coalesces into a nonspecific texture. Such spiral germs could be a hint for undulated layer structures or a B_7_ phase. X-ray patterns on nonoriented samples display two orders of layer reflections (*d* = 4.7 nm), however do not provide any evidence for a modulation or undulation of the layers.

**Figure 9:**
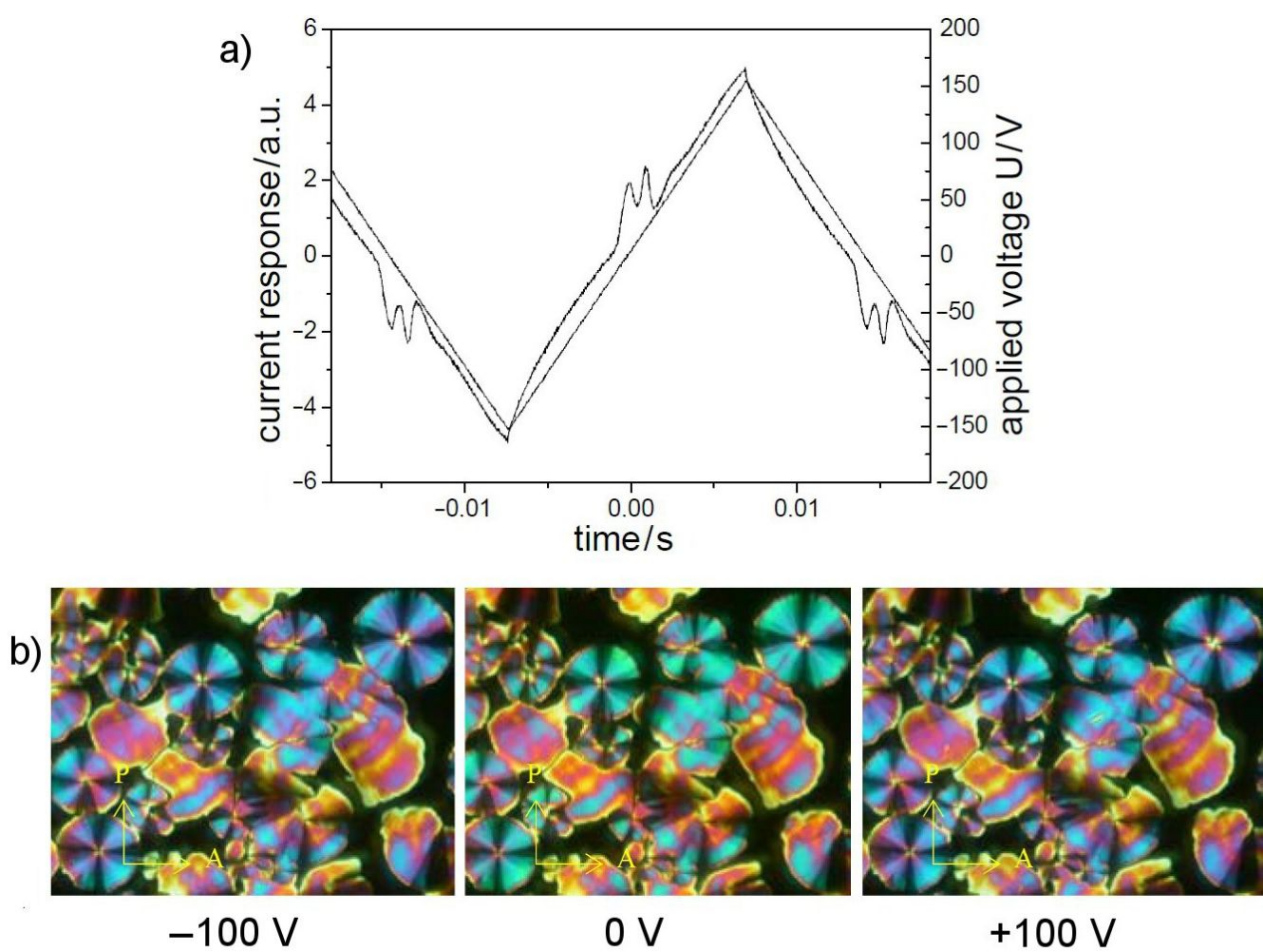
Electro-optical behaviour of compound **OH 3a**: a) Current response curve (*U* = 308 V_pp_, *f* = 35 Hz, *T* = 172 °C, *d*_cell_ = 6 μm, *P*_S_ = 370 nC/cm^2^); b) texture of the SmCP_A_ phase depending on the polarity of the applied field.

Upon application of an electric field, the birefringence slightly changes, as shown in Figure 8b and Figure 8c for a D.C. field. In Figure 9b, the parallel arrangement of extinction crosses with respect to the position of the crossed polarisers indicates an anticlinic arrangement of the molecules in adjacent layers. With high fields, two repolarization peaks per half period were separated, which is typical for an antiferroelectric switching of a SmC_a_P_A_ phase (Figure 9a). The extinction crosses do not rotate and the electro-optical behaviour is independent of the polarity of the electric field. This behaviour suggests that the position of the optical axis and hence the direction of the molecular long axes does not change. Therefore, the switching process can be explained by a collective rotation of the molecules about their long axis.

**Figure 10:**
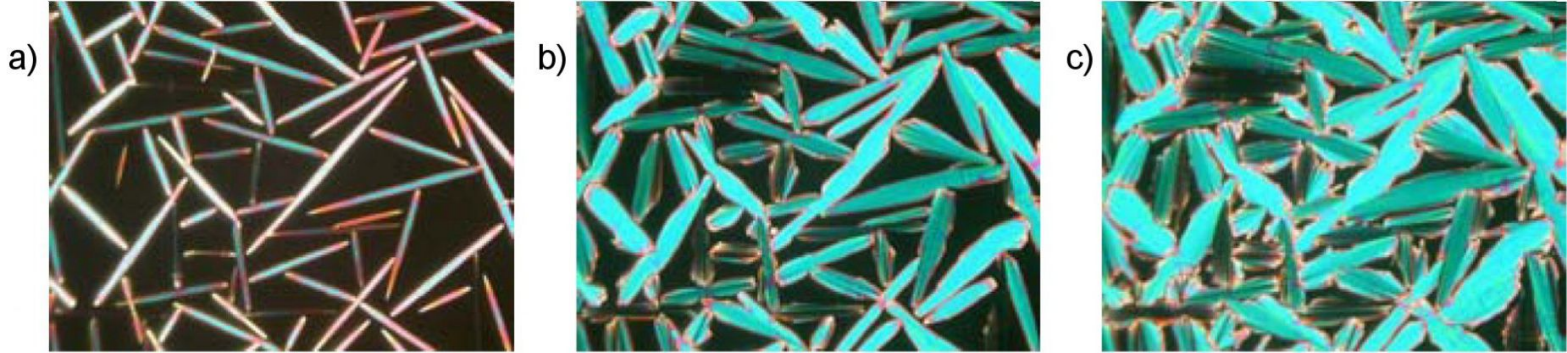
Growth of a fan-shaped texture from lancetlike filaments upon cooling of the isotropic liquid of compound **OH 3b**.

Substitution of the central phenyl ring with a bromine atom (Z = 4-Br) results in a broader mesophase range at lower temperatures for **OH 3b**. Upon cooling of the isotropic liquid, lancetlike filaments grow into a fan-shaped texture exhibiting some cross-stripes (Figure 10).

**Figure 11:**
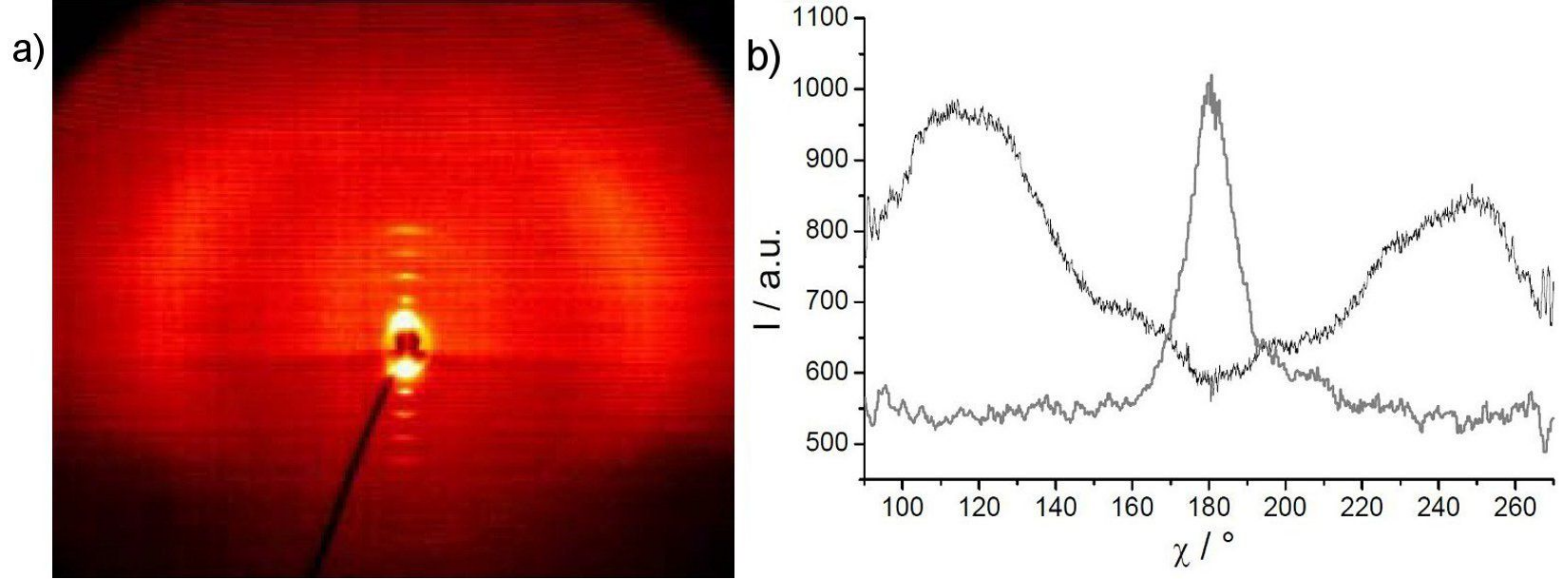
a) 2D X-ray pattern of a surface-aligned sample of compound **OH 3b** at 160 °C **(**lower part of the pattern shadowed by the heating stage); b) χ-scan for the diffuse scattering (black line), compared to the position of the layer reflections on the meridian (grey line).

X-ray patterns from partially aligned samples of the 4-bromo-isophthalic acid derivative **OH 3b** display up to five orders of the layer reflections on the meridian of the 2D-pattern (Figure 11a), which is evidence for a layer structure with well defined layers (*d* = 4.8 nm at 160 °C). From the position of the maxima of the outer diffuse scattering at approximately 115 and 245° in the χ scan (Figure 11b), an average tilt angle of about 25° towards the layer normal was found.

**Figure 12:**
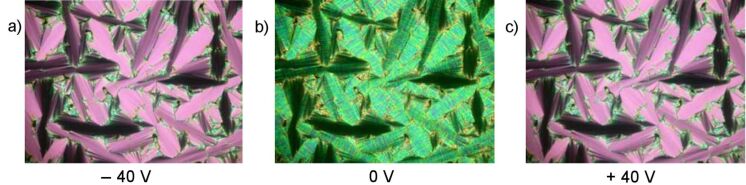
Compound **OH 3b**: Texture of the SmCP_A_ phase in dependence on the polarity of the applied D.C. field.

Initial electro-optical investigations employing an A.C. field (*U* = 260 V_pp_, *f* = 20 Hz, *T* = 106 °C, *d*_cell_ = 6 μm, *P*_S_ = 180 nC/cm^2^) yielded a current response with one repolarization peak per half period only. This would normally be a hint for ferroelectric properties of compound **OH 3b**, but it is in contradiction to the optical behaviour shown in Figure 12. A tristable switching process is observed, and after the electric field is switched off the texture is clearly changed (Figure 12b). In the case of a ferroelectric response, the compound would switch between the states (Figure 12a and Figure 12c), and after the field is switched off one of these textures would be remain. By extensive electro-optical studies employing lower frequencies and a modified triangular wave voltage, the current response peaks split. Thus, the results of the electro-optical studies are consistent with each other, and the mesophase of compound **OH 3b** can be assigned as SmCP_A_ phase.

### Mesophase behaviour of the compounds **OH 4**

The carboxylic groups attached to the central phenyl ring of the molecules **OH 4** have an inverse direction in comparison to those of the compounds **OH 3**. The basic series **OH 4**, i.e., without lateral substituents, has been an object of interest for several research groups [27,37–41]. Some time ago, the influence of lateral substituents on six complete homologous series, in which the aromatic five-ring core corresponds to formula **OH 4**, was investigated by us. The effect of atoms and groups laterally attached to different positions on the central phenyl ring was studied [42]. Therefore, Table 4 shows only a few examples of interest for the present paper.

**Table 4:** Mesophase behaviour, transition temperatures (°C) and -enthalpies [kJ/mol] of compounds **OH 4** (on the right) and corresponding compounds **H 4** (on the left).

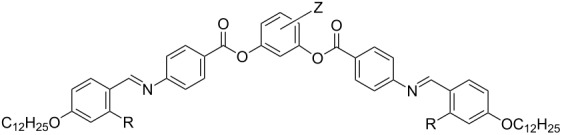													

Mesophase behaviour					R = H	Z	R = OH	Mesophase behaviour					*P*_S_^c^

Cr	124	(SmCP_A_	110)	I	**H 4a**	H	**OH 4a** [27]	Cr	114	SmCP_A_	179	I	500
	[73.4]		[—^a^]						[21.1]		[21.6]		
Cr	88	N	95	I	**H 4b**	4-Br	**OH 4b**	Cr	112	USmCP_A_	146	I	600
	[37.3]		[21.5]						[18.0]		[12.2]		
Cr	125	(B_X_	116)^b^	I	**H 4c**	2-Me	**OH 4c**	Cr	148	SmCP_A_	171	I	640
	[80.9]		[—^a^]						[36.3]		[22.1]		
Cr	98	I			**H 4d**	5-OMe	**OH 4d**	Cr	134	(SmCP_A_	127)	I	590
	[45.7]								[73.2]		[20.1]		

^a^could not be found by calorimetric measurements due to crystallization. ^b^B_X_ phase: Preliminary assignment as crystal-like phase. ^c^in [nC/cm²]

Compounds **H 4a–d** and **OH 4b** and **4d** are new materials. As seen from Table 4, the nature of the mesophase changes upon introduction of the hydroxy groups, from a crystal-like B_X_ phase to a SmCP phase (**H 4c** to **OH 4c**) and from a nematic phase to an undulated SmCP phase (**H 4b** to **OH 4b**). In the case of the 5-methoxy-substituted compound **OH 4d**, liquid-crystalline behaviour was induced by introduction of the hydroxy groups. The increase of the clearing temperatures by the introduction of two ortho-hydroxy groups in compounds **OH 4** depends strongly on the pattern of substitution; compare compounds **H 4a–d** with **OH 4a–d.** The increase amounts to 69 K for the laterally nonsubstituted compounds **H 4a/OH 4a**, but is reduced to 33 K for 4,6-dichloro-substituted compounds (see Supporting Information File 1, Table S4).

**Figure 13:**
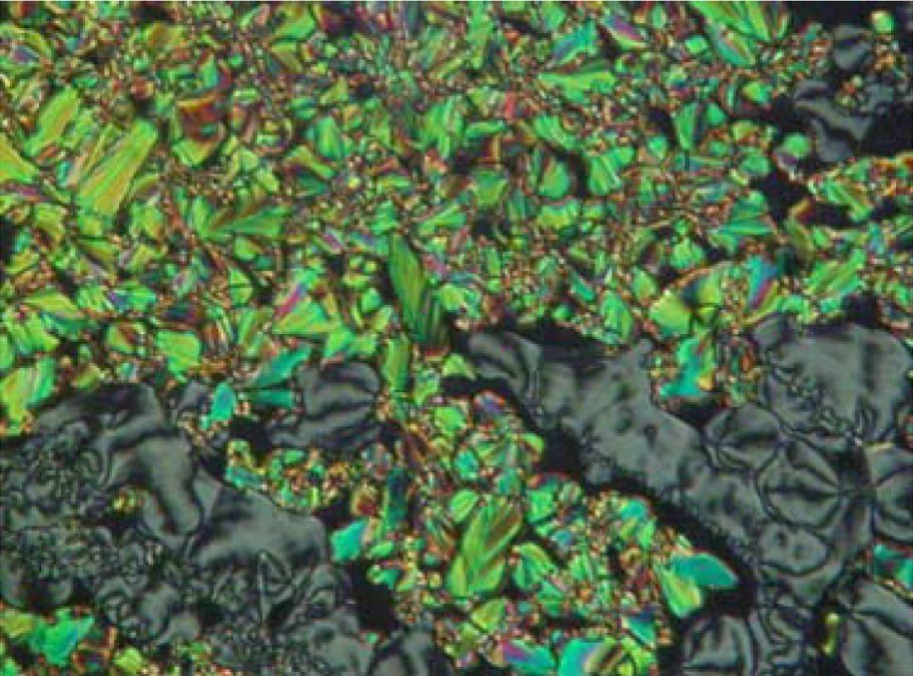
Compound **OH 4b,** exhibiting a fan-shaped texture together with a Schlieren texture upon cooling of the isotropic liquid, *T* = 145 °C.

The 4-bromoresorcinol derivative **OH 4b** (Z = 4-Br) is isomeric to the 4-bromoisophthalic acid ester **OH 3b**. The texture of the mesophase of compound **OH 4b** is quite different from that of **OH 3b**. It shows spherolitic, lancetlike filaments and regions of a growing Schlieren texture upon cooling the isotropic liquid (Figure 13).

**Figure 14:**
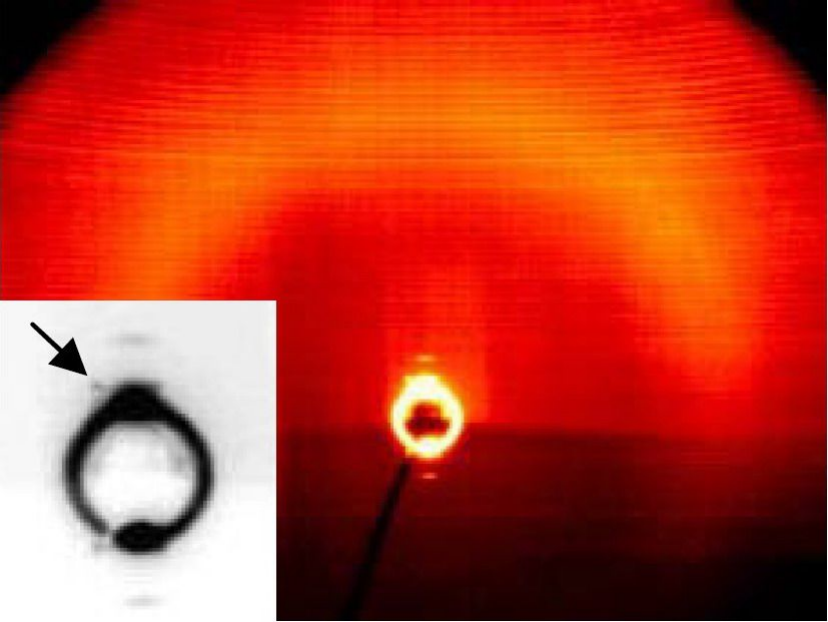
2D X-ray diffraction pattern of a partially surface-aligned sample of **OH 4b** at 115 °C (inset: Small angle region, the arrow points to one of the additional non-layer reflections).

In addition, the X-ray patterns show significant differences in the structures of the mesophases for both compounds. Partially surface-aligned samples of **OH 4b** show additional very weak X-ray reflections next to the first-order layer reflections (Figure 14), which suggest a two-dimensionally modulated electron density, i.e., an undulated or modulated layer structure (layer spacing *d* = 4.5 nm). The position of the reflections implies an oblique 2D lattice for this modulation, but only partial alignment was achieved, and the very strong ringlike-layer reflection affects the measurements of the other very weak ones. Therefore, further quantitative conclusions are rather speculative. One plausible interpretation of the data is given in Supporting Information File 1, Figure S2.

**Figure 15:**
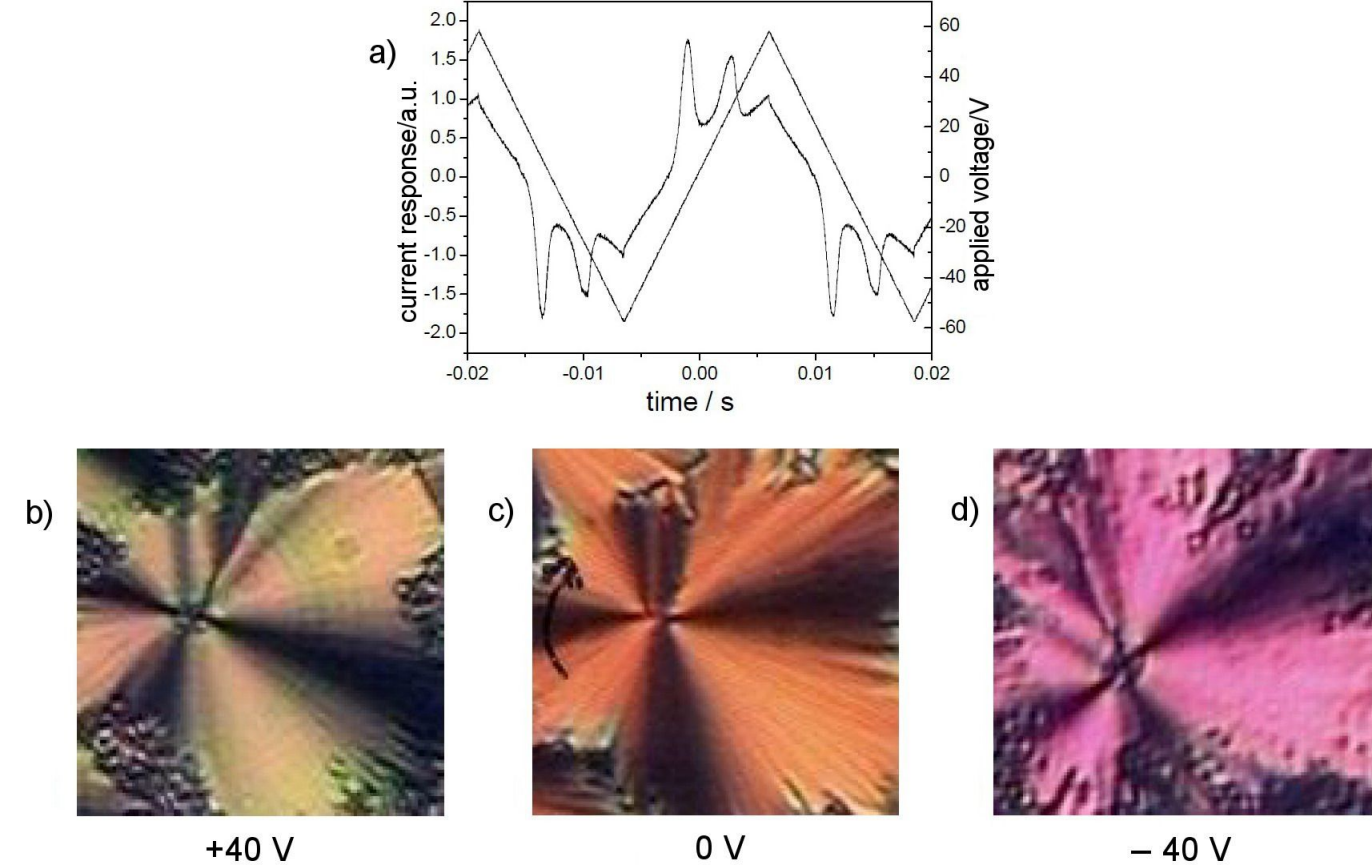
Switching behaviour of compound **OH 4b** at 120 °C: a) Current response curve (*U* = 116 V_pp_, *f* = 40 Hz, *T* = 120 °C, *d*_cell_ = 6 μm, *P*_S_ = 600 nC/cm^2^); b) tristable switching observed on circular domains applying a D.C. voltage (*U* = ± 40 V, *T* = 122 °C, *d*_cell_ = 6 μm).

The designation as a polar phase results from electro-optical investigations. Two repolarization peaks per half period of an applied triangular wave voltage are generated (Figure 15a). The spontaneous polarization amounts to 600 nC/cm^2^. The extinction crosses rotate clockwise (Figure 15b) or anticlockwise (Figure 15d) depending upon the polarity of the electric field, by an angle of about 20°. According to the electro-optical switching behaviour and the results of the X-ray measurements, the mesophase was assigned as a USmCP_A_ phase

The 2-methyl-substituted compound **OH 4c** is isomeric with **OH 5b**, which exhibits an interesting multistage switching [44]. However, electro-optical studies on **OH 4c** show two peaks in the current response, as usually found for a SmCP_A_ phase.

**Figure 16:**
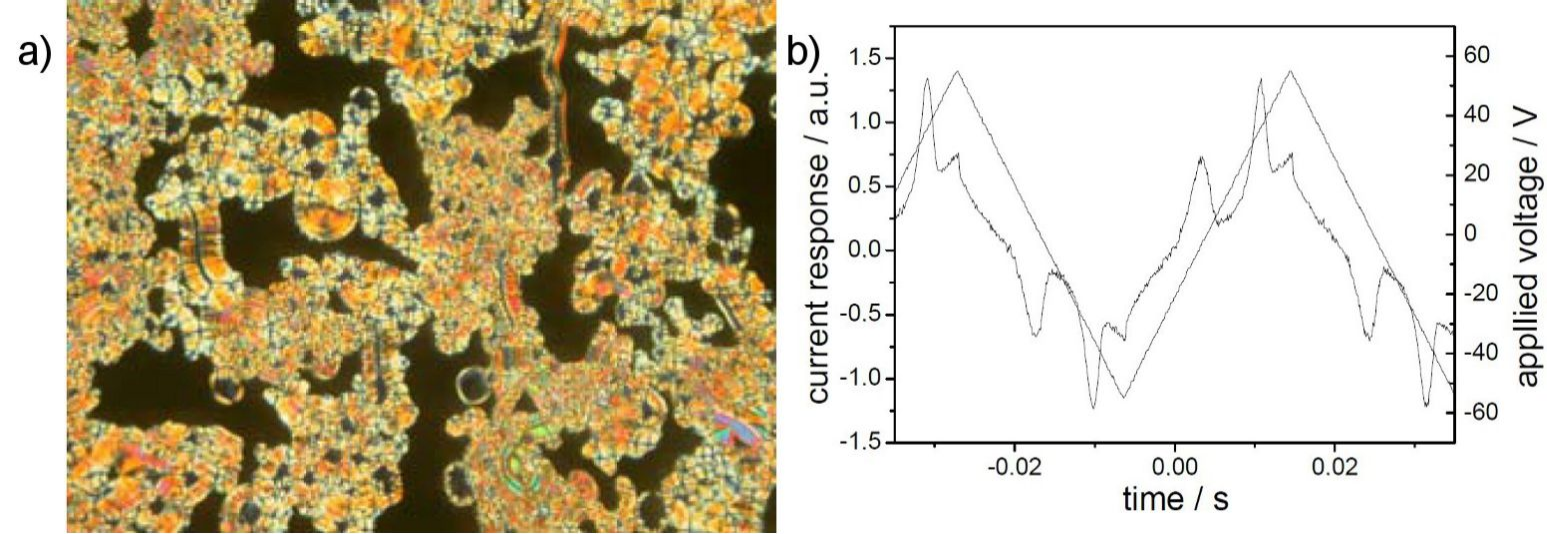
Compound **OH 4d**: a) Microphotograph of a growing fringe pattern of a SmCP phase upon cooling of the isotropic liquid; b) current response curve showing antiferroelectric behaviour (*U* = 19 V/µm, 20 Hz, *d*_cell_ = 6 µm, *P_S_* = 500 nC/cm^2^).

In compound **OH 4d** a methoxy group is attached at the top of the central phenyl ring; this 5-position is often sterically unfavourable for the formation of banana phases. Nevertheless, a metastable SmCP phase was observed and investigated. The growth of a fringe pattern typical for a SmCP phase can be seen in Figure 16a.

**Figure 17:**
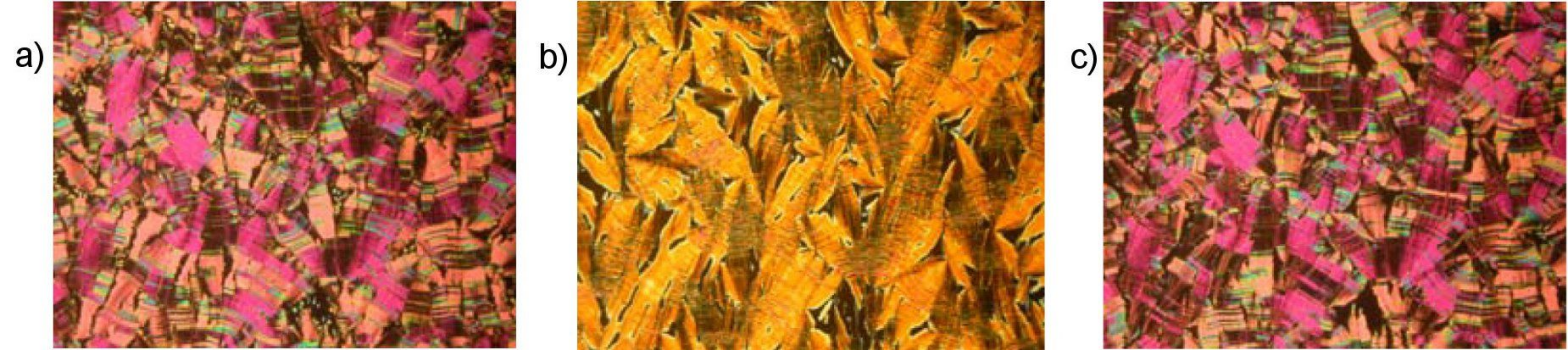
Electro-optical behaviour observed on the fan-shaped texture of compound **OH 4d**; *U*_D.C._ = 47 V; *T* = 123 °C; a) −47 V; b) 0 V; c) +47 V.

The current response shows two peaks per half period, which is typical for an antiferroelectric switching. The spontaneous polarization amounts to 500 nC/cm^2^ (Figure 16b). Action of an electric D.C. field shows a transition from a striped, fan-shaped texture to a smooth one (Figure 17).

**Figure 18:**
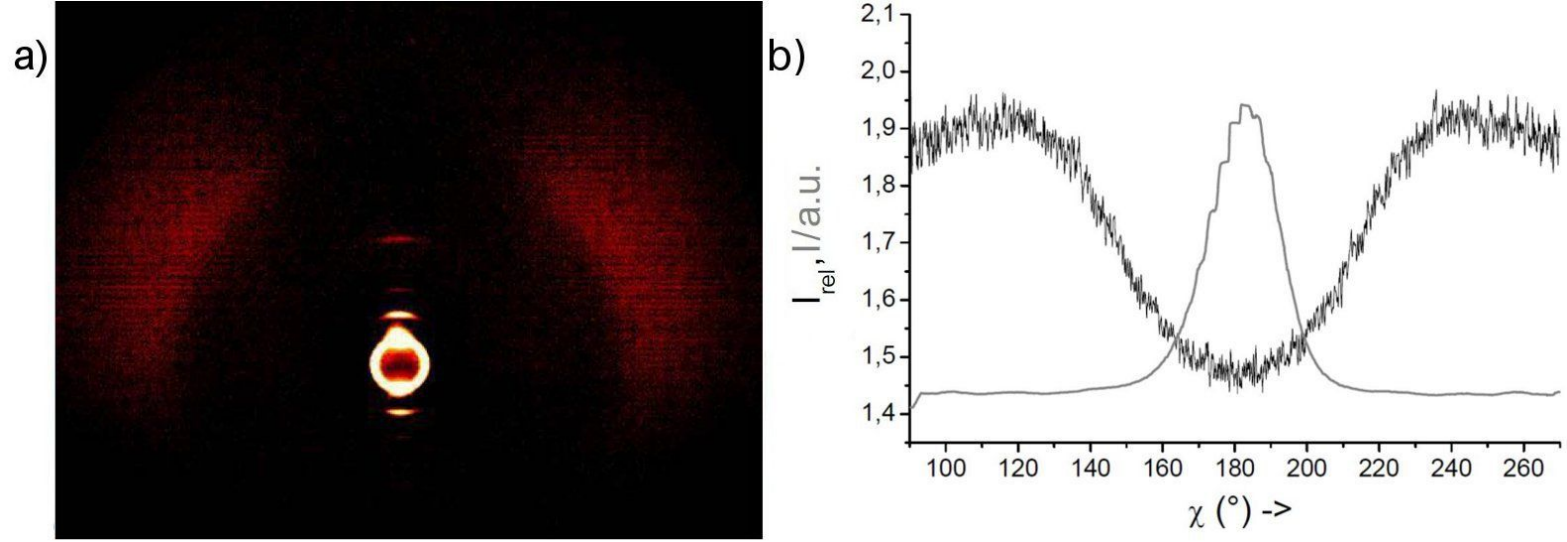
a) 2D X-ray diffraction pattern for a surface-aligned sample of **OH 4d** at 122 °C on cooling; b) χ-scan for the outer diffuse scattering with maxima at about 120 and 240° (*I*_rel_ = *I*(122 °C)/*I*(140 °C, isotropic liquid).

The X-ray diffraction measurements on the monotropic liquid-crystalline phase of **OH 4d** show the patterns of a tilted smectic layer structure with a layer spacing of 4.3 nm and a 30° tilt of the long molecular axes with respect to the layer normal (Figure 18), from which an effective molecular length of *L*_eff_ = *d*/cos θ = 5.0 nm results, which is significantly shorter than the molecular length *L*_calc_ = 5.5 nm estimated by molecular models. The difference may be caused by a deviation of the molecular configuration from that assumed in the model calculations (e.g., by another bending angle or a high proportion of gauche conformers), or by an interdigitation of the terminal chains (see discussion for **OH 4b** in Supporting Information File 1).

The results of electro-optical, microscopic and X-ray diffraction studies prove the existence of a SmCP_A_ phase.

### Disalicylideneimine compounds **OH 5** and **OH 6** bearing the azomethine groups between the central phenyl ring and the neighbouring rings

#### Synthesis of the compounds **OH 5** and **OH 6**

**Scheme 5:**
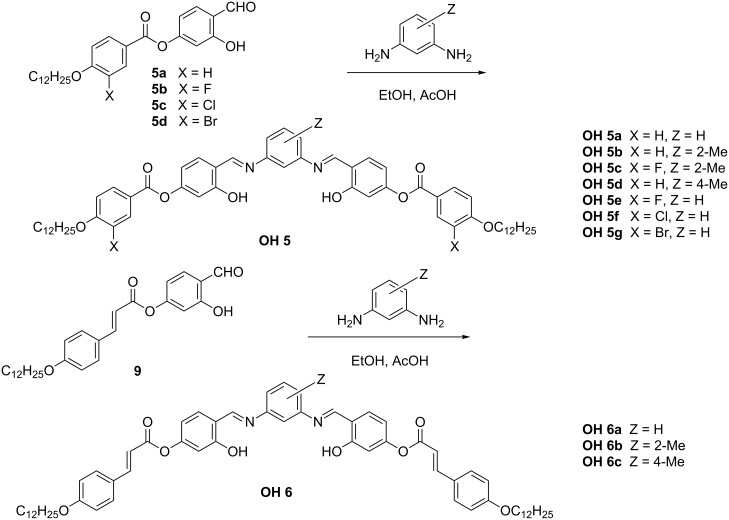
Reaction steps employed for the preparation of the compounds **OH 5** and **OH 6**.

The 1,3-phenylenediamine derivatives **OH 5** and **OH 6** were prepared by condensation of the corresponding 1,3-phenylenediamines with 4-formyl-3-hydroxyphenyl 4-*n*-dodecyloxy-3-substituted-benzoates **5** and 4-formyl-3-hydroxyphenyl 4-*n*-dodecyloxycinnamate (**9**), respectively, as sketched in Scheme 5. Compound **9** was obtained by esterification of 2,4-dihydroxybenzaldehyde with 4-*n*-dodecyloxycinnamic acid, under the same conditions as reported for the salicylaldehydes **5**.

#### Mesophase behaviour of the compounds **OH 5** and **OH 6**

The influence of the pattern of substitution at the outer and/or the central phenyl ring on the phase-transition temperatures and phase type of the compounds **OH 5** and **OH 6** can be seen from Table 5. For comparison, corresponding compounds having the same aromatic core but without the hydroxy groups are given.

**Table 5:** Mesophase behaviour, transition temperatures (°C) and enthalpies [kJ/mol] of compounds **OH 5** and **OH 6** (on the right) and corresponding compounds **H 5** and **H 6** (on the left).

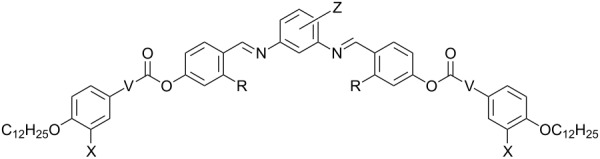														

Mesophase behaviour					R = H	V	X	Z	R = OH	Mesophase behaviour				

Cr	120	(SmCP	114)	I	**H 5a **[52]	—	H	H	**OH 5a**	Cr	177	(Col	172)	I
	[48.9]		[17.1]								[45.2]		[—^a^]	
Cr	128	I			**H 5b**	—	H	2-Me	**OH 5b **[44]	Cr	123	SmCP_A_	193	I
	[26.2]										[71.2]		[19.5]	
							F	2-Me	**OH 5c**	Cr	105	SmCP_A_	206	I
											[63.2]		[20.4]	
Cr	98	I			**H 5d**	—	H	4-Me	**OH 5d**	Cr	97	B_X_	126	I
	[58.0]										[8.5]		[32.1]	
Cr	127	(SmCP	124)	I	**H 5e**	—	F	H	**OH 5e**	Cr	179	USmC	186	I
	[75.1]		[8.6]								[25.9]		[26.7]	
Cr	116	(SmCP	108)	I	**H 5f**	—	Cl	H	**OH 5f**	Cr	165	USmC	173	I
	[74.1]		[13.5]								25.1		[22.2]	
Cr	79	SmCP	92	I	**H 5g**	—	Br	H	**OH 5g**	Cr	143	USmC	161	I
	[14.1]		[6.2]								[17.6]		[13.5]	
Cr	146	I			**H 6a**	HC=CH	H	H	**OH 6a**	Cr	189	(SmCP_A_	183)	I
	[56.8]										[54.4]		[—^a^]	
Cr	136	N	140	I	**H 6b**	HC=CH	H	2-Me	**OH 6b**	Cr	131	SmCP_A_	202	I
	[67.6]		[0.5]								[74.1]		[16.9]	
Cr	107	I			**H 6c**	HC=CH	H	4-Me	**OH 6c**	Cr	114	SmCP_A_	134	I
	[40.8]										[23.4]		[14.4]	

^a^Could not be found by calorimetric measurements due to crystallization.

The phase-transition behaviour shown in Table 5 demonstrates the strong influence of the ortho-hydroxy groups on the mesophase type and the mesophase stability. The comparison of the compounds **H 5a–g** with **OH 5a–g** shows that in all cases the clearing temperatures are increased by about 60 K or liquid-crystalline behaviour is induced.

Compound **OH 5a** exhibits a very high melting point; the occurrence of the metastable columnar phase is established by means of typical textures only. It should be mentioned that the B_6_ banana phase was first reported for short-chain members of the homologous series of **H 5a** [52]. The effect of lateral substituents X and Z is remarkable. Introduction of a methyl group into the 2-position of the central phenyl ring of **OH 5a** not only decreases the melting temperature by 54 K but also increases the clearing point by 21 K for **OH 5b**. Additional attachment of fluorine atoms at the outer phenyl rings increases the smectic–isotropic phase-transition temperature again by 13 K for **OH 5c**. That means that the laterally substituted compound **OH 5c** exhibits a transition temperature to the isotropic phase that is 34 K higher than the laterally unsubstituted parent compound **OH 5a**. This is a really exciting result.

**Figure 19:**
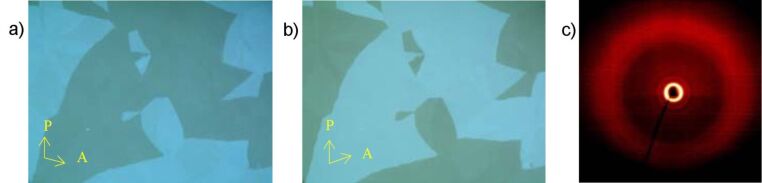
Compound **OH 5d**: Optical photomicrographs of chiral domains at 122 °C; polariser and analyser are uncrossed by about a) −8° and b) +8° from the 90° position; c) X-ray pattern of a powderlike sample at 110 °C.

Upon shifting of the methyl groups of **OH 5b** from the 2-position into the 4-position, the clearing temperature decreases by 67 K for the isomeric compound **OH 5d**. By cooling the isotropic liquid of **OH 5d**, an intensive blue colour arises. Furthermore, chiral domains of different handedness are visible in the texture (Figure 19a and Figure 19b). Both appearances could be a hint for a B_4_ banana phase [8,53–54]. X-ray patterns show slightly broadened layer reflections (*d* = 4.0 nm) and additional weak and sharp reflections together with broadened ones over the entire diffraction range (Figure 19c), which are also arguments for a B_4_ or another soft-crystal-like B_X_ phase of **OH 5d**.

**Figure 20:**
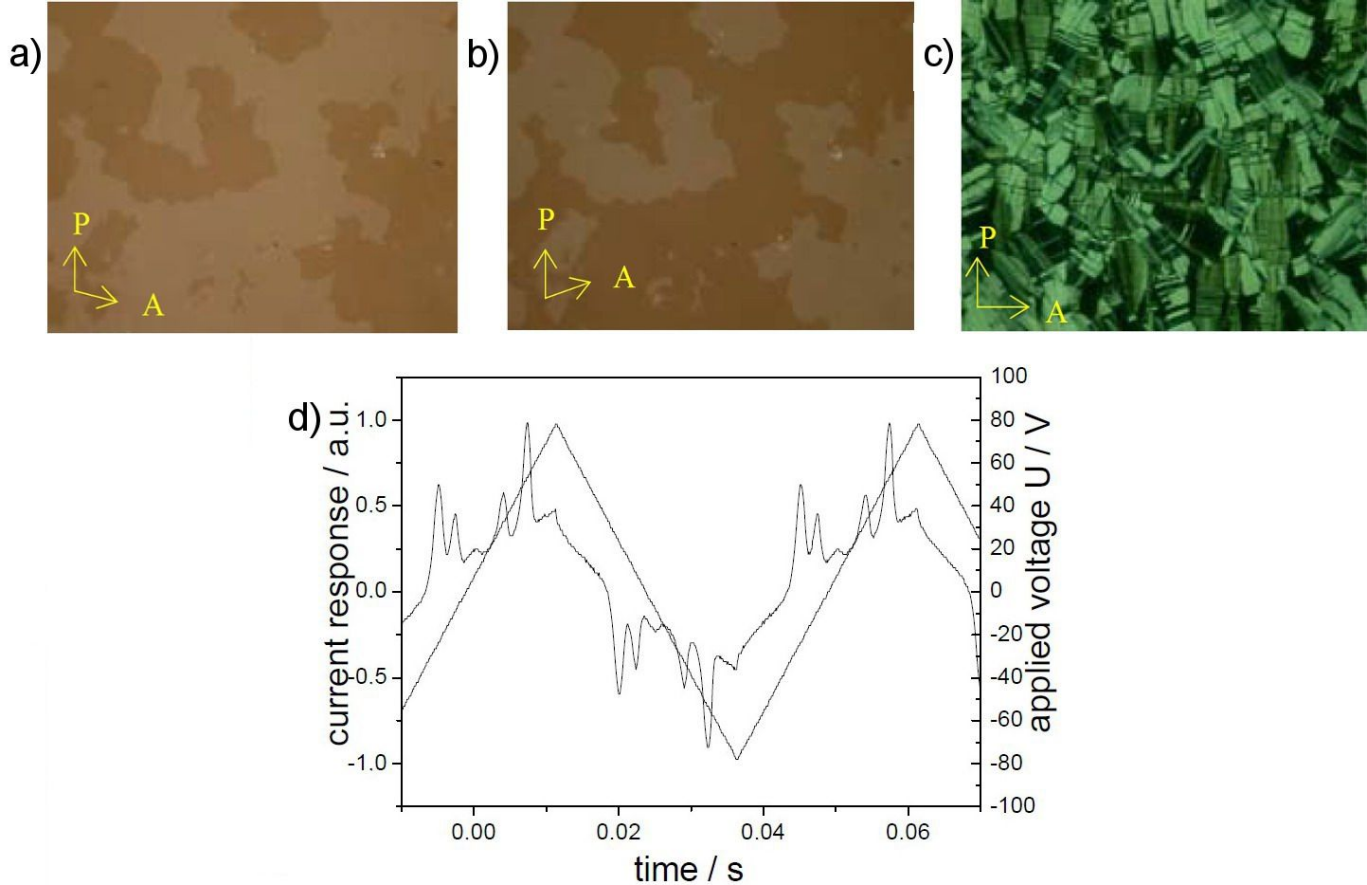
Compound **OH 5b:** a), b) Chiral domains at 175 °C, 0 V, polarisers uncrossed by about ±8° from the 90° position; c) birefringent texture formed under an electric field (*U* = 100 V_A.C._); d) current response (*U* = 158 V_pp_, *f* = 20 Hz, *T* = 174 °C, *d*_cell_ = 6 μm).

The methyl-substituted compound **OH 5b** is of special interest. The X-ray patterns of nonoriented samples show a strong reflection in the small-angle region with a very weak second order reflection and a diffuse scattering in the wide-angle region indicating a simple layer structure without in-plane ordering. The layer spacing of 3.8 nm is clearly smaller than the molecular length *L* = 5.4 nm as estimated by commercial molecular modelling software (Cerius 2) and thus from cosθ = *d*/*L* = 3.8 nm/5.4 nm a tilt angle of about 45° was estimated. Such a tilt angle explains the optical behaviour. Upon cooling of the isotropic liquid, the SmCP phase occurs as nonbirefringent black texture. After slight decrossing of the polarisers, however, optically active domains become visible, as shown in Figure 20a and Figure 20b. Under the action of an A.C. electric field, nucleation of a birefringent texture starts, which coalesces to a non-specific or broken fan-shaped texture (Figure 20c). This state of compound **OH 5b** exhibits an interesting current response consisting of five or more peaks per half period (Figure 20d).

Such a multistage switching is unique for bent-core compounds and was reported by us some time ago [44]. This switching cannot be explained by using a simple layer structure of the SmCP phase. Therefore, a structure model for an intermediate field-induced ferrielectric state was proposed [45]. In the present work it is helpful to search for further bent-core compounds exhibiting a multistage switching. Hence, the molecular structure was changed in a stepwise manner. Starting from compound **OH 5b**, the attachment of fluoro atoms on the outer rings results in compound **OH 5c**, which also exhibits a SmCP_A_ phase. However, a two-peak current response typical of an antiferroelectric switching is found. As mentioned above, compound **OH 5d** isomeric with **OH 5b**, shows a crystal-like phase only. That means that simple changes of the molecular structure can prevent the unusual multistage switching found for **OH 5b**.

Substitution by fluorine, chlorine, and bromine atoms at both outer phenyl rings of compound **OH 5a** results in **OH 5e–g**. The following tendency of clearing temperatures is found: X = H (172 °C), F (186 °C), Cl (173 °C), Br (161 °C). It is very surprising that compound **OH 5f**, bearing two chlorine atoms in lateral positions, exhibits mesophase stability similar to the laterally unsubstituted parent compound **OH 5a**.

**Figure 21:**
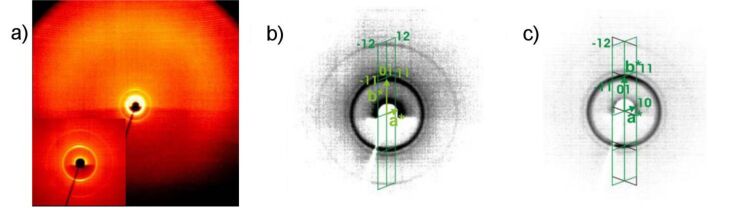
X-ray diffraction patterns of compounds **OH 5**: a) Pattern of a powderlike sample of compound **OH 5g** at 150 °C on cooling (inset: small-angle region); b) small-angle pattern of **OH 5g** showing the reciprocal 2D lattice and the indexing of the observed reflections; c) small-angle pattern of a partially surface-aligned sample of compound **OH 5f** at 158 °C on cooling, showing the two preferred orientations of the reciprocal 2D lattice in the sample and the indexing of the observed reflections.

X-ray diffraction patterns for the halogen-substituted compounds **OH 5e–g** show several sharp small-angle reflections and a diffuse outer scattering (Figure 21). The sample of **OH 5f** was partially surface-aligned and the diffraction angles of four reflections were determined. Thus the reflections were indexed to an oblique 2D lattice (for parameters see Table 6). In the same way the small-angle reflections of the other two compounds can be interpreted. Comparing the molecular volume with the volume of a hypothetical 3D unit cell with a thickness of 0.52 nm, corresponding to the assumed stacking distance of the molecules in bending direction, the number of molecules in the cross section of the unit cell, i.e., in the blocks of the modulated structure, was calculated (see Table 6 and Supporting Information File, Table S3). These structural parameters for the liquid-crystalline phases of the three compounds closely resemble each other. There are several packing models for the molecules that fit these data, namely the undulated-layer model (USmC phase), the broken-layer (columnar) model (Col_ob_ phase), and the polarization-modulation model, and several modes within these models depending on the direction of the polarization between the layers, which cannot be decided based on our X-ray results [5,9]. Since no sufficient alignment of the samples was achieved, the tilt angle of the molecules with respect to the axes of the 2D lattice could not be determined.

**Table 6:** Structural parameters for the modulated-layer phases of compounds **OH 5e**, **5f** and **5g:** Temperature, *T* (°C) on cooling, 2D lattice parameters *a*, *b* (nm), and γ (°), molecules in the cross section of the unit cell *n*_lc_.

Compound	*T*	*a*	*b*	γ	*n*_lc_

**OH 5e**	177	14.0	4.9	116	22
**OH 5f**	158	14.8	4.5	112	21
**OH 5g**	150	17.0	4.4	112	24

**Figure 22:**
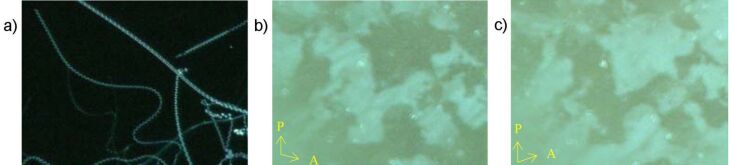
Photomicrographs of compound **OH 5f**: a) Appearance of spiral filaments on slow cooling of the isotropic liquid at 171 °C; b), c) cooling from the isotropic phase in the presence of an electric field of 15 V/μm results in a texture with chiral domains, which are visible by rotation of one polariser clockwise or anticlockwise by several degrees from the crossed position; *T* = 165 °C.

In principle the appearance of spiral germs upon slow cooling of the isotropic liquid of the compounds **OH 5** hints at an undulated smectic phase or a B_7_ banana phase, see Figure 22a. However, in the presence of an electric D.C. field, a texture with chiral domains is observed, with a change in contrast seen upon rotation of one polariser from the 90° position by several degrees (Figure 22). Surprisingly, it was not possible to find a current response typical for a polar banana phase. The birefringence of the sample changed depending on the power of the electric field. Although the chemical structure and the molecular shape suggest that the compounds might be capable of forming banana phase(s), the polar character of the mesophases could not be unambiguously determined. Therefore, the mesophases of the compounds **OH 5e–g** are designated as USmC phases.

A further slight modification of the chemical structure of the series **OH 5** is the insertion of C=C groups between the outer and the neighbouring phenyl rings, giving **OH 6a–c**. Of all three hydroxy group-free compounds, **H 6a–c**, only the 2-methyl-substituted compound shows liquid-crystalline behaviour. A nematic phase is observed, which is very surprising for this mesogen having a molecular shape characteristic of banana-shaped liquid crystals. Although both legs of the bent-core molecules are extended by the C=C units, the clearing temperatures increase only by about 10 K. Introduction of a lateral methyl group on the central phenyl ring drastically decreases the melting temperatures. Therefore, broad SmCP phase ranges exist for **OH 6b** and **OH 6c** in comparison to the laterally unsubstituted derivative **OH 6a**. That also means that the nematic phase of **H 6b** changes to a polar smectic phase in **OH 6b** by insertion of the C=C units.

**Figure 23:**
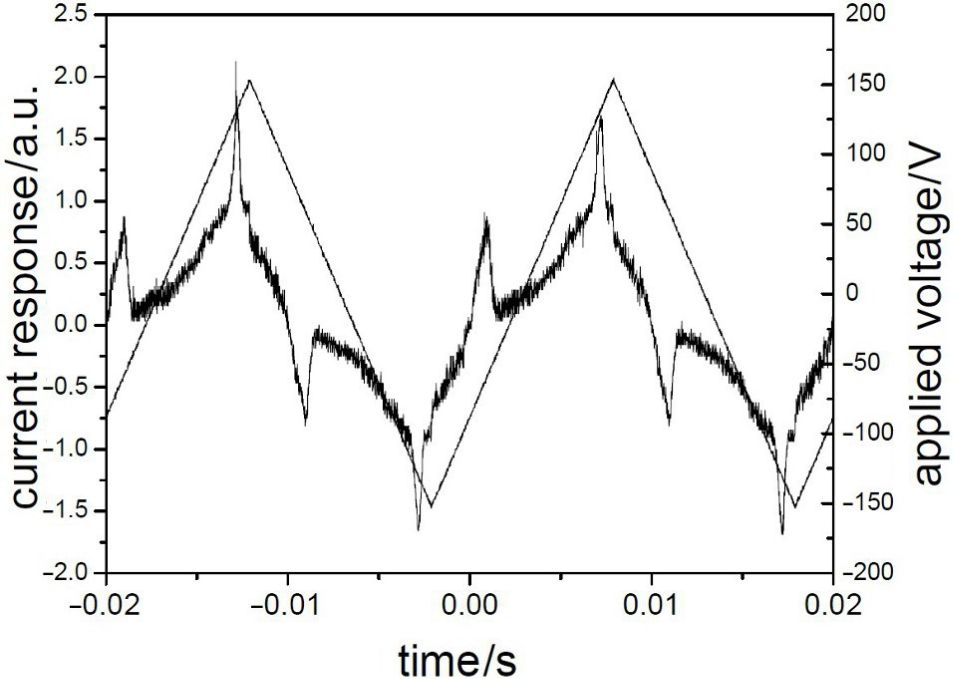
Current response of compound **OH 6b** exhibiting two repolarisation peaks proving an antiferroelectric switching (*U*_A.C._ = 15 V/µm, *f* = 50 Hz, *T* = 174 °C, *d*_cell_ = 6 μm).

The mesophase behaviour of compound **OH 6b** is characteristic for a SmCP_A_ phase. On cooling, the isotropic liquid fringe textures as well as Schlieren textures are observed. The current response shows two peaks per half period (Figure 23). The spontaneous polarization amounts to 220 nC/cm^2^. However, notably, the multistage switching found for the corresponding compound **OH 5b** disappears.

**Figure 24:**
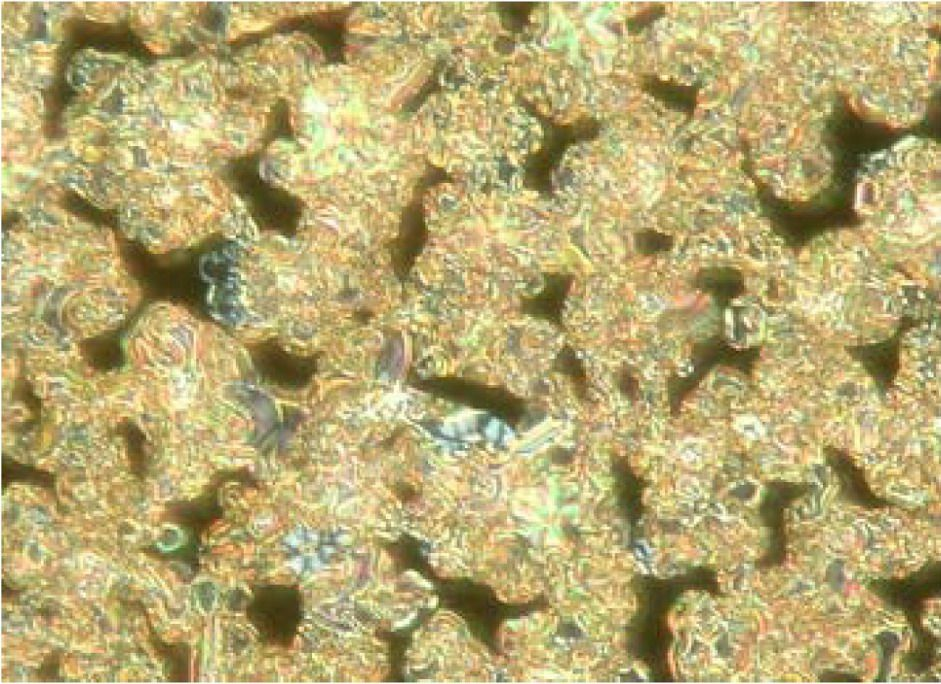
On cooling the isotropic liquid phase of compound **OH 6c**; a Schlieren texture together with fringe pattern appears characteristic for SmCP_A_ phases.

By shifting the methyl group from the 2-position in **OH 6b** to the 4-position of the central phenyl ring of compound **OH 6c**, the clearing temperature decreases by 68 K. Nevertheless, an enantiotropic mesophase exists and can be investigated. Microscopic pictures show textures typical for SmCP phases (Figure 24).

**Figure 25:**
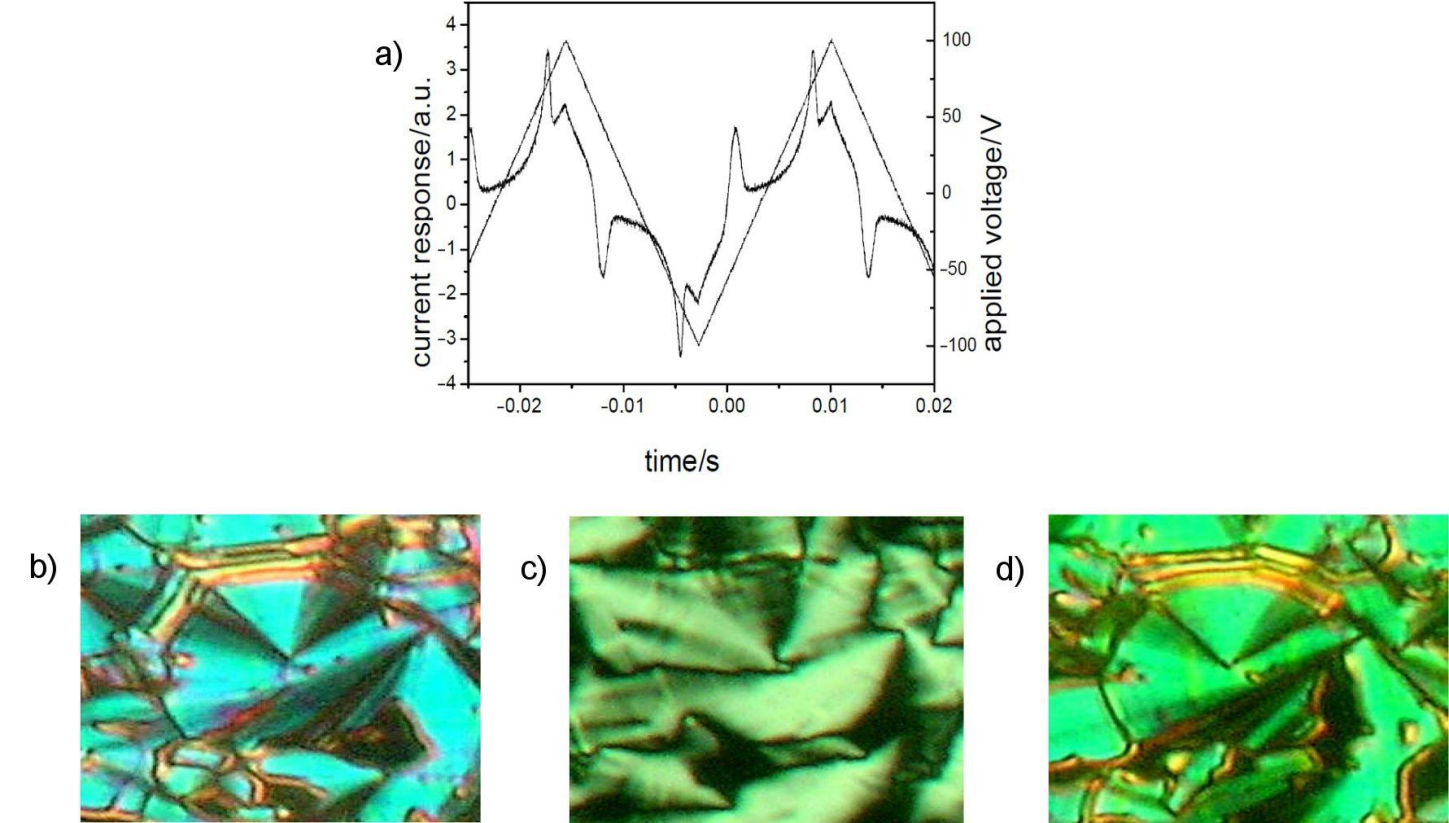
Electro-optical behaviour of compound **OH 6c**: a) Current response (*U*_A.C._ = 17 V/µm, *f* = 39 Hz, *T* = 123 °C, *d*_cell_ = 6 μm, *P*_S_ = 520 nC/cm^2^); c) 0 V, ground state SmC_a_P_A_; b) and d) formation of the SmC_s_P_F_ phase upon application of a D.C. field of different sign: b) +7 V/µm; d) −7 V/µm.

Guinier X-ray patterns of a powderlike sample of **OH 6c** show first and second order layer reflections and a diffuse outer scattering indicating a smectic layer structure with a layer spacing of 4.0 nm. Electro-optical studies of the mesophase of compound **OH 6c** support the assignment as a SmCP_A_ phase. Figure 25 shows the current response curve and microscopic pictures, which demonstrate the switching between SmC_a_P_A_ to SmC_s_P_F_ upon application of a D.C. electric field.

## Conclusion

The use of salicylideneaniline moieties in five-ring bent-core mesogens is shown to be helpful in deriving the relationship between the chemical structure and the mesophase behaviour. Here, the effect of fluorine, chlorine, and bromine atoms laterally attached to the outer phenyl rings, the influence of the hydroxy group in ortho-position to azomethine units, and the effect of a methyl group situated at the central phenyl ring on the liquid-crystalline behaviour and physical properties were studied.

For most of the salicylideneaniline compounds synthesized, the corresponding benzylideneaniline derivatives were prepared for a comparative study. As expected, the clearing temperatures generally increase due to the presence of the ortho-hydroxy group. The increase amounts to about 30 K for mono-salicylideneaniline compounds **OH 1** and **OH 2**, and can be up to 60 K for the mesogens containing two salicylideneaniline moieties, namely **OH 4**, **OH 5**, **OH 6**. As shown in Table 1–Table 5 and Supporting Information File 1, Table S4, the definite increase of the clearing temperature can be much lower depending on the size of the molecules, the length of the terminal chains, and the presence of further lateral substituents and their positions at the different phenyl rings. More importantly, in many cases the introduction of the hydroxy group induces liquid-crystalline behaviour, which was not observed for the corresponding benzylideneaniline compounds. There are other cases where the type of mesophase also changed, for example from nematic to SmCP, nematic to USmCP, B_X_ to SmCP, and SmCP to columnar.

The influence of one halogen atom attached at the outer phenyl rings was investigated in several nonsymmetric molecules. The mono-halogen derivatives of three series are isomeric to each other (**OH 1b–c**, **OH 2e–g**, **OH 2h–j**). All these compounds exhibit SmCP phases. The lowering of the clearing temperatures by a lateral fluorine, chlorine, or bromine atom is unexpectedly low and nearly independent of the different chemical structure of the aromatic cores of the compounds **OH 1** and **OH 2**. For example, the clearing temperatures of the isomeric mono-chlorinated compounds (**OH 1b** 140 °C; **OH 2f** 138 °C; **OH 2i** 135 °C) have nearly the same values as those of the corresponding laterally unsubstituted compounds (**OH 1a** 142 °C; **OH 2a** 136 °C) The replacement of the chlorine atom by the larger bromine atom additionally decreases the clearing temperatures by only 2–6 K.

Looking at the compounds **OH 5e–g**, which have two halogen atoms, i.e., one halogen atom at each leg, the lowering of the clearing points found for mono-halogen derivatives is approximately doubled. Nevertheless, the effect of lateral halogen atoms on the mesophase stability is much lower for the salicylideneanilines under study, as reported for comparable derivatives in the original banana series. There, in dependence on further substituents at the central phenyl ring, the clearing temperatures of corresponding terminally dodecyloxy-substituted derivatives decreased by the introduction of chlorine atoms, by 18–34 K. For related octyloxy-substituted homologues the effect was much stronger [55–56]. The reason for such differences found in different series of bent-core mesogens is not clear up to now.

Comparing the compounds **OH 5a–c**, an unusually strong increase of the mesophase stability caused by lateral substituents was found. In comparison to the salicylideneimine-based compound **OH 5a** (clp 172 °C), the clearing point of the 2-methyl-substituted compound **OH 5b** increases to 193 °C; compound **OH 5c** having additional fluorine atoms at the outer phenyl rings exhibits a SmCP–isotropic phase-transition temperature of 206 °C.

In relation to this it should be mentioned that the cinnamic ester **H 6b**, having a methyl group in the same position, exhibits a nematic phase. The formation of nematic and nonpolar smectic phases by bent-core mesogens can be explained by flexible molecular fragments, and especially by a bending angle clearly above 120° [57]. Such molecules have a more rodlike shape, and therefore “calamitic phases” can be formed. The 4-bromoresorcinol derivative **H 3c** also shows a nematic phase. It is known from NMR studies of other related bent-core mesogens that halogen atoms in the 4-position extend the bending angle to about 135°, and hence this would explain the nematic phase in compound **H 3c**. The influence of a methyl group in the 2-position of the central phenyl ring on the bending angle of bent-core mesogens depends on the chemical structure and on the direction of the linking groups, which connect both molecular legs to the central phenyl ring, as we know from solid state NMR studies. For 2-methylresorcinol bisbenzoates, for example, the bending angle amounts to about 120° [58]. In contrast, for isomeric diphenyl 2-methylisophthalates the bending angle is extended to about 145° [59]. Transferring this knowledge to the methyl-substituted compound **H 6b** would mean that the existence of a nematic phase strongly hints at a bending angle larger than 120°. However, the evidence can only be delivered by NMR studies of the liquid-crystalline state.

The electro-optical behaviour of the salicylideneaniline compounds can change in comparison to that of the corresponding compounds without a hydroxy group. Upon action of an electric field on a SmCP phase, the tilted molecules can rotate on a cone or can rotate about their long axis. Only the latter movement changes the chirality of the layers. In a few cases both molecular processes have been observed for the same material, in dependence on the frequency, temperature and/or the field [48,50–51]. For compound **H 1a** both molecular movements exist depending on how rapidly the electric field is removed. This interesting behaviour disappears in compound **OH 1a** by the substitution with an ortho-hydroxy group.

A further motivation for the present work was the exciting multistage switching reported for the 2-methyl-substituted compound **OH 5b** (and for two longer-chain homologues) bearing two salicylidene legs [44]. Therefore, the chemical structure of this molecule was varied step by step. Liquid-crystalline behaviour was not observed for the corresponding compound without an ortho-hydroxy group, **H 5b**. In compound **OH 4c** only the COO and CH=N linking groups are exchanged in comparison to the isomeric compound **OH 5b**; however, the characteristic electro-optical behaviour of a SmCP_A_ phase was found. Furthermore, the chemical structure of compound **OH 5b** was varied by substitution with fluorine atoms at the outer phenyl rings (**OH 5c**), by shifting the methyl group to the 4-position of the central phenyl ring (**OH 5d**), by insertion of C=C units (**OH 6b** and **OH 6c**) and by preparation of a corresponding compound having only one salicylideneaniline moiety together with a methyl group in the 2-position (**OH 2c**). Unfortunately, the creation of new compounds exhibiting a multistage switching was not successful.

The studies on the influence of lateral halogen atoms, and also of a lateral methyl group, on the mesophase behaviour and further physical properties of salicylideneimine-based bent-core mesogens show that the relationships strongly differ from those reported for calamitic liquid crystals, and that they also vary in comparison to other similar five-ring bent-core mesogens.

## Experimental

The thermal transition behaviour was investigated by using a Perkin Elmer DSC Pyris 1 differential scanning calorimeter. Texture observations of liquid-crystal films sandwiched between two glass plates were carried out with a Leitz polarizing microscope (Laborlux 12 Pol S, Germany). For the electro-optical measurements we used commercial sandwich cells (E.H.C., Japan) with thickness of 6 µm, having a rubbed polyimide coating for planar surface alignment. The cells were observed in transmission by using a Leica (DMRXP, Germany) polarizing microscope equipped with a digital camera (Nikon Coolpix 4500, Japan). The cells are mounted on a heating stage (Linkam LTS 350, UK, and Mettler FP82HT and FP90, UK) for temperature control. The magnitude of the spontaneous polarization was measured by integrating the polarization-reversal current peak obtained by switching the sample with a triangular electric field.

X-ray diffraction measurements on powderlike samples in glass capillaries kept in a temperature-controlled heating stage were performed by using a Guinier film camera (Huber Diffraktionstechnik GmbH) and quartz-monochromatized Cu Kα radiation. 2D diffraction patterns were recorded on an area detector (HI-Star, Siemens/Bruker) from Ni-filtered Cu Kα radiation. Surface aligned fibrelike disordered samples were obtained by slowly cooling a drop of the isotropic liquid placed on a glass plate on a temperature-controlled heating stage.

Reaction pathways to prepare the final compounds **OH 1–6** are sketched in Scheme 2–Scheme 5.

Generally, all compounds **H1–H6**, which do not bear the ortho-hydroxy group, were prepared by following the same reaction pathways sketched in Scheme 2–Scheme 5, but instead of the salicylidene intermediates the corresponding benzylidene intermediates were used. Further details are given in Supporting Information File 1.

## Supporting Information

File 1 Schematic presentation of the SmC_a_P_A_ phase, additional X-ray data, mesophase behaviour of selected compounds and synthesis of compounds (experimental procedures and analytical data).
