# Supplementary material for: Laterally substituted symmetric and nonsymmetric salicylideneimine-based bent-core mesogens
Source: Beilstein J Org Chem. 2012 Jan 24;8:129–54. doi: 10.3762/bjoc.8.15 (PMC3302074; doi:10.3762/bjoc.8.15)
Supplement: File 1 — Schematic presentation of the SmCaPA phase, additional X-ray data, mesophase behaviour of selected compounds and synthesis of compounds (experimental procedures and analytical data). [file Beilstein_J_Org_Chem-08-129-s001.pdf]

Supporting Information

for

Laterally substituted symmetric and nonsymmetric salicylideneimine-based bent-core mesogens

Sonja Findeisen-Tandel[1], Wolfgang Weissflog*[1], Ute Baumeister[1], Gerhard Pelzl[1], H. N. Shreenivasa Murthy[1] and Channabasaveshwar V. Yelamaggad[2]

Address: [1]Martin-Luther-Universität Halle-Wittenberg, Institut für Chemie, Physikalische Chemie, von-Dankelmann-Platz 4, 06120, Halle, Germany and [2]Centre for Soft Matter Research, Jalahalli, Bangalore 560 013, India

Email: Wolfgang Weissflog - weissflog@chemie.uni-halle.de

*Corresponding author

Schematic presentation of the SmC_aP_A phase, additional X-ray data, mesophase behaviour of selected compounds and synthesis of compounds (experimental procedures and analytical data).

Table of contents

Schematic presentation of the SmC$_a$P$_A$ phase and its electro-optical switching

Figure S1 Molecular arrangement of bent-core molecules and their electro-optical switching in the SmC$_a$P$_A$ phase

Additional X-ray data

Table S1: X-ray data for the layer reflections from Guinier film measurements (9 compounds)

Table S2: X-ray data for the 2D modulated phases (compounds **OH 4b, OH 5e-g)**

Table S3: Estimation of the number of molecules in the cross section of the 2D unit cells in the modulated smectic phases (compounds **OH 4b, OH 5e-g)**

Figure S2**:** a) Small angle X-ray diffraction pattern for a partially surface-aligned sample of **o-OH 4b;** b) reciprocal and real lattice; c) one possible schematic model of the undulated SmCP phase of compound **OH 4b**.

Mesophase behaviour

Table S4: Mesophase behaviour of additional compounds **OH 4** and corresponding compounds **H 4**

Synthesis of the compounds: Experimental procedures and analytical data

1. General procedures
2. Intermediates
3. Final compounds **OH 1-6 and H 1-6**

Schematic presentation of the SmC$_a$P$_A$ phase and its electro-optical switching

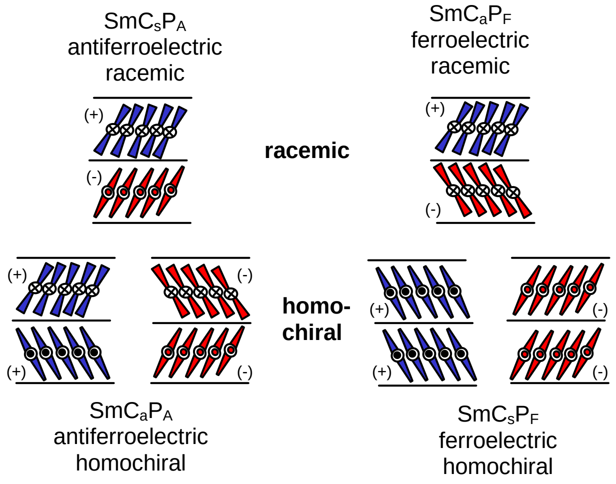

Figure S1: The molecular arrangement of bent-core molecules considering the tilt and the polarity in adjacent layers of the SmCP phase. The smectic layers are perpendicular to the drawing plane. The different molecular symbols correspond to opposite bent directions perpendicular to the drawing plane: Symbol having a circle in the centre means front view; symbols with a cross means back-side view. Both directions of the bend mean also opposite polar axes. The red and blue symbols indicate layers of different chirality. In the upper part, on the left, the racemic antiferroelectric state is sketched, which can be switched to the ferroelectric state on application of an electric field, shown on the right. In the lower part, the homochiral antiferroelectric states are sketched on the left, in which the tilt direction alternates. The field-induced arrangement in the homochiral ferroelectric state is shown on the right.

Polar smectic C phases (SmCP) are designated with the following symbols: **P** stands for *polar*, the suffixes **A** or **F**, added to **P**, indicate *antiferroelectric* or *ferroelectric* states (SmCP$_A$ and SmCP$_F$, respectively). A *synclinic* or an *anticlinic* interlayer correlation is indicated by the suffixes **s** or **a** after C (i.e., SmC$_s$P, SmC$_a$P). In the so-called homochiral (homogeneous chiral) structures all layers have the same handedness, whereas in the racemic state the chirality alternates from layer to layer.

X-ray diffraction measurements

Additional X-ray data

Table S1: X-ray data for the layer reflections from Guinier film measurements (*T* temperature, all measurements on cooling, θ Bragg angle, d_{obs} observed *d* value, *n* order of the layer reflection, *d* layer spacing).

Compound	*T*/°C	θ/°	d_{obs}/nm	*n*	*d*/nm
**OH 1a**	95	1.100	4.01	1	4.01
		2.199	2.01	2	
**OH 1b**	135	1.062	4.16	1	4.16
		2.117	2.09	2	
**OH 2a**	128	1.150	3.84	1	3.84
		2.300	1.92	2	
**OH 3a**	185	0.938	4.71	1	4.70
		1.889	2.34	2	
**OH 3b**#	160	0.920	48.0	1	4.80
		1.829	24.2	2	
		2.776	15.9	3	
		3.680	12.0	4	
		4.624	9.6	5	
**OH 4d**	122	1.029	4.29	1	4.29
		2.063	2.14	2	
**OH 5b**	160	1.150	3.84	1	3.85
		2.293	1.93	2	
**OH 5d***	115	1.101	3.84	1	3.85
		2.201	1.93	2	
**OH 6c**	130	1.112	3.97	1	3.97
		2.227	1.98	2	

# data from 2D SAXD
* data for the two strongest inner reflections

Table S2: X-ray data for the 2D modulated phases (*T* temperature, all measurements on cooling, *a*, *b*, γ lattice parameters, θ observed Bragg angle, d_{obs} observed d value, *h, k* Miller indices, d_{calc} d value calculated from the lattice parameters).

hk	θ/°	d_{obs}/nm	d_{calc}/nm	$d_{obs} - d_{calc}$/nm
**OH 4b*:** *T = 115 °C, a = 6.57 nm, b = 4.82 nm, γ = 111.5°*				
01	*0.986*	*44.8*	*44.8*	*0.00*
02	*1.955*	*22.6*	*22.4*	*0.02*
11	*1.419*	*31.1*	*31.1*	*0.00*
−11	*0.986*	*44.8*	*44.8*	*0.00*
**OH 5e:** *T* = 177 °C, *a* = 14.0 nm, *b* = 4.93 nm, γ = 116.5°				
-11	0.90	4.91	4.90	−0.01
01	1.00	4.42	4.41	0.01
11	1.20	3.68	3.68	0.00
−12	1.90	2.33	2.36	−0.03
**OH 5f:** *T* = 158 °C, *a* = 14.8 nm, *b* = 4.5 nm, γ = 112°				
−11	0.898	4.47	4.49	−0.02
01	1.065	4.15	4.17	−0.02
11	1.225	3.60	3.63	−0.03
−12	2.002	2.21	2.19	0.02
**OH 5g:** *T* = 150 °C, *a* = 17.0 nm, *b* = 4.4 nm, γ = 112°				
−11	1.012	4.36	4.37	−0.01
01	1.082	4.08	4.08	0.00
11	1.212	3.64	3.63	0.01
−12	2.05	2.15	2.13	0.02
12	2.29	1.93	1.93	0.00

* with limited accuracy, see comments to Figure S1

Table S3: Estimation of the number of molecules in the cross section of the 2D unit cells in the modulated smectic phases (*T* temperature, $V_{M,cr}$ molecular volume in the crystal calculated by the incremental method of A. Immirzi, B. Perini, *Acta Cryst.* 1977, A33, 216 – 218, $V_{M,l}$ molecular volume in the isotropic liquid derived from $V_{M,cr}$ by the ratio of the average packing coefficients in both phases $V_{M,l} = V_{M,cr}k_{cr}/k_l$ with $k_{cr} = 0.7$ and $k_l = 0.55$ according to A. I Kitaigorodski, “Molekülkristalle”, Akademieverlag Berlin, 1979, V_U ... volume of a hypothetical 3D unit cell calculated from the lattice parameters and a height *h* of about 0.52 nm corresponding to the assumed stacking distance of the molecules in bend direction by $V_U = a{\cdot}b{\cdot}h{\cdot}\sin\gamma$, n_{cr} number of molecules in the cross section of the unit cell for a crystal-like packing density, n_l number of molecules in the cross section of the unit cell for a liquid-like packing density, n_{lc} number of molecules in the cross section of the unit cell in the liquid-crystalline state, estimated as the average of n_{cr} and n_l).

Compound	*T*/°C	$V_{M,cr}$/nm^3	$V_{M,l}$/nm^3	V_U/nm^3	n_{cr}	n_l	n_{lc}
**OH 4b**	115	1.318	1.677	15.321	11.6	9.1	10.4
**OH 5e**	177	1.310	1.668	32.092	24.5	19.2	21.9
**OH 5f**	158	1.338	1.703	32.110	24.0	18.9	21.4
**OH 5g**	150	1.351	1.718	36.064	26.7	21.0	23.8

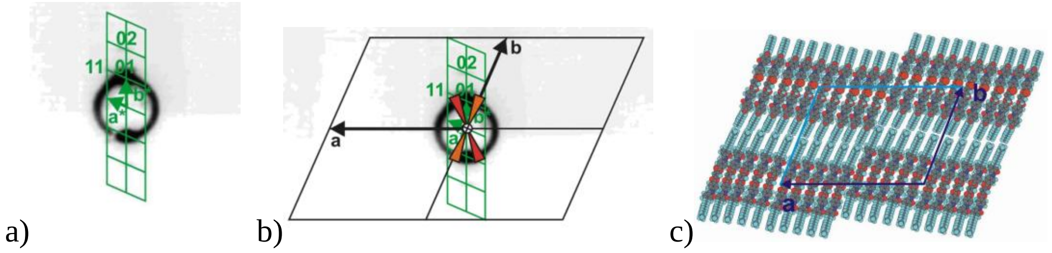

Figure S2: a) Small angle X-ray diffraction pattern for a partially surface-aligned sample of **OH 4b** at 115 °C, indexed on the basis of an oblique lattice with the parameters a = 6.6 nm, b = 4.8 nm, γ = 111.5°; b) reciprocal (green) and real lattice (black) with the possible orientation of the molecules in the undulated SmCP phase; c) one possible (schematic) model of the undulated SmCP phase with an orientation of the molecular long axis along lattice direction b (corresponding to the orange “molecule” in b).

One possible interpretation of the X-ray data for **OH 4b** in agreement with the other measurements is the following:
The additional reflections may be indexed as a 11 reflection of an oblique 2D lattice. Assuming the -11 reflection to be superimposed by the very strong ring-like 01 layer reflection, lattice parameters can be determined to a = 6.6 nm, b = 4.8 nm and γ = 111.5°. Comparing the volume V_U = 15.32 nm^3 of a hypothetical 3D unit cell with the molecular volume in the liquid crystalline phase $V_{M,lc}$ = 1.49 nm^3, about 10 molecules occupy the cross section of the unit cell (cp. Table S3). From the azimuthal distribution of the wide angle scattering along (χ-scan), the tilt angle θ of the molecules with respect to the layer normal can be determined to be ~20°, which is in good agreement with the optically determined value. From this tilt and the d-value of the 01 reflection (layer thickness 4.5 nm) an effective molecular length of L_{eff} = 4.8 nm can be calculated according to $L_{eff} = d/\cos\theta$. Using CPK models and with the assumptions that the bending angle amounts to nearly 120° and that all chains are in an all-trans configuration, a length L_{calc} of 5.46 nm would be obtained. The difference (L_{eff} = 4.8 nm and L_{calc} = 5.5 nm) indicates that the molecular configuration assumed in this calculation is not correct for compound **OH 4b**. Another bending angle, a high portion of gauche conformers, or an interdigitation of the terminal chains could be the reason for the difference. The relatively large bromine atom at the central part of the molecule requires an increased lateral space, which could make easier an interdigitation of hydrocarbon chains of adjacent layers.

Materials

Table S4: Transition temperatures (°C) and enthalpies [kJ/mol] of compounds **OH 4** (R = OH; on the right) and corresponding compounds **H 4** (R = H, on the left).

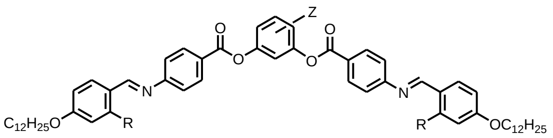

Mesophase behaviour	R = H	Z	R = OH	Mesophase behaviour	ΔT/K (Klp.OH-Klp.H)
Cr 124 ($SmCP_A$ 110) I<br>[73.4]	**H 4.1**	H	**OH 4.1**	Cr 114 $SmCP_A$ 179 I<br>[21.1] [21.6]	69
Cr 96 N 101 I	**H 4.2**<br>[13]	4-Cl	**OH 4.2**	Cr 112 $USmCP_A$ 156 I<br>[20.5] [14.3]	55
Cr 88 N 95 I<br>[37.3] [21.5]	**H 4.3**	4-Br	**OH 4.3**	Cr 112 $USmCP_A$ 146 I<br>[18.0] [12.2]	51
Cr 90 N 124 I	**H 4.4**<br>[13]	4,6-Cl	**OH 4.4**	Cr 133 SmC 153 N 157 I<br>[55.2] [1.2] [2.5]	33
Cr 125 (B_4 116) I<br>[80.9]	**H 4.5**	2-Me	**OH 4.5**	Cr 146 $SmCP_A$ 169 I<br>[36.3] [22.1]	53
Cr 98 [45.7] I	**H 4.6**	5-MeO	**OH 4.6**	Cr 134 ($SmCP_A$ 127) I<br>[73.2] [20.1]	>29
–	–	5-Me	**OH 4.7**<br>[4]	Cr 136 $SmCP_A$ 153 I<br>[13.6] [22.9]	–
Cr 117 B_7 122 I<br>[30.2] [24.5]	**H 4.8**<br>[14]	2-NO_2	**OH 4.8**<br>[4]	Cr 117 B_7 170 I<br>[20.3] [24.1]	48
–	–	4-NO_2	**OH 4.9**<br>[4]	Cr 95 $SmCP_A$ 183 I<br>[6.9] [19.6]	–

Synthesis of the compounds: Experimental procedures and analytical data

The preparation of the final compounds is sketched in the Schemes 2–5 (main paper). Because several reactions were performed by using similar experimental conditions, three general procedures are given here. In the second part new intermediates are described. In the third part the preparation of the final products is reported, the melting temperature and the mesophase behaviour are summarised in the corresponding Tables 2–5 (main paper).

1. General procedures

General procedure 1 (GP1):
Esterification of benzoic acids with phenols by the carbodiimide method
The substituted benzoic acid (0.005 mol), the corresponding phenol (0.005 mol) and a catalytic amount of DMAP were dissolved in 50 mL of dry dichloromethane. Dicyclohexylcarbodiimide (1.14 g, 0.0055 mol) was added and the mixture was stirred at rt for about 24 h. The reaction process was controlled by TLC. After completion the precipitated dicyclohexylurea was separated, the solvent evaporated and the crude material purified by recrystallization.

General procedure 2 (GP2)
Condensation of anilines with benzaldehydes giving Schiff bases
To a slurry of the substituted benzaldehyde (0.005 mol) and the corresponding aniline (0.005 mol) in 30 mL ethanol, a catalytic amount of acetic acid was added and the mixture heated under reflux for about 10 min. Stirring was continued for 24 h at rt. The precipitate was filtered off and the crude compound was purified by recrystallization. For the preparation of the final products **OH 5** and **OH 6** only 0.0025 mol of the substituted diamine was used.

General procedure 3 (GP3)
Acylation of 2,4-dihydroxybenzaldehyde using acid chlorides
To a slurry of the substituted benzoic acid (0.01 mol) in 50 mL of dry dichloromethane and one drop of pyridine, oxalyl chloride (0.04 mol as 2 M solution in dichloromethane) was added and the mixture refluxed for 2 h. The solvent was evaporated, 50 mL dichloromethane was added again to remove the residue of oxalyl chloride under vacuum together with the solvent. The crude acid chloride was dissolved in 30 mL of dry toluene. After addition of 2,4-dihydroxybenzaldehyde (0.005 mol) and DMAP (catalytic amount) 0.011 mol triethylamine was slowly added dropwise. Stirring was continued for 6 h at 65 °C and afterwards for 10 h at rt. After cooling, the reaction mixture was filtered to remove the triethylamine hydrochloride, and the solvent evaporated in vacuum. The crude compound was purified by recrystallization.

2. Intermediates

Intermediates sketched in Scheme 2

4-Formylphenyl 3-chloro-4-n-dodecyloxybenzoate (**1c**)
Following GP1 from 3-chloro-4-*n*-dodecyloxybenzoic acid [1] and 4-hydroxybenzaldehyde.
Yield: 78%, Cr 103 [68.7] I (ethanol); $C_{26}H_{33}O_4Cl$, M_n = 445.00 g/mol.
^{1}H NMR (400 MHz, $CDCl_3$): δ 0.87 (t, ^3J = 6.9 Hz, 3H, CH_3), 1.23-1.54 (m, 18H, CH_2), 1.87 (m, 2H, ArOCH$_2$*CH$_2$*), 4.11 (t, ^3J = 6.5 Hz, 2H, ArO*CH$_2$*CH$_2$), 6.98 (d, ^3J = 8.7 Hz, 1H, Ar-H), 7.38 (d, ^3J = 8.5 Hz, 2H, Ar-H), 7.95 (d, ^3J = 8.7 Hz, 2H, Ar-H), 8.05 (d, ^3J = 8.6 Hz, 1H, Ar-H), 8.19 (s, 1H, Ar-H), 10.00 (s, 1H, CHO). **^{13}C NMR** (100.6 MHz, $CDCl_3$): δ 14.20, 22.77, 25.98, 28.98, 29.36, 29.42, 29.59, 29.64, 29.71, 29.72, 30.95, 31.99, 69.56, 112.24, 112.49, 122.40, 123.20, 130.53, 131.14, 132.17, 134.01, 155.51, 159.10, 163.14, 190.62.

4-Formylphenyl 3-bromo-4-n-dodecyloxybenzoate (**1d**)
From 3-bromo-4-*n*-dodecyloxybenzoic acid [1] and 4-formylphenol following GP1. Yield: 77%, Cr 108 [68.8] I (Ethanol); $C_{26}H_{33}O_4Br$, M_n = 489.45 g/mol.
^{1}H NMR (400 MHz, $CDCl_3$): δ 0.87 (t, ^3J = 6.9 Hz, 3H, CH_3), 1.26-1.55 (m, 18H, CH_2), 1.87 (m, 2H, ArOCH$_2$*CH$_2$*), 4.11 (t, ^3J = 6.5 Hz, 2H, ArO*CH$_2$*CH$_2$), 6.94 (d, ^3J = 8.7 Hz, 1H, Ar-H), 7.38 (d, ^3J = 8.5 Hz, 2H, Ar-H), 7.95 (d, ^3J = 8.7 Hz, 2H, Ar-H), 8.09 (d, ^3J = 8.7 Hz, 1H, Ar-H), 8.37 (s, 1H, Ar-H), 10.01 (s, 1H, CHO). **^{13}C NMR** (100.6 MHz, $CDCl_3$): δ 14.20, 22.78, 26.00, 28.97, 29.35, 29.43, 29.60, 29.64, 29.73, 32.00, 69.63, 112.03, 112.21, 121.94, 122.41, 131.14, 131.27, 134.01, 135.33, 155.51, 159.93, 163.01, 190.63.

4-(3-Chloro-4-n-dodecyloxybenzoyloxy)benzoic acid (**2c**)

4-Formylphenyl 3-chloro-4-*n*-dodecyloxybenzoate (**1c**) (0.0153 mol/6.8 g) was solved in 80 mL acetic acid at 40 °C and H_2O_2 (30%, 20 ml) was added under stirring within 2 h. The stirred mixture was heated at 75 °C for about 20 h, with the process controlled by TLC. After cooling of the slurry, the precipitate was separated and purified by crystallization from acetic acid.

Yield: 5.17 g (73%), Cr 152 [16.0] N 206 [11.0] I; $C_{26}H_{33}O_5Cl$, M_n = 460.97 g/mol.

^{1}**H NMR** (400 MHz, $CDCl_3$): δ 0.87 (m, 3H, CH_3), 1.25-1.48 (m, 18H, CH_2), 1.86 (m, 2H, $ArOCH_2\mathit{CH_2}$), 4.11 (t, ^3J = 6.4 Hz, 2H, $ArO\mathit{CH_2}CH_2$), 6.98 (d, ^3J = 8.5 Hz, 1H, Ar-H), 7.32 (d, ^3J = 8.5 Hz, 2H, Ar-H), 8.05 (d, ^3J = 8.3 Hz, 1H, Ar-H), 8.18 (m, 3H, Ar-H).

^{13}**C NMR** (125.7 MHz, $CDCl_3$): δ 14.14, 22.72, 25.93, 28.94, 29.32, 29.37, 29.55, 29.59, 29.67, 31.95, 69.54, 112.27, 121.67, 121.87, 123.23, 126.70, 130.58, 131.88, 132.24, 155.22, 159.13, 163.31.

4-(3-Bromo-4-n-dodecyloxybenzoyloxy)benzoic acid (**2d**)

4-Formylphenyl 3-bromo-4-*n*-dodecyloxybenzoate (**1d**) (0.0151 mol/7.4 g) was dissolved in 80 mL acetic acid at 40 °C and H_2O_2 (30%, 20 ml) was added under stirring within 2 h. The stirred mixture was heated at 75 °C for about 20 h, with the process controlled by TLC. After cooling of the slurry, the precipitate was separated and purified by crystallization from acetic acid. Yield: 6.02 g (79%), Cr 142 [13.8] N 193 [5.7] I.

^{1}**H NMR** (400 MHz, $CDCl_3$): δ 0.87 (t, ^3J = 6.8 Hz, 3H, CH_3), 1.26-1.53 (m, 18H, CH_2), 1.87 (m, 2H, $ArOCH_2\mathit{CH_2}$), 4.11 (t, ^3J = 6.4 Hz, 2H, $ArO\mathit{CH_2}CH_2$), 6.95 (d, ^3J = 8.7 Hz, 1H, Ar-H), 7.32 (d, ^3J = 8.9 Hz, 2H, Ar-H), 8.10 (d, ^3J = 8.6 Hz, 1H, Ar-H), 8.18 (d, ^3J = 8.9 Hz, 2H, Ar-H), 8.37 (m, 1H, Ar-H). ^{13}**C NMR** (125.7 MHz, $CDCl_3$): δ 14.21, 22.78, 26.00, 28.98, 29.35, 29.43, 29.61, 29.64, 29.72, 32.00, 69.62, 112.02, 112.18, 121.82, 122.07, 126.65, 131.25, 131.82, 135.32, 155.13, 159.87, 163.07, 169.83.

3-Benzyloxyphenyl 4-(3-bromo-4-n-dodecyloxybenzoyloxy)benzoate (**3d-Bn**)

Following GP1 from 4-(3-bromo-4-*n*-dodecyloxybenzoyloxy)benzoic acid (**2d**) and 3-benzyloxyphenol. Yield: 63%, Cr 80 [45.7] I (ethanol).

^{1}**H NMR** (200 MHz, $CDCl_3$): δ 0.87 (t, ^3J = 6.4 Hz, 3H, CH_3), 1.26-1.51 (m, 18H, CH_2), 1.88 (m, 2H, $ArOCH_2\mathit{CH_2}$), 4.11 (t, ^3J = 6.4 Hz, 2H, $ArO\mathit{CH_2}CH_2$), 5.06 (s, 2H, Ar-$O\mathit{CH_2}$Ph), 6.86 (m, 3H, Ar-H), 6.95 (d, ^3J = 8.7 Hz, 1H, Ar-H), 7.35 (m, 8H, Ar-H), 8.12 (m, 1H, Ar-H), 8.26 (d, ^3J = 8.9 Hz, 2H, Ar-H), 8.38 (s, 1H, Ar-H).

3-Hydroxyphenyl 4-(3-bromo-4-n-dodecyloxybenzoyloxy)benzoate (**3d**)

3-Benzyloxy-phenyl 4-(3-bromo-4-*n*-dodecyloxybenzoyloxy)benzoate (**3d-Bn**) (0.64 mmol/0.44 g), was dissolved in ethyl acetate and hydrogenated at normal pressure in the presence of Pd/C (10% Pd, 0.1 g). Yield: 0.28 g (73%), Cr 108 [19.5] I (ethanol).

^{1}**H NMR** (400 MHz, $CDCl_3$): δ 0.87 (t, ^3J = 6.9 Hz, 3H, CH_3), 1.25-1.52 (m, 18H, CH_2), 1.79 (m, 2H, $ArOCH_2\mathit{CH_2}$), 4.11 (t, ^3J = 6.5 Hz, 2H, $ArO\mathit{CH_2}CH_2$), 4.87 (bs, 1H, OH), 6.73 (m, 1H, Ar-H), 6.79 (s, 1H, Ar-H), 6.95 (m, 2H, Ar-H), 7.27 (t, ^3J = 8.4 Hz, 1H, Ar-H), 7.34 (d, ^3J = 8.5 Hz, 2H, Ar-H), 8.17 (m, 1H, Ar-H), 8.25 (d, ^3J = 8.7 Hz, 2H, Ar-H), 8.38 (s, 1H, Ar-H).

Intermediates sketched in Scheme 3

4-Formyl-3-hydroxyphenyl 4-n-dodecyloxy-3-fluorobenzoate (**5b**)
Following GP3 from 4-*n*-dodecyloxy-3-fluorobenzoic acid [2] and 2,4-dihydroxybenzaldehyde, Yield: 92%, Cr 101 [51.1] I (ethanol); $C_{26}H_{33}O_5F$, M_n = 44.52 g/mol.
^{1}H NMR (400 MHz, $CDCl_3$): δ 0.47 (t, ^3J = 6.8 Hz, 3H, CH_3), 0.86-1.28 (m, 18H, CH_2), 1.45 (m, 2H, ArOCH$_2$*CH$_2$*), 3.74 (t, ^3J = 6.4 Hz, 2H, ArO*CH$_2$*CH$_2$), 6.47 (m, 2H, Ar-H), 6.70 (m, 1H, Ar-H), 7.31 (s, 1H, Ar-H), 7.42 (m, 1H, Ar-H), 7.54 (d, ^3J = 8.7 Hz, 1H, Ar-H), 9.62 (s, 1H, CHO), 10.67 (s, 1H, OH). **^{13}C NMR** (100.6 MHz, $CDCl_3$): δ 13.23, 21.67, 24.02, 24.82, 24.85, 27.98, 28.75, 28.30, 28.50, 28.53, 28.59, 29.97, 30.89, 33.00, 68.54, 109.62, 112.89, 116.53, 118.35, 120.04, 126.68, 132.99, 151.20, 156.28, 156.53, 161.73, 161.96, 195.27.

4-Formyl-3-hydroxyphenyl 3-chloro-4-n-dodecyloxybenzoate (**5c**)
Following GP3 from 3-chloro-4-*n*-dodecyloxybenzoic acid [1] and 2,4-dihydroxybenzaldehyde. Yield: 81%, Cr 79 [31.6] I (ethanol); $C_{26}H_{33}O_5Cl$; M_n = 460.97 g/mol.
^{1}H NMR (400 MHz, $CDCl_3$): δ 0.87 (t, ^3J = 6.4 Hz, 3H, CH_3), 1.25-1.51 (m, 18H, CH_2), 1.87 (m, 2H, ArOCH$_2$*CH$_2$*), 4.11 (t, ^3J = 6.4 Hz, 2H, ArO*CH$_2$*CH$_2$), 6.86 (s, 1H, Ar-H), 6.95 (m, 2H, Ar-H), 7.60 (d, ^3J = 8.3 Hz, 1H, Ar-H), 8.03 (d, ^3J = 8.7 Hz, 1H, Ar-H), 8.17 (s, 1H, Ar-H), 9.87 (s, 1H, CHO), 11.23 (s, 1H, OH).

4-Formyl-3-hydroxyphenyl 3-bromo-4-n-dodecyloxybenzoate (**5d**)
Following GP3 from 4-*n*-dodecyloxy-3-bromobenzoic acid [1] and 2,4-dihydroxybenzaldehyde. Yield: 91%; Cr 73 [36.5] I (ethanol); $C_{26}H_{33}O_5Br$; M_n = 505.43 g/mol.
^{1}H NMR (400 MHz, $CDCl_3$): δ 0.86 (t, ^3J = 6.8 Hz, 3H, CH_3), 1.25-1.70 (m, 18H, CH_2), 1.88 (m, 2H, ArOCH$_2$*CH$_2$*), 4.10 (t, ^3J = 6.4 Hz, 2H, ArO*CH$_2$*CH$_2$), 6.86 (d, ^3J = 8.9 Hz, 1H, Ar-H), 6.89 (s, 1H, Ar-H), 6.93 (d, ^3J = 8.7 Hz, 1H, Ar-H), 7.59 (d, ^3J = 8.3 Hz, 1H, Ar-H), 8.07 (d, ^3J = 8.6 Hz, 1H, Ar-H), 8.34 (s, 1H, Ar-H), 9.87 (s, 1H, CHO), 11.22 (s, 1H, OH). **^{13}C NMR** (100.6 MHz, $CDCl_3$): δ 14.26, 22.84, 25.08, 25.78, 26.05, 29.02, 29.40, 29.48, 29.66, 29.70, 29.79, 32.06, 34.08, 69.69, 110.85, 112.07, 112.26, 113,98, 118.73, 121.89, 131.56, 134.90, 135.40, 157.52, 160.01, 162.72, 163.12, 195.27.

3-Aminophenyl 4-(3-chloro-4-n-dodecyloxy-benzoyloxy)benzoate (**6b**)
3-Nitrophenyl 4-(3-chloro-4-n-dodecyloxy-benzoyloxy)benzoate (**6b-NO₂**)

Following GP1 from 4-(3-chloro-4-*n*-dodecyloxybenzoyloxy)benzoic acid (**2c**) and 3-nitrophenol. Yield: 72%; Cr 95 [35.4] SmA 97 [3.2] I (DMF/ethanol); $C_{32}H_{36}O_7NCl$; M_n = 582.07 g/mol.
^{1}H NMR (400 MHz, $CDCl_3$): δ 0.87 (t, ^3J = 6.8 Hz, 3H, CH_3), 1.26-1.49 (m, 18H, CH_2), 1.80 (m, 2H, $ArOCH_2\mathit{CH_2}$), 4.12 (t, ^3J = 6.5 Hz, 2H, $ArO\mathit{CH_2}CH_2$), 6.99 (d, ^3J = 8.7 Hz, 1H, Ar-H), 7.38 (d, ^3J = 8.9 Hz, 2H, Ar-H), 7.60 (m, 2H, Ar-H), 8.07 (m, 1H, Ar-H), 8.15 (m, 2H, Ar-H), 8.21 (s, 1H, Ar-H), 8.27 (d, ^3J = 8.9 Hz, 2H, Ar-H).
3-Aminophenyl 4-(3-chloro-4-n-dodecyloxy-benzoyloxy)benzoate (**6b**)
3-Nitrophenyl 4-(3-chloro-4-*n*-dodecyloxy-benzoyloxy)benzoate (**6b-NO₂**) was reduced with hydrogen by using Pd/C (10% Pd) in ethyl acetate. Yield: 81% ; Cr 94 [28.9] (SmC 77 [6.6]) I (ethanol); $C_{32}H_{38}O_5NCl$; M_n = 552.08 g/mol.
^{1}H NMR (400 MHz, $CDCl_3$): δ 0.87 (t, ^3J = 6.8 Hz, 3H, CH_3), 1.26-1.54 (m, 18H, CH_2), 1.87 (m, 2H, $ArOCH_2\mathit{CH_2}$), 4.12 (t, ^3J = 6.5 Hz, 2H, $ArO\mathit{CH_2}CH_2$), 6.57 (m, 3H, Ar-H), 6.99 (d, ^3J = 8.7 Hz, 1H, Ar-H), 7.18 (t, ^3J = 8.1 Hz, 1H, Ar-H), 7.33 (d, ^3J = 8.7 Hz, 2H, Ar-H), 8.06 (m, 1H, Ar-H), 8.21 (s, 1H, Ar-H), 8.25 (d, ^3J = 8.7 Hz, 2H, Ar-H).

3-Amino-2-methylphenyl 4-(4-n-dodecyloxybenzoyloxy)benzoate (**6c**)
2-Methyl-3-nitrophenyl 4-(4-n-dodecyloxybenzoyloxy)benzoate (**6c -NO₂**)
Following GP1 from 4-(4-*n*-dodecyloxybenzoyloxy)benzoic acid (**2a**) and 2-methyl-3-nitro-phenol. Yield: 65%, Cr 103 [47.1] SmA 115 [3.1] I (DMF/ ethanol); $C_{33}H_{39}O_7N$; M_n = 561.65 g/mol.
^{1}H NMR (400 MHz, $CDCl_3$): δ 0.87 (t, ^3J = 6.8 Hz, 3H, CH_3), 1.25-1.51 (m, 18H, CH_2), 1.80 (m, 2H, $ArOCH_2\mathit{CH_2}$), 2.41 (s, 1H, Ar-CH_3), 4.04 (t, ^3J = 6.5 Hz, 2H, $ArO\mathit{CH_2}CH_2$), 6.97 (d, ^3J = 8.9 Hz, 2H, Ar-H), 7.38 (d, ^3J = 8.9 Hz, 2H, Ar-H), 7.40 (m, 2H, Ar-H), 7.83 (m, 1H, Ar-H), 8.14 (d, ^3J = 9.1 Hz, 2H, Ar-H), 8.28 (d, ^3J = 8.9 Hz, 2H, Ar-H).

3-Amino-2-methylphenyl 4-(4-n-dodecyloxybenzoyloxy)benzoate (**6c**)
2-Methyl-3-nitrophenyl 4-(4-*n*-dodecyloxybenzoyloxy)benzoate (**6c-NO₂**) was reduced with hydrogen by using Pd/C (10% Pd) in ethyl acetate. Yield: 94%; Cr 121 [36.6] I (ethanol); $C_{33}H_{41}O_5N$; M_n = 531.67 g/mol.
^{1}H NMR (400 MHz, $CDCl_3$): δ 0.87 (t, ^3J = 6.8 Hz, 3H, CH_3), 1.23-1.50 (m, 18H, CH_2), 1.81 (m, 2H, $ArOCH_2\mathit{CH_2}$), 2.15 (s, 1H, Ar-CH_3), 4.04 (t, ^3J = 6.6 Hz, 2H, $ArO\mathit{CH_2}CH_2$), 6.62 (m, 2H, Ar-H), 6.97 (d, ^3J = 9.1 Hz, 2H, Ar-H), 7.06 (t, ^3J = 8.1 Hz, 1H, Ar-H), 7.35 (d, ^3J = 8.7 Hz, 2H, Ar-H), 8.14 (d, ^3J = 9.1 Hz, 2H, Ar-H), 8.28 (d, ^3J = 8.9 Hz, 2H, Ar-H).

3-Amino-2-methylphenyl 4-(3-chloro-4-n-dodecyloxybenzoyloxy)benzoate (**6d**)
2-Methyl-3-nitrophenyl 4-(3-chloro-4-n-dodecyloxybenzoyloxy)benzoate(**6d-NO₂**)
Following GP1 from 4-(3-chloro-4-*n*-dodecyloxybenzoyloxy)benzoic acid (**2c**) and 2-methyl-3-nitrophenol. Yield: 60%; , Cr 95 [43.7] I (DMF/ethanol). $C_{33}H_{38}O_7NCl$; M_n = 596.09 g/mol.

^{1}H NMR (400 MHz, $CDCl_3$): δ 0.87 (t, ^3J = 6.9 Hz, 3H, CH_3), 1.26-1.51 (m, 18H, CH_2), 1.88 (m, 2H, ArOCH$_2$*CH$_2$*), 2.41 (s, 1H, Ar-CH_3), 4.12 (t, ^3J = 6.5 Hz, 2H, ArO*CH$_2$*CH_2), 6.99 (d, ^3J = 8.7 Hz, 1H, Ar-H), 7.38 (d, ^3J = 8.7 Hz, 2H, Ar-H), 7.39 (m, 2H, Ar-H), 7.83 (m, 1H, Ar-H), 8.07 (m, 1H, Ar-H), 8.21 (s, 1H, Ar-H), 8.27 (d, ^3J = 8.7 Hz, 2H, Ar-H).

3-Amino-2-methylphenyl 4-(3-chloro-4-n-dodecyloxy-benzoyloxy)benzoate (**6d**)
2-Methyl-3-nitrophenyl 4-(3-chloro-4-*n*-dodecyloxybenzoyloxy)benzoate (**6d-NO$_2$**) was reduced with hydrogen by using Pd/C (10% Pd) in ethyl acetate. Yield: 94%; Cr 121 [40.4] I (ethanol); $C_{33}H_{40}O_5NCl$; M_n = 566.11 g/mol.
^{1}H NMR (400 MHz, $CDCl_3$): δ 0.87 (t, ^3J = 6.8 Hz, 3H, CH_3), 1.23-1.54 (m, 18H, CH_2), 1.88 (m, 2H, ArOCH$_2$*CH$_2$*), 2.01 (s, 1H, Ar-CH_3), 3.93 (bs, 2H, NH_2), 4.12 (t, ^3J = 6.5 Hz, 2H, ArO*CH$_2$*CH_2), 6.61 (m, 2H, Ar-H), 6.99 (d, ^3J = 8.7 Hz, 1H, Ar-H), 7.06 (t, ^3J = 7.5 Hz, 1H, Ar-H), 7.35 (d, ^3J = 8.9 Hz, 2H, Ar-H), 8.07 (m, 1H, Ar-H), 8.21 (s, 1H, Ar-H), 8.28 (d, ^3J = 8.7 Hz, 2H, Ar-H).

3-Hydroxy-[4-(3-hydroxyphenyliminomethyl)]phenyl 4-n-dodecyloxybenzoate (**7a**)
Following GP2 from 4-formyl-3-hydroxyphenyl 4-*n*-dodecyloxybenzoate (**5a**) [3] and 3-amino-phenol. Yield: 66%, slightly yellow solid, Cr 146 [30.3] (N 145 [1.6]) I (ethanol); $C_{32}H_{39}O_5N$; M_n = 517.64 g/mol.
^{1}H NMR (400 MHz, $CDCl_3$): δ 0.87 (t, ^3J = 6.8 Hz, 3H, CH_3), 1.22-1.50 (m, 18H, CH_2), 1.80 (m, 2H, ArOCH$_2$*CH$_2$*), 4.03 (t, ^3J = 6.5 Hz, 2H, ArO*CH$_2$*CH_2), 5.24 (bs, 1H, OH), 6.80 (m, 5H, Ar-H), 6.96 (d, ^3J = 8.9 Hz, 2H, Ar-H), 7.25 (m, 1H, Ar-H), 7.38 (d, ^3J = 8.5 Hz, 1H, Ar-H), 8.12 (d, ^3J = 8.9 Hz, 2H, Ar-H), 8.57 (s, 1H, CH=N). **^{13}C NMR** (100.6 MHz, $CDCl_3$): δ 14.14, 22.72, 26.02, 29.13, 29.37, 29.58, 29.61, 29.66, 29.68, 31.94, 68.41, 108.30, 109.85, 110.70, 113.12, 113.43, 114.03, 114.38, 116.93, 121.14, 130.39, 132.37, 133.21, 149.43, 155.02, 156.55, 161.83, 162.85, 163.72, 164.42.

3-Hydroxy-[4-(3-hydroxy-4-methylphenyliminomethyl)phenyl] 4-n-dodecyloxybenzoate (**7b**)
Following GP2 from 4-formyl-3-hydroxyphenyl 4-*n*-dodecyloxybenzoate (**5a**) [3] and 3-amino-6-methylphenol. Yield: 86%, slightly yellow solid, Cr 190 [40.0] N 196 [1.1] I (ethanol); $C_{33}H_{41}O_5N$, M_n = 531.67 g/mol.
^{1}H NMR (400 MHz, $CDCl_3$, DMSO-d_6): δ 0.79 (t, ^3J = 6.8 Hz, 3H, CH_3), 1.18-1.43 (m, 18H, CH_2), 1.73 (m, 2H, ArOCH$_2$*CH$_2$*), 2.13 (s, 3H, Ar-CH_3), 3.97 (t, ^3J = 6.5 Hz, 2H, ArO*CH$_2$*CH_2), 6.63 (m, 1H, Ar-H), 6.73 (m, 2H, Ar-H), 6.90 (d, ^3J = 8.7 Hz, 2H, Ar-H), 7.01 (m, 1H, Ar-H), 7.39 (d, ^3J = 8.1 Hz, 1H, Ar-H), 7.56 (s, 1H, Ar-H), 8.01 (d, ^3J = 8.7 Hz, 2H, Ar-H), 8.58 (s, 1H, CH=N), 8.96 (s, 1H, OH). **^{13}C NMR** (100.6 MHz, $CDCl_3$, DMSO-d_6): δ 19.06, 20.84, 27.47, 30.78, 33.89, 34.10, 34.12, 34.33, 34.34, 34.39, 34.41, 36.68, 73.18, 112.63, 115.10, 116.36, 117.66, 119.24, 121.90, 125.76, 128.68, 135.98, 136.86, 137.87, 151.18, 159.23, 160.94, 167.13, 168.37, 168.82.

Intermediates sketched in Schemes 4 and 5

4-(4-n-Dodecyloxy-2-hydroxybenzylideneamino)phenol (**8**)
Following GP2 from 4-*n*-dodecyloxy-2-hydroxybenzaldehyde [4] and 4-aminophenol. Yield: 63%, slightly yellow solid, Cr 130 [27.6] (SmA 126 [5.0]) I (ethanol); $C_{25}H_{35}O_3N$; M_n = 397.54 g/mol.
^{1}H NMR (400 MHz, $CDCl_3$): δ 0.87 (t, ^3J = 6.8 Hz, 3H, CH_3), 1.25-1.43 (m, 18H, CH_2), 1.77 (m, 2H, ArOCH$_2$*CH$_2$*), 3.97 (t, ^3J = 6.2 Hz, 2H, ArO*CH$_2$*CH$_2$), 6.45 (m, 1H, Ar-H), 6.49 (s, 1H, Ar-H), 6.86 (d, ^3J = 8.7 Hz, 2H, Ar-H), 7.15 (d, ^3J = 8.7 Hz, 2H, Ar-H), 7.22 (d, ^3J = 8.9 Hz, 1H, Ar-H), 8.45 (s, 1H, CH=N).

4-Formyl-3-hydroxyphenyl 4-n-dodecyloxycinnamate (**9**)
Following GP3 from 4-*n*-dodecyloxycinnamic acid and 2,4-dihydroxybenzaldehyde. Yield: 66%; Cr 93 [48.7] (SmA 90 [1.1]) I (ethanol); $C_{28}H_{36}O_5$; M_n = 452.57 g/mol.
^{1}H NMR (400 MHz, $CDCl_3$): δ 0.87 (t, ^3J = 6.9 Hz, 3H, CH_3), 1.25-1.48 (m, 18H, CH_2), 1.79 (m, 2H, ArOCH$_2$*CH$_2$*), 3.99 (t, ^3J = 6.1 Hz, 2H, ArO*CH$_2$*CH$_2$), 6.43 (d, ^3J = 16.0 Hz, 1H, Ar-CH=*CH*-COOAr), 6.82 (s, 1H, Ar-H), 6.86 (m, 1H, Ar-H), 6.91 (d, ^3J = 8.7 Hz, 2H, Ar-H), 7.51 (d, ^3J = 8.9 Hz, 2H, Ar-H), 7.57 (m, 1H, Ar-H), 7.82 (d, ^3J = 15.8 Hz, 1H, Ar-*CH*=CH-COOAr), 9.95 (s, 1H, CHO), 11.21 (s, 1H, OH). **^{13}C NMR** (100.6 MHz, $CDCl_3$): δ 14.21, 22.77, 26.08, 29.22, 29.42, 29.43, 29.63, 29.66, 29.71, 29.73, 31.99, 68.29, 110.64, 113.51, 113.95, 114.95, 118.47, 126.33, 130.11, 134.76, 147.34, 157.63, 161.54, 163.03, 164.48, 195.19.

Final compounds OH 1–6 and H 1–6
The compounds are reported as listed in Tables 1–6.

Compounds H 1 and OH 1 (Table 1)

3-[4-(4-n-Dodecyloxybenzylideneamino)benzoyloxy]phenyl 4-(4-n-dodecyloxy-benzoyloxy)benzoate (**H 1a**)

Following GP1 from 4-(4-*n*-dodecyloxybenzylideneamino)benzoic acid [5] and 3-hydroxy-phenyl 4-(4-*n*-dodecyloxybenzoyloxy)benzoate (**3a**) [6]; yield: 89%, colourless solid from DMF/ethanol. $C_{58}H_{71}O_8N$; M_n = 910.16 g/mol; EA: calculated: C 76.53, H 7.86, N 1.54; found: C 76.22, H 7.90, N 1.30.
^{1}H NMR (400 MHz, $CDCl_3$): δ 0.87 (t, ^3J = 6.95 Hz, 6H, CH_3), 1.23-1.50 (m, 36H, CH_2), 1.80 (m, 4H, ArOCH$_2$*CH$_2$*), 4.03 (m, 4H, ArO*CH$_2$*CH$_2$), 6.97 (d, ^3J = 8.9 Hz, 4H, Ar-H), 7.18 (m, 3H, Ar-H), 7.24 (m, 2H, Ar-H), 7.36 (d, ^3J = 8.9 Hz, 2H, Ar-H), 7.47 (t, ^3J = 8.1 Hz, 1H, Ar-H), 7.85 (d, ^3J = 8.7 Hz, 2H, Ar-H), 8.13 (d, ^3J = 8.9 Hz, 4H, Ar-H), 8.20 (d, ^3J = 8.9 Hz, 2H, Ar-H), 8.26 (d, ^3J = 8.9 Hz, 2H, Ar-H), 8.37 (s, 1H, CH=N); **^{13}C NMR** (100.6 MHz, $CDCl_3$): δ 14.25, 22.83, 26.13, 26.16, 29.24, 29.31, 29.48, 29.69, 29.72, 29.77, 29.79, 32.05, 68.41, 68.51, 114,45, 114.77, 114.85, 115.82, 119.08, 119.30, 120.94, 121.01, 122.09, 125.99, 126.68, 129.76, 130.93, 131.51, 131.79, 132.38, 151.39, 151.57, 155.44, 161.02, 162.39, 163.77, 163.98, 164.18, 164.46.

3-[4-(4-n-Dodecyloxy-2-hydroxybenzylideneamino)benzoyloxy]phenyl 4-(4-n-dodecyloxybenzoyloxy)benzoate (**OH 1**)

Following GP1 from 4-(4-*n*-dodecyloxy-2-hydroxybenzylideneamino)benzoic acid (**4**) [4] and 3-hydroxyphenyl 4-(4-*n*-dodecyloxy-benzoyloxy)benzoate (**3a**) [6]; yield: 65%, light yellow solid (DMF/ethanol), $C_{58}H_{71}O_9N$; M_n = 926.16 g/mol; EA: calculated: C 75.21, H 7.23, N 1.51; found: C 74.72, H 7.74, N 1.28.
^{1}H NMR (400 MHz, $CDCl_3$): δ 0.87 (t, ^3J = 6.85 Hz, 6H, CH_3), 1.26-1.50 (m, 36H, CH_2), 1.80 (m, 4H, ArOCH$_2$*CH$_2$*), 4.02 (m, 4H, ArO*CH$_2$*CH$_2$), 6.51 (m, 2H, Ar-H), 6.97 (d, ^3J = 8.9 Hz, 2H, Ar-H), 7.19 (m, 3H, Ar-H), 7.32 (m, 2H, Ar-H), 7.36 (d, ^3J = 8.9 Hz, 4H, Ar-H), 7.48 (m, 1H, Ar-H), 8.13 (d, ^3J = 8.9 Hz, 2H, Ar-H), 8.23 (d, ^3J = 8.7 Hz, 2H, Ar-H), 8.26 (d, ^3J = 8.9 Hz, 2H, Ar-H), 8.55 (s, 1H, CH=N).

3-[4-(4-n-Dodecyloxy-2-hydroxybenzylideneamino)benzoyloxy]phenyl 4-(3-chloro-4-n-dodecyloxybenzoyloxy)benzoate (**OH 1b**)

Following GP 1 from 4-(4-*n*-dodecyloxy-2-hydroxybenzylideneamino)benzoic acid (**4**) [4] and 3-hydroxyphenyl 4-(3-chloro-4-*n*-dodecyloxybenzoyloxy)benzoate (**3c**) [7]; yield: 65%, yellow solid (DMF/ethanol); $C_{58}H_{70}O_9NCl$; M_n = 960.65 g/mol; EA: calc: C 72.51, H 7.34, N 1.46, Cl 3.69; found: C 72.22, H 7.44, N 1.27, Cl 3.57.
^{1}H NMR (400 MHz, $CDCl_3$): δ 0.87 (t, ^3J = 6.85 Hz, 6H, CH_3), 1.26-1.52 (m, 36H, CH_2), 1.77-1.89 (m, 4H, ArOCH$_2$*CH$_2$*), 3.99-4.13 (m, 4H, ArO*CH$_2$*CH$_2$), 6.49-6.54 (m, 2H, Ar-H), 6.99 (d, ^3J = 8.2 Hz, 1H, Ar-H), 7.18 (m, 3H, Ar-H), 7.29 (d, ^3J = 8.7 Hz, 1H, Ar-H), 7.35 (d, ^3J = 8.8 Hz, 4H, Ar-H), 7.48 (t, ^3J = 7.8 Hz, 1H, Ar-H), 8.06 (d, ^3J = 8.6 Hz, 1H, Ar-H), 8.21 (m, 1H, Ar-H), 8.23 (d, ^3J = 8.7 Hz, 2H, Ar-H), 8.26 (d, ^3J = 8.9 Hz, 2H, Ar-H), 8.55 (s, 1H, CH=N).

3-[4-(4-n-Dodecyloxy-2-hydroxybenzylideneamino)benzoyloxy]phenyl 4-(3-bromo-4-n-dodecyloxybenzoyloxy)benzoate (**OH 1c**)

Following GP 1 from 4-(4-*n*-dodecyloxy-2-hydroxybenzylideneamino)benzoic acid (**4**) [4] and 3-hydroxyphenyl 4-(3-bromo-4-*n*-dodecyloxybenzoyloxy)benzoate (**3d**); yield: 72%, yellow solid (DMF/ethanol); $C_{58}H_{70}O_9NBr$, M_n = 1005.05 g/mol, EA: calculated: C 69.31, H 7.02, N 1.39, Br 7.95; found: C 69.17, H 7.11, N 1.29, Br 7.24.
^{1}H NMR (400 MHz, $CDCl_3$): δ 0.87 (t, ^3J = 6.85 Hz, 6H, CH_3), 1.26-1.55 (m, 36H, CH_2), 1.75-1.91 (m, 4H, ArOCH$_2$*CH$_2$*), 3.99-4.13 (m, 4H, ArO*CH$_2$*CH$_2$), 6.49-6.52 (m, 2H, Ar-H), 6.95 (d, ^3J = 8.7 Hz, 1H, Ar-H), 7.15-7.20 (m, 3H, Ar-H), 7.28 (d, ^3J = 8.3 Hz, 1H, Ar-H), 7.33 (d, ^3J = 8.5 Hz, 2H, Ar-H), 7.36 (d, ^3J = 8.7 Hz, 2H, Ar-H), 7.48 (t, ^3J = 8.1 Hz, 1H, Ar-H), 8.11 (m, 1H, Ar-H), 8.23 (d, ^3J = 8.7 Hz, 2H, Ar-H), 8.26 (d, ^3J = 8.9 Hz, 2H, Ar-H), 8.38 (s, 1H, Ar-H), 8.55 (s, 1H, CH=N).

Compounds H 2 and OH 2 (Table 2)

3-[4-(4-n-Dodecyloxybenzoyloxy)benzylideneamino]phenyl 4-(4-n-dodecyloxybenzoyloxy)benzoate (**H 2a**)

Following GP2 from 4-formylphenyl 4-*n*-dodecyloxybenzoate [8] and 3-aminophenyl 4-(4-*n*-dodecyloxybenzoyloxy)benzoate (**6a**) [9]; yield: 89%, light yellow solid (DMF/ethanol); $C_{58}H_{71}O_8N$; M_n = 910.16 g/mol; EA: calculated: C 76.53, H 7.86, N 1.54; found: C 76.29, H 7.88, N 1.34.
^{1}H NMR (400 MHz, $CDCl_3$): δ 0.87 (t, ^3J = 6.6 Hz, 6H, CH_3), 1.21-1.48 (m, 36H, CH_2), 1.81 (m, 4H, ArOCH_2*CH_2*), 4.04 (m, 4H, ArO*CH_2*CH_2), 6.53 (m, 2H, Ar-H), 6.96 (d, ^3J = 8.9 Hz, 4H, Ar-H), 7.14 (m, 3H, Ar-H), 7.35 (m, 3H, Ar-H), 7.95 (m, 2H, Ar-H), 8.13 (d, ^3J = 8.9 Hz, 4H, Ar-H), 8.25 (m, 2H, Ar-H), 8.48 (s, 1H, CH=N).

3-[4-(4-n-Dodecyloxybenzoyloxy)-2-hydroxybenzylideneamino]phenyl 4-(4-n-dodecyloxybenzoyloxy)benzoate (**OH 2a**)

Following GP2 from 4-formyl-3-hydroxyphenyl 4-*n*-dodecyloxybenzoate (**5a**) [10] and 3-aminophenyl 4-(4-*n*-dodecyloxybenzoyloxy)benzoate (**6a**) [9]; yield: 80%, light-yellow solid, recrystallized from DMF/ethanol; $C_{58}H_{71}O_9N$, M_n = 926.16 g/mol; EA: calculated: C 75.21, H 7.23, N 1.51; found: C 74.77, H 7.77, N 1.26.
^{1}H NMR (400 MHz, $CDCl_3$): δ 0.87 (t, ^3J = 6.8 Hz, 6H, CH_3), 1.26-1.48 (m, 36H, CH_2), 1.81 (m, 4H, ArOCH_2*CH_2*), 4.03 (m, 4H, ArO*CH_2*CH_2), 6.83 (m, 2H, Ar-H), 6.97 (m, 4H, Ar-H), 7.18 (m, 3H, Ar-H), 7.37 (d, ^3J = 8.7 Hz, 2H, Ar-H), 7.42 (m, 2H, Ar-H), 8.13 (m, 4H, Ar-H), 8.28 (d, ^3J = 8.7 Hz, 2H, Ar-H), 8.64 (s, 1H, CH=N).

3-[4-(4-n-Dodecyloxybenzoyloxy)-2-hydroxybenzylideneamino]-6-methylphenyl 4-(4-n-dodecyloxybenzoyloxy)benzoate (**OH 2b**)

Following GP1 from 4-(4-*n*-dodecyloxybenzoyloxy)benzoic acid (**2a**) and 3-hydroxy-[4-(3-hydroxy-4-methylphenyliminomethyl)phenyl] 4-*n*-dodecyloxybenzoate (**7b);** yield: 44%, yellow solid, recrystallized from DMF/ethanol; $C_{59}H_{73}O_9N$; M_n = 940.18 g/mol; EA: calculated: C 75.37, H 7.83, N 1.49; found: C 75.17, H 7.77, N 1.33.
^{1}H NMR (400 MHz, $CDCl_3$): δ 0.87 (t, ^3J = 6.8 Hz, 6H, CH_3), 1.25-1.48 (m, 36H, CH_2), 1.77-1.83 (m, 4H, ArOCH_2*CH_2*), 2.25 (s, 3H, CH_3), 4.01-4.06 (m, 4H, ArO*CH_2*CH_2), 6.81 (d, ^3J = 8.4 Hz, 1H, Ar-H), 6.87 (s, 1H, Ar-H), 6.96 (m, 4H, Ar-H), 6.14 (m, 2H, Ar-H), 7.32 (d, ^3J = 8.1 Hz, 1H, Ar-H), 7.37 (d, ^3J = 8.7 Hz, 2H, Ar-H), 7.40 (d, ^3J = 8.5 Hz, 1H, Ar-H), 8.11 (d, ^3J = 8.9 Hz, 2H, Ar-H), 8.14 (d, ^3J = 8.9 Hz, 2H, Ar-H), 8.29 (d, ^3J = 8.7 Hz, 2H, Ar-H), 8.63 (s, 1H, CH=N). **^{13}C NMR** (100.6 MHz, $CDCl_3$): δ 14.26, 16.20, 22.84, 26.13, 29.25, 29.50, 29.69, 29.72, 29.77, 29.79, 32.06, 68.51, 110.64, 113.16, 114.38, 114.46, 114.64, 117.01, 120.96, 122.18, 129.14, 131.81, 132.33, 132.39, 133.18, 149.70, 151.60, 155.10, 156.10, 160.10, 161.72, 163.79, 163.94, 164.20.

3-[4-(4-n-Dodecyloxybenzoyloxy)-2-hydroxybenzylideneamino]-2-methylphenyl 4-(4-n-dodecyloxybenzoyloxy)benzoate (**OH 2c**)

Following GP2 from 4-formyl-3-hydroxyphenyl 4-*n*-dodecyloxybenzoate (**5a**) [10] and 3-amino-2-methylphenyl 4-(4-*n*-dodecyloxybenzoyloxy)benzoate (**6c**)**;** yield: 66%, yellow solid, (DMF/ethanol). $C_{59}H_{73}O_9N$; M_n = 940.18 g/mol; EA: calculated: C 75.37, H 7.83, N 1.49; found: C 74.98, H 7.80, N 1.34.
^{1}H NMR (400 MHz, $CDCl_3$): δ 0.87 (t, ^3J = 6.8 Hz, 6H, CH_3), 1.26-1.48 (m, 36H, CH_2), 1.79-1.83 (m, 4H, $ArOCH_2\mathit{CH_2}$), 2.26 (s, 3H, CH_3), 4.02-4.06 (m, 4H, $ArO\mathit{CH_2}CH_2$), 6.84 (m, 1H, Ar-H), 6.89 (s, 1H, Ar-H), 6.96 (d, ^3J = 8.9 Hz, 2H, Ar-H), 6.98 (d, ^3J = 8.9 Hz, 2H, Ar-H), 7.02 (d, ^3J = 7.5 Hz, 1H, Ar-H), 7.10 (d, ^3J = 7.9 Hz, 1H, Ar-H), 7.31 (t, ^3J = 7.9 Hz, 1H, Ar-H), 7.38 (d, ^3J = 8.9 Hz, 2H, Ar-H), 7.45 (d, ^3J = 8.1 Hz, 1H, Ar-H), 8.13 (m, 4H, Ar-H), 8.30 (d, ^3J = 8.7 Hz, 2H, Ar-H), 8.57 (s, 1H, CH=N). **^{13}C NMR** (100.6 MHz, $CDCl_3$): δ 11.51, 14.27, 22.84, 26.14, 29.26, 29.49, 29.51, 29.70, 29.73, 29.78, 29.80, 32.06, 68.47, 68.51, 110.67, 113.24, 114.39, 114.46, 116.03, 117.14, 120.50, 120.99, 121.23, 122.18, 125.02, 126.61, 127.08, 131.83, 132.36, 132.40, 133.26, 149.15, 150.03, 155.10, 155.47, 162.56, 163.65, 163.79, 164.07, 164.22.

3-[4-(4-n-Dodecyloxybenzoyloxy)-2-hydroxybenzylideneamino]-2-methylphenyl 4-(3-chloro-4-n-dodecyloxybenzoyloxy)benzoate (**OH 2d**)

Following GP2 from 4-formyl-3-hydroxyphenyl 4-*n*-dodecyloxy-benzoate (**5a**) [10] and 3-amino-2-methylphenyl 4-(3-chloro-4-*n*-dodecyloxybenzoyloxy)benzoate (**6c**); yield: 72%, yellow solid (DMF/ethanol); $C_{59}H_{72}O_9NCl$; M_n = 974.63 g/mol; EA: calculated: C 71.47, H 7.45, N 1.44, Cl 3.64; found: C 71.36, H 7.62, N 1.25, Cl 3.71.
^{1}H NMR (400 MHz, $CDCl_3$): δ 0.87 (t, ^3J = 6.7 Hz, 6H, CH_3), 1.26-1.53 (m, 36H, CH_2), 1.79-1.90 (m, 4H, $ArOCH_2\mathit{CH_2}$), 2.26 (s, 3H, CH_3), 4.02-4.14 (m, 4H, $ArO\mathit{CH_2}CH_2$), 6.84 (m, 1H, Ar-H), 6.89 (s, 1H, Ar-H), 6.96 (d, ^3J = 8.9 Hz, 2H, Ar-H), 6.99 (d, ^3J = 8.9 Hz, 1H, Ar-H), 7.03 (d, ^3J = 7.9 Hz, 1H, Ar-H), 7.10 (d, ^3J = 7.9 Hz, 1H, Ar-H), 7.31 (t, ^3J = 7.9 Hz, 1H, Ar-H), 7.37 (d, ^3J = 8.7 Hz, 2H, Ar-H), 7.45 (d, ^3J = 8.5 Hz, 1H, Ar-H), 8.07 (d, ^3J = 8.7 Hz, 1H, Ar-H), 8.12 (d, ^3J = 8.9 Hz, 2H, Ar-H), 8.21 (s, 1H, Ar-H), 8.31 (d, ^3J = 8.7 Hz, 2H, Ar-H), 8.57 (s, 1H, CH=N).

3-[4-(4-n-Dodecyloxybenzoyloxy)-2-hydroxybenzylideneamino]phenyl 4-(4-n-dodecyloxy-3-fluorobenzoyloxy)benzoate (**OH 2e**)

Following GP1 from 4-(4-*n*-dodecyloxy-3-fluorobenzoyloxy)benzoic acid (**2b**) [2], 3-hydroxy-4-(3-hydroxyphenyliminomethyl)phenyl 4-(4-*n*-dodecyloxybenzoyloxy)benzoate (**7a**); yield: 47%, light-yellow solid (DMF/ethanol); $C_{58}H_{70}O_9NF$; M_n = 944.15 g/mol; EA: calculated: C 73.78, H 7.47, N 1.48; found: C 73.34, H 7.97, N 0.92.
^{1}H NMR (400 MHz, $CDCl_3$): δ 0.87 (t, ^3J = 6.8 Hz, 6H, CH_3), 1.26-1.52 (m, 36H, CH_2), 1.77-1.90 (m, 4H, $ArOCH_2\mathit{CH_2}$), 4.02-4.13 (m, 4H, $ArO\mathit{CH_2}CH_2$), 6.82 (d, ^3J = 8.4 Hz, 1H, Ar-H), 6.88 (s, 1H, Ar-H), 6.96 (d, ^3J = 8.9 Hz, 2H, Ar-H), 7.03 (d, ^3J = 8.4 Hz, 1H, Ar-H), 7.18 (m, 3H, Ar-H), 7.37 (d, ^3J = 8.7 Hz, 2H, Ar-H), 7.42 (d, ^3J = 8.5 Hz, 1H, Ar-H), 7.47 (t,

^3J = 7.9 Hz, 1H, Ar-H), 7.89 (s, 1H, Ar-H), 7.96 (d, ^3J = 8.5 Hz, 1H, Ar-H), 8.12 (d, ^3J = 8.9 Hz, 2H, Ar-H), 8.28 (d, ^3J = 8.9 Hz, 2H, Ar-H), 8.64 (s, 1H, CH=N). **^{13}C NMR** (100.6 MHz, $CDCl_3$): δ 14.25, 22.83, 26.01, 26.13, 29.15, 29.25, 29.47, 29.66, 29.72, 29.76, 32.05, 68.47, 69.66, 110.69, 113.24, 113.61, 114.39, 114.52, 116.94, 117.78, 121.24, 122.01, 126.89, 127.52, 130.20, 131.85, 132.34, 133.32, 149.66, 151.67, 152.29, 155.14, 159.12, 160.10, 162.61, 163.65, 164.16.

3-[4-(4-n-Dodecyloxybenzoyloxy)-2-hydroxybenzylideneamino]phenyl 4-(3-chloro-4-n-dodecyloxybenzoyloxy)benzoate (**OH 2f**)

Following GP2 from 4-formyl-3-hydroxyphenyl 4-*n*-dodecyloxybenzoate (**5a**) [10] and 3-aminophenyl 4-(3-chloro-4-*n*-dodecyloxybenzoyloxy)benzoate (**6b**); yield: 58%, yellow solid (DMF/ethanol); $C_{58}H_{70}O_9NCl$; M_n = 960.60 g/mol; EA: calculated: C 72.51, H 7.35, N 1.46, Cl 3.69; found: C 72.22, H 7.37, N 1.27, Cl 3.64.
^{1}H NMR (400 MHz, $CDCl_3$): δ 0.87 (t, ^3J = 6.8 Hz, 6H, CH_3), 1.26-1.53 (m, 36H, CH_2), 1.77-1.90 (m, 4H, ArOCH$_2$*CH$_2$*), 4.02-4.14 (m, 4H, ArO*CH$_2$*CH$_2$), 6.83 (d, ^3J = 8.4 Hz, 1H, Ar-H), 6.88 (s, 1H, Ar-H), 6.96 (d, ^3J = 8.9 Hz, 2H, Ar-H), 6.99 (d, ^3J = 8.7 Hz, 1H, Ar-H), 7.18 (m, 3H, Ar-H), 7.37 (d, ^3J = 8.7 Hz, 2H, Ar-H), 7.42 (d, ^3J = 8.5 Hz, 1H, Ar-H), 7.47 (t, ^3J = 8.0 Hz, 1H, Ar-H), 8.07 (d, ^3J = 8.7 Hz, 1H, Ar-H), 8.12 (d, ^3J = 8.9 Hz, 2H, Ar-H), 8.21 (s, 1H, Ar-H), 8.28 (d, ^3J = 8.7 Hz, 2H, Ar-H), 8.64 (s, 1H, CH=N)..**^{13}C NMR** (100.6 MHz, $CDCl_3$): δ 14.27, 22.84, 26.05, 26.14, 29.06, 29.26, 29.44, 29.49, 29.51, 29.67, 29.71, 29.72, 29.74, 29.78, 29.80, 32.06, 68.47, 69.62, 110.67, 112.31, 113.24, 114.39, 114.53, 116.95, 119.12, 120.04, 121.23, 121.66, 121.89, 122.02, 123.25, 126.88, 130.19, 130.59, 131.85, 132.23, 132.34, 133.34, 149.62, 151.67, 155.13, 155.19, 159.12, 162.54, 162. 60, 163.25, 163.65, 164.16, 164.17.

3-[4-(4-n-Dodecyloxybenzoyloxy)-2-hydroxybenzylideneamino]phenyl 4-(3-bromo-4-n-dodecyloxybenzoyloxy)benzoate (**OH 2g**)
Following GP1 from 4-(3-bromo-4-*n*-dodecyloxybenzoyloxy)benzoic acid (**2d**) and 3-hydroxy-4-(3-hydroxyphenyliminomethyl)phenyl 4-(4-*n*-dodecyloxybenzoyloxy)benzoate (**7a**); yield: 50%, yellow solid (DMF/ethanol), $C_{58}H_{70}O_9NBr$; M_n = 1005.05 g/mol; EA: calculated: C 69.31, H 7.02, N 1.39, Br 7.95; found: C 69.16, H 7.08, N 1.21, Br 7.96.
^{1}H NMR (400 MHz, $CDCl_3$): δ 0.87 (t, ^3J = 6.8 Hz, 6H, CH_3), 1.26-1.54 (m, 36H, CH_2), 1.79-1.90 (m, 4H, ArOCH$_2$*CH$_2$*), 4.02-4.13 (m, 4H, ArO*CH$_2$*CH$_2$), 6.82 (d, ^3J = 8.3 Hz, 1H, Ar-H), 6.89 (s, 1H, Ar-H), 6.95 (d, ^3J = 8.9 Hz, 2H, Ar-H), 6.96 (d, ^3J = 8.9 Hz, 2H, Ar-H), 7.18 (m, 3H, Ar-H), 7.37 (d, ^3J = 8.7 Hz, 2H, Ar-H), 7.42 (d, ^3J = 8.5 Hz, 1H, Ar-H), 7.47 (t, ^3J = 7.9 Hz, 1H, Ar-H), 8.12 (d, ^3J = 8.9 Hz, 4H, Ar-H), 8.28 (d, ^3J = 8.7 Hz, 2H, Ar-H), 8.38 (s, 1H, Ar-H), 8.64 (s, 1H, CH=N). **^{13}C NMR** (100.6 MHz, $CDCl_3$): δ 14.27, 22.85, 26.08, 26.14, 29.05, 29.26, 29.43, 29.50, 29.68, 29.71, 29.72, 29.74, 29.80, 32.07, 68.47, 69.69, 110.68, 112.10, 112.26, 113.24, 114.40, 114.53, 116.95, 119.13, 120.05, 121.23, 121.90, 122.03, 122.11, 126.88, 130.19, 131.32, 131.85, 132.34, 132.42, 133.34, 135.38, 149.62, 151.67, 155.13, 155.19, 159.95, 162.54, 162. 60, 163.12, 163.65, 164.17.

3-[4-(4-n-Dodecyloxy-3-fluorobenzoyloxy)-2-hydroxybenzylideneamino]phenyl 4-(4-n-dodecyloxybenzoyloxy)benzoate (**OH 2h**)

Following GP2 from 4-formyl-3-hydroxyphenyl 4-*n*-dodecyloxy-3-fluorobenzoate (**5b**) and 3-aminophenyl 4-(4-*n*-dodecyloxybenzoyloxy)benzoate (**6a**) [9]; yield: 57%, light-yellow solid (DMF/ethanol); $C_{58}H_{70}O_9NF$, M_n = 944.15 g/mol; EA: calculated: C 73.78, H 7.47, N 1.48; found: C 73.36, H 7.92, N 0.89.
^{1}H NMR (400 MHz, $CDCl_3$): δ 0.87 (t, ^3J = 6.7 Hz, 6H, CH_3), 1.26-1.48 (m, 36H, CH_2), 1.78-1.87 (m, 4H, ArOCH$_2$*CH$_2$*), 4.03-4.13 (m, 4H, ArO*CH$_2$*CH$_2$), 6.82 (d, ^3J = 8.4 Hz, 1H, Ar-H), 6.88 (s, 1H, Ar-H), 6.97 (d, ^3J = 8.9 Hz, 2H, Ar-H), 7.02 (d, ^3J = 8.3 Hz, 1H, Ar-H), 7.19 (m, 3H, Ar-H), 7.37 (d, ^3J = 8.7 Hz, 2H, Ar-H), 7.43 (d, ^3J = 8.5 Hz, 1H, Ar-H), 7.48 (t, ^3J = 8.1 Hz, 1H, Ar-H), 7.87 (m, 1H, Ar-H), 7.93 (m, 1H, Ar-H), 8.14 (d, ^3J = 8.9 Hz, 2H, Ar-H), 8.27 (d, ^3J = 8.7 Hz, 2H, Ar-H), 8.65 (s, 1H, CH=N); **^{13}C NMR** (100.6 MHz, $CDCl_3$): δ 14.19, 22.77, 25.95, 26.07, 29.10, 29.19, 29.39, 29.42, 29.43, 29.60, 29.63, 29.64, 29.66, 29.71, 31.99, 68.45, 69.57, 110.52, 112.98, 113.51, 114.40, 114.44, 117.02, 117.70, 117.90, 119.04, 120.04, 120.92, 121.47, 121.53, 122.05, 126.57, 127.34, 127.37, 130.12, 131.72, 132.31, 133.31, 149.49, 150.61, 151.64, 151.96, 152.06, 153.07, 154.76, 155.42, 162.46, 163.26, 163.72, 164.10, 164.15.

3-[4-(3-Chloro-4-n-dodecyloxybenzoyloxy)-2-hydroxybenzylideneamino]phenyl 4-(4-n-dodecyloxybenzoyloxy)benzoate (**OH 2i**)

Following GP2 from 4-formyl-3-hydroxyphenyl 3-chloro-4-*n*-dodecyloxybenzoate (**5c**) and 3-aminophenyl 4-(4-*n*-dodecyloxybenzoyloxy)benzoate (**6a**) [9]; yield: 55%, light-yellow solid (DMF/ethanol); $C_{58}H_{70}O_9NCl$; M_n = 960.60 g/mol; EA: calculated: C 72.51, H 7.35, N 1.46, Cl 3.69; found: C 72.11, H 7.45, N 1.21, Cl 3.89.
^{1}H NMR (400 MHz, $CDCl_3$): δ 0.88 (t, ^3J = 6.4 Hz, 6H, CH_3), 1.26-1.52 (m, 36H, CH_2), 1.78-1.91 (m, 4H, ArOCH$_2$*CH$_2$*), 4.03-4.13 (m, 4H, ArO*CH$_2$*CH$_2$), 6.81 (d, ^3J = 8.1 Hz, 1H, Ar-H), 6.88 (s, 1H, Ar-H), 6.97 (d, ^3J = 8.9 Hz, 4H, Ar-H), 7.18 (m, 3H, Ar-H), 7.37 (d, ^3J = 8.7 Hz, 2H, Ar-H), 7.47 (m, 2H, Ar-H), 8.05 (d, ^3J = 8.7 Hz, 1H, Ar-H), 8.14 (d, ^3J = 8.7 Hz, 2H, Ar-H), 8.19 (s, 1H, Ar-H), 8.28 (d, ^3J = 8.5 Hz, 2H, Ar-H), 8.65 (s, 1H, CH=N). **^{13}C NMR** (100.6 MHz, $CDCl_3$): δ 14.26, 22.84, 26.05, 26.14, 29.06, 29.25, 29.43, 29.49, 29.67, 29.71, 29.78, 29.79, 32.06, 68.52, 69.58, 112.26, 113.10, 114.47, 119.13, 120.12, 120.99, 121.93, 122.14, 123.19, 126.65, 130.21, 130.54, 131.81, 132.23, 132.39, 133.39, 149.58, 151.71, 155.50, 158.99, 162.53, 163.25, 163.79, 164.24.

3-[4-(3-Bromo-4-n-dodecyloxybenzoyloxy)-2-hydroxybenzylideneamino]phenyl 4-(4-n-dodecyloxybenzoyloxy)benzoate (**OH 2j**)

Following GP2 from 4-formyl-3-hydroxyphenyl 3-bromo-4-*n*-dodecyloxy-benzoate (**5d**) and 3-aminophenyl 4-(4-*n*-dodecyloxybenzoyloxy)benzoate (**6a**) [9]; yield: 70%, yellow solid (DMF/ethanol), $C_{58}H_{70}O_9NBr$; M_n = 1005.05 g/mol; EA: calculated: C 69.31, H 7.02, N 1.39, Br 7.95; found: C 68.92, H 7.05, N 1.10, Br 7.94.

^{1}**H NMR** (400 MHz, $CDCl_3$): δ 0.87 (t, ^{3}J = 6.7 Hz, 6H, CH_3), 1.26-1.53 (m, 36H, CH_2), 1.78-1.91 (m, 4H, $ArOCH_2\mathit{CH_2}$), 4.03-4.13 (m, 4H, $ArO\mathit{CH_2}CH_2$), 6.82 (d, ^{3}J = 8.5 Hz, 1H, Ar-H), 6.88 (s, 1H, Ar-H), 6.94 (d, ^{3}J = 8.9 Hz, 1H, Ar-H), 6.97 (d, ^{3}J = 8.9 Hz, 2H, Ar-H), 7.18 (m, 3H, Ar-H), 7.37 (d, ^{3}J = 8.7 Hz, 2H, Ar-H), 7.43 (d, ^{3}J = 8.5 Hz, 1H, Ar-H), 7.47 (t, ^{3}J = 7.9 Hz, 1H, Ar-H), 8.09 (d, ^{3}J = 8.7 Hz, 1H, Ar-H), 8.14 (d, ^{3}J = 8.9 Hz, 2H, Ar-H), 8.27 (d, ^{3}J = 8.7 Hz, 2H, Ar-H), 8.36 (s, 1H, Ar-H), 8.64 (s, 1H, CH=N). ^{13}**C NMR** (100.6 MHz, $CDCl_3$): δ 14.19, 22.77, 26.00, 26.07, 28.99, 29.19, 29.35, 29.42, 29.60, 29.63, 29.66, 29.71, 29.73, 31.99, 68.45, 69.60, 110.55, 112.00, 112.15, 113.00, 114.41, 114.45, 117.04, 119.05, 120.05, 120.93, 122.07, 122.32, 126.58, 130.14, 131.20, 131.73, 132.32, 132.33, 135.31, 149.51, 151.65, 154.76, 155.44, 159.76, 162.48, 163.04, 163.73, 164.12, 164.17.

Compounds H 3 and OH 3 (Table 3)

Bis[4-(4-n-dodecyloxybenzylideneamino)phenyl] isophthalate (**H 3a**)

Following GP1 from 4-(4-*n*-dodecyloxybenzylideneamino)phenol [11] and isophthalic acid; yield: 61%, yellow solid (DMF/ethanol); $C_{58}H_{72}O_6N_2$; M_n = 893.18 g/mol; EA: calculated: C 77.99, H 8.13, N 3.14; found: C 77.49, H 8.12, N 3.00.

^{1}**H NMR** (400 MHz, DMSO-d_6): δ 0.87 (t, ^{3}J = 6.6 Hz, 6H, CH_3), 1.28-1.47 (m, 36H, CH_2), 1.76 (m, 4H, $ArOCH_2\mathit{CH_2}$), 4.11 (t, ^{3}J = 6.4 Hz, 4H, $ArO\mathit{CH_2}CH_2$), 6.66 (d, ^{3}J = 8.4 Hz, 4H, Ar-H), 6.96 (d, ^{3}J = 8.4 Hz, 4H, Ar-H), 7.08 (d, ^{3}J = 8.5 Hz, 4H, Ar-H), 7.78 (t, ^{3}J = 7.9 Hz, 1H, Ar-H), 7.82 (d, ^{3}J = 8.7 Hz, 4H, Ar-H), 8.36 (m, 2H, Ar-H), 8.72 (s, 1H, Ar-H), 9.87 (s, 2H, CH=N). ^{13}**C NMR** (125.7 MHz, DMSO-d_6): δ 12.85, 21.17, 24.68, 27.82, 27.89, 28.12, 28.17, 28.19, 29.62, 30.49, 67.81, 100.78, 113.86, 114.33, 114.72, 115.17, 120.25, 120.79, 121.07, 121.58, 129.43, 130.01, 132.73, 146.50, 163.25, 163.59.

Bis[4-(4-n-dodecyloxybenzylideneamino)phenyl] 4-bromo-isophthalate (**H 3b**)

Following GP1 from 4-(4-*n*-dodecyloxybenzylideneamino)phenol [11] and 4-bromo-isophthalic acid; yield: 54%, yellow solid (DMF/ethanol); $C_{58}H_{71}O_6N_2Br$; M_n = 972.07 g/mol; EA: calculated: C 71.66, H 7.36, N 2.88, Br 8.22; found: C 71.20, H 7.41, N 2.73, Br 8.78:

^{1}**H NMR** (400 MHz, $CDCl_3$): δ 0.88 (t, ^{3}J = 6.8 Hz, 6H, CH_3), 1.27-1.50 (m, 36H, CH_2), 1.80 (m, 4H, $ArOCH_2\mathit{CH_2}$), 4.02 (t, ^{3}J = 6.5 Hz, 4H, $ArO\mathit{CH_2}CH_2$), 6.96 (d, ^{3}J = 8.7 Hz, 4H, Ar-H), 7.27 (m, 8H, Ar-H), 7.83 (d, ^{3}J = 8.6 Hz, 4H, Ar-H), 8.89 (d, ^{3}J = 8.4 Hz, 1H, Ar-H), 8.18 (m, 1H, Ar-H), 8.38 (s, 2H, CH=N), 8.78 (s, 1H, Ar-H). ^{13}**C NMR** (125.7 MHz, $CDCl_3$): δ 14.03, 22.66, 26.04, 29.24, 29.33, 29.38, 29.56, 29.59, 29.63, 29.66, 31.93, 68.37, 114.88, 121.85, 121.87, 122.04, 122.05, 128.68, 129.09, 129.34, 130.63, 132.47, 133.12, 133.88, 135.20, 148.42, 148.47, 150.58, 150.68, 160.00, 162.18, 163.72, 163.83.

Bis[4-(4-n-dodecyloxybenzylideneamino)phenyl] 5-nitro-isophthalate (**H 3c**)

Following GP1 from 4-(4-*n*-dodecyloxybenzylideneamino)phenol [11] and 5-nitro-isophthalic acid; yield: 49%, yellow solid (DMF/ethanol); $C_{58}H_{71}O_8N_3$; M_n = 938.18 g/mol; EA: calculated: C 74.25, H 7.63, N 4.48; found: C 73.83, H 7.72, N 4.34.
^{1}H NMR (500 MHz, $CDCl_3$): δ 0.88 (t, ^3J = 6.9 Hz, 6H, CH_3), 1.27-1.49 (m, 36H, CH_2), 1.81 (m, 4H, ArOCH$_2$*CH$_2$*), 4.02 (t, ^3J = 6.4 Hz, 4H, ArO*CH$_2$*CH$_2$), 6.97 (d, ^3J = 8.9 Hz, 4H, Ar-H), 7.27 (m, 8H, Ar-H), 7.83 (d, ^3J = 8.7 Hz, 4H, Ar-H), 8.39 (s, 2H, CH=N), 9.24 (m, 2H, Ar-H), 9.28 (s, 1H, Ar-H). **^{13}C NMR** (100.6 MHz, $CDCl_3$): δ 14.02, 22.66, 26.04, 29.23, 29.33, 29.38, 29.56, 29.59, 29.63, 29.65, 31.92, 68.38, 114.90, 121.89, 121.97, 125.40, 129.03, 130.65, 132.54, 136.56, 148.18, 148.95, 150.90, 160.15, 162.24, 162.37.

Bis[4-(4-n-dodecyloxy-2-hydroxybenzylideneamino)phenyl] isophthalate (**OH 3a**)

Following GP1 from 4-(4-*n*-dodecyloxy-2-hydroxybenzylideneamino)phenol (**8**) and isophthalic acid; yield: 69%, yellow solid (DMF/ethanol); $C_{58}H_{72}O_8N_2$; M_n = 925.18 g/mol; EA: calculated: C 75.29, H 7.85, N 3.03; found: C 74.98, H 7.88, N 2.87.
^{1}H NMR (400 MHz, $CDCl_3$): δ 0.88 (t, ^3J = 6.3 Hz, 6H, CH_3), 1.29-1.46 (m, 36H, CH_2), 1.75 (m, 4H, ArOCH$_2$*CH$_2$*), 4.06 (t, ^3J = 6.5 Hz, 4H, ArOCH$_2$*CH$_2$*), 6.47 (s, 2H, Ar-H), 6.54 (m, 2H, Ar-H), 7.40 (m, 5H, Ar-H), 7.51 (d, ^3J = 8.7 Hz, 4H, Ar-H), 7.86 (m, 1H, Ar-H), 8.47 (m, 4H, Ar-H), 8.82 (s, 2H, CH=N).

Bis[4-(4-n-dodecyloxy-2-hydroxybenzylideneamino)phenyl] 4-bromo-isophthalate (**OH 3b**)

Following GP1 from 4-(4-*n*-dodecyloxy-2-hydroxybenzylideneamino)phenol (**8**) and 4-bromo-isophthalic acid; yield: 71%, yellow solid (DMF/ethanol); $C_{58}H_{71}O_8N_2Br$; M_n = 1004.07 g/mol. EA: calculated: C 69.38, H 7.13, N 2.79, Br 7.96; found: C 69.00, H 7.43, N 2.47, Br 8.47.
^{1}H NMR (400 MHz, $CDCl_3$): δ 0.87 (t, ^3J = 6.5 Hz, 6H, CH_3), 1.26-1.52 (m, 36H, CH_2), 1.79 (m, 4H, ArOCH$_2$*CH$_2$*), 4.00 (t, ^3J = 6.5 Hz, 4H, ArO*CH$_2$*CH$_2$), 6.49 (d, ^3J = 8.6 Hz, 2H, Ar-H), 6.56 (s, 2H, Ar-H), 7.27 (d, ^3J = 8.9 Hz, 4H, Ar-H), 7.33 (m, 6H, Ar-H), 7.91 (d, ^3J = 8.3 Hz, 1H, Ar-H), 8.19 (d, ^3J = 8.4 Hz, 1H, Ar-H), 8.51 (s, 2H, CH=N), 8.79 (s, 1H, Ar-H).

Bis[4-(4-n-dodecyloxy-2-hydroxybenzylideneamino)phenyl] 5-nitro-isophthalate (**OH 3c**)

Following GP1 from 4-(4-*n*-dodecyloxy-2-hydroxybenzylideneamino)phenol (**8**) and 5-nitro-isophthalic acid; yield: 56%, yellow solid (DMF/ethanol); $C_{58}H_{71}O_{10}N_3$; M_n = 970.18 g/mol calculated: C 71.80, H 7.38, N 4.33; found: C 71.60, H 7.36, N 4.19.
^{1}H NMR (400 MHz, $CDCl_3$): δ 0.87 (t, ^3J = 6.6 Hz, 6H, CH_3), 1.26-1.52 (m, 36H, CH_2), 1.79 (m, 4H, ArOCH$_2$*CH$_2$*), 3.99 (t, ^3J = 6.4 Hz, 4H, ArO*CH$_2$*CH$_2$), 6.49 (m, 2H, Ar-H), 7.30 (m, 10H, Ar-H), 8.53 (s, 2H, CH=N), 9.27 (m, 3H, Ar-H). **^{13}C NMR** (100.6 MHz, $CDCl_3$): δ 14.02, 22.66, 26.02, 29.14, 29.33, 29.36, 29.56, 29.59, 29.63, 29.65, 31.93, 68.42, 101.81, 107.84, 113.02, 122.18, 122.24, 129.12, 132.42, 133.68, 147.29, 148.67, 148.98, 162.21, 162.27, 163.83, 164.00.

Compounds H 4 and OH 4 (Table 4)

1,3-Phenylene bis[4-(4-n-dodecyloxybenzylideneamino)benzoate] (**H 4a**)

Following GP1 from 4-(4-*n*-dodecyloxybenzylideneamino)benzoic acid [11] and resorcinol, yield: 87%, yellow solid (DMF/ethanol); $C_{58}H_{72}O_6N_2$; M_n = 893.18 g/mol; EA: calculated: C 77.99, H 8.13, N 3.14; found: C 77.53, H 8.16, N 3.17.
^{1}H NMR (400 MHz, $CDCl_3$): δ 0.87 (t, ^3J = 6.8 Hz, 6H, CH_3), 1.26-1.46 (m, 36H, CH_2), 1.80 (m, 4H, ArOCH$_2$*CH$_2$*), 4.02 (t, ^3J = 6.5 Hz, 4H, ArO*CH$_2$*CH$_2$), 6.97 (d, ^3J = 8.7 Hz, 4H, Ar-H), 7.17 (m, 3H, Ar-H), 7.23 (d, ^3J = 8.7 Hz, 4H, Ar-H), 7.46 (m, 1H, Ar-H), 7.84 (d, ^3J = 8.7 Hz, 4H, Ar-H), 8.20 (d, ^3J = 8.7 Hz, 4H, Ar-H), 8.36 (s, 2H, CH=N).

4-Bromo-1,3-phenylene bis[4-(4-n-dodecyloxybenzylideneamino)benzoate] (**H 4b**)

Following GP1 from 4-(4-*n*-dodecyloxybenzylideneamino)benzoic acid [11] and 4-bromoresorcinol, yield: 69%, yellow solid (DMF/ethanol); $C_{58}H_{71}O_6N_2Br$; M_n = 972.07 g/mol.
^{1}H NMR (400 MHz, $CDCl_3$): δ 0.87 (t, ^3J = 6.6 Hz, 6H, CH_3), 1.26-1.50 (m, 36H, CH_2), 1.80 (m, 4H, ArOCH$_2$*CH$_2$*), 4.02 (t, ^3J = 6.5 Hz, 4H, ArO*CH$_2$*CH$_2$), 6.97 (d, ^3J = 8.7 Hz, 4H, Ar-H), 7.10 (m, 1H, Ar-H), 7.24 (d, ^3J = 8.5 Hz, 4H, Ar-H), 7.29 (m, 1H, Ar-H), 7.68 (d, ^3J = 8.7 Hz, 1H, Ar-H), 7.84 (d, ^3J = 8.7 Hz, 4H, Ar-H), 8.18 (d, ^3J = 8.3 Hz, 2H, Ar-H), 8.24 (d, ^3J = 8.3 Hz, 2H, Ar-H), 8.36 (s, 2H, CH=N).

2-Methyl-1,3-phenylene bis[4-(4-n-dodecyloxybenzylideneamino)benzoate] (**H 4c**)

Following GP1 from 4-(4-*n*-dodecyloxybenzylideneamino)benzoic acid [11] and 2-methylresorcinol, yield: 66%, yellow solid (DMF/ethanol); $C_{59}H_{74}O_6N_2$; M_n = 907.20 g/mol.
^{1}H NMR (400 MHz, $CDCl_3$): δ 0.87 (t, ^3J = 6.8 Hz, 6H, CH_3), 1.26-1.50 (m, 36H, CH_2), 1.80 (m, 4H, ArOCH$_2$*CH$_2$*), 2.13 (s, 3H, Ar-CH$_3$), 4.02 (t, ^3J = 6.6 Hz, 4H, ArO*CH$_2$*CH$_2$), 6.97 (d, ^3J = 8.7 Hz, 4H, Ar-H), 7.12 (d, ^3J = 8.3 Hz, 2H, Ar-H), 7.25 (d, ^3J = 8.5 Hz, 4H, Ar-H), 7.31 (t, ^3J = 8.2 Hz, 1H, Ar-H), 7.34 (d, ^3J = 8.7 Hz, 4H, Ar-H), 8.23 (d, ^3J = 8.5 Hz, 4H, Ar-H), 8.36 (s, 2H, CH=N). **^{13}C NMR** (100.6 MHz, $CDCl_3$): δ 10.24, 14.18, 22.77, 26.10, 29.27, 29.42, 29.46, 29.64, 29.67, 29.71, 29.74, 32.00, 68.36, 114.81, 119.82, 120.92, 123.97, 125.90, 126.44, 128.56, 130.84, 131.45, 150.34, 157.36, 160.96, 162.33, 164.27.

5-Methoxy-1,3-phenylene bis[4-(4-n-dodecyloxybenzylideneamino)benzoate] (**H 4d**)

Following GP1 from 4-(4-*n*-dodecyloxybenzylideneamino)benzoic acid [11] and 5-methoxyresorcinol, yield: 49%, yellow solid (DMF/ethanol); $C_{59}H_{74}O_7N_2$; M_n = 923.20 g/mol;
^{1}H NMR (400 MHz, $CDCl_3$): δ 0.87 (t, ^3J = 6.6 Hz, 6H, CH_3), 1.26-1.48 (m, 36H, CH_2), 1.80 (m, 4H, ArOCH$_2$*CH$_2$*), 3.83 (s, 3H, Ar-OCH$_3$), 4.02 (t, ^3J = 6.5 Hz, 4H, ArO*CH$_2$*CH$_2$), 6.73

(s, 2H, Ar-H), 6.80 (s, 1H, Ar-H), 6.97 (d, ^3J = 8.7 Hz, 4H, Ar-H), 7.23 (d, ^3J = 8.1 Hz, 4H, Ar-H), 7.83 (d, ^3J = 8.7 Hz, 4H, Ar-H), 8.18 (d, ^3J = 8.3 Hz, 4H, Ar-H), 8.36 (s, 2H, CH=N).

4-Bromo-1,3-phenylene bis[4-(4-n-dodecyloxy-2-hydroxybenzylideneamino)benzoate] (**OH 4b**)

Following GP1 from 4-(4-*n*-dodecyloxybenzylideneamino)benzoic acid [11] and 4-bromoresorcinol, yield: 61%, yellow solid (DMF/ethanol); $C_{58}H_{71}O_8N_2Br$, M_n = 1004.07 g/mol. EA: calculated: C 69.38, H 7.13, N 2.79, Br 7.96; found: C 69.03, H 7.20, N 2.58, Br 8.01.

^{1}H NMR (400 MHz, $CDCl_3$): δ 0.87 (t, ^3J = 7.1 Hz, 6H, CH_3), 1.26-1.53 (m, 36H, CH_2), 1.79 (m, 4H, $ArOCH_2\mathit{CH_2}$), 4.00 (t, ^3J = 6.5 Hz, 4H, $ArO\mathit{CH_2}CH_2$), 6.49 (m, 4H, Ar-H), 7.10 (d, ^3J = 8.7 Hz, 1H, Ar-H), 7.31 (m, 7H, Ar-H), 7.69 (d, ^3J = 8.7 Hz, 1H, Ar-H), 8.20 (d, ^3J = 8.7 Hz, 2H, Ar-H), 8.27 (d, ^3J = 8.7 Hz, 2H, Ar-H), 8.55 (s, 2H, CH=N). **^{13}C NMR** (100.6 MHz, $CDCl_3$): δ 14.26, 22.84, 26.13, 29.21, 29.48, 29.69, 29.72, 29.77, 29.79, 32.06, 68.49, 101.64, 108.16, 112.92, 117.93, 121.29, 121.32, 131.72, 131.94, 133.38, 134.00, 150.54, 163.08, 163.36, 164.30.

5-Methoxy-1,3-phenylene bis[4-(4-n-dodecyloxy-2-hydroxybenzylideneamino)benzoate] (**OH 4d**)

Following GP1 from 4-(4-*n*-dodecyloxybenzylideneamino)benzoic acid [11] and 4-5-methoxyresorcinol, yield: 65%, yellow solid (DMF/ethanol), $C_{59}H_{74}O_9N_2$; M_n = 955.20 g/mol; EA: calculated: C 74.18, H 7.81, N 2.93; found: C 73.78, H 7.87, N 2.84.

^{1}H NMR (400 MHz, $CDCl_3$): δ 0.87 (t, ^3J = 6.8 Hz, 6H, CH_3), 1.26-1.53 (m, 36H, CH_2), 1.79 (m, 4H, $ArOCH_2\mathit{CH_2}$), 3.83 (s, 3H, OCH_3), 4.00 (t, ^3J = 6.5 Hz, 4H, $ArO\mathit{CH_2}CH_2$), 6.50 (m, 4H, Ar-H), 7.23 (s, 2H, Ar-H), 6.80 (s, 1H, Ar-H), 7.28 (d, ^3J = 9.3 Hz, 2H, Ar-H), 7.32 (d, ^3J = 8.5 Hz, 4H, Ar-H), 8.21 (d, ^3J = 8.5 Hz, 4H, Ar-H), 8.55 (s, 2H, CH=N). **^{13}C NMR** (100.6 MHz, $CDCl_3$): δ 14.26, 22.84, 26.13, 29.21, 29.48, 29.69, 29.73, 29.77, 29.79, 32.06, 55.86, 68.48, 101.65, 105.66, 108.13, 108.18, 112.82, 121.22, 126.79, 131.68, 133.97, 152.01, 153.33, 160.86, 163.00, 164.04, 164.16, 164.26.

Compounds H 5 and OH 5 (Table 5)

N,N'-Bis[4-(4-n-dodecyloxybenzoyloxy)benzylidene]-2-methyl-1,3-phenylenediamine (**H 5b**)

Following GP2 from 4-formylphenyl 4-*n*-dodecyloxybenzoate [8] and 2-methyl-1,3-phenylenediamine. yield: 93%, light yellow solid (DMF/ethanol), $C_{59}H_{74}O_6N_2$; M_n = 907.20 g/mol; EA: calculated: C 78.11, H 8.22, N 3.09; found: C 78.00, H 8.24, N 3.02.

^{1}H NMR (400 MHz, $CDCl_3$): δ 0.87 (t, ^3J = 6.8 Hz, 6H, CH_3), 1.26-1.48 (m, 36H, CH_2), 1.81 (m, 4H, $ArOCH_2\mathit{CH_2}$), 2.36 (s, 3H, Ar-CH_3), 4.04 (t, ^3J = 6.5 Hz, 4H, $ArO\mathit{CH_2}CH_2$), 6.81 (d, ^3J = 7.7 Hz, 2H, Ar-H), 6.96 (d, ^3J = 9.1 Hz, 4H, Ar-H), 7.21 (t, ^3J = 7.8 Hz, 1H, Ar-H), 7.32 (d, ^3J = 8.5 Hz, 4H, Ar-H), 7.99 (d, ^3J = 8.7 Hz, 4H, Ar-H), 8.14 (d, ^3J = 8.9 Hz, 4H, Ar-H),

8.38 (s, 2H, CH=N). **^{13}C NMR** (100.6 MHz, $CDCl_3$): δ 12.54, 14.15, 22.74, 26.04, 29.17, 29.39, 29.41, 29.60, 29.64, 29.69, 29.71, 31.97, 68.43, 114.40, 115.24, 121.32, 122.23, 125.54, 126.81, 129.95, 132.35, 134.06, 151.76, 153.47, 158.56, 163.69, 164.56.

N,N'-Bis[4-(4-n-dodecyloxybenzoyloxy)benzylidene]-4-methyl-1,3-phenylenediamine (**H 5d**)
Following GP2 from 4-formylphenyl 4-*n*-dodecyloxybenzoate [8] and 4-methyl-1,3-phenylenediamine. yield: 72%, yellow solid (DMF/ethanol); $C_{59}H_{74}O_6N_2$, M_n = 907.20 g/mol. EA: calculated: C 78.11, H 8.22, N 3.09; found: C 77.93, H 8.24, N 2.97.
^{1}H NMR (400 MHz, $CDCl_3$): δ 0.87 (t, ^3J = 6.8 Hz, 6H, CH_3), 1.26-1.48 (m, 36H, CH_2), 1.81 (m, 4H, $ArOCH_2\mathit{CH_2}$), 2.37 (s, 3H, Ar-CH_3), 4.04 (t, ^3J = 6.6 Hz, 4H, $ArO\mathit{CH_2}CH_2$), 6.96 (d, ^3J = 8.9 Hz, 4H, Ar-H), 7.02 (m, 1H, Ar-H), 7.16 (m, 2H, Ar-H), 7.32 (d, ^3J = 8.6 Hz, 4H, Ar-H), 7.96 (d, ^3J = 8.7 Hz, 2H, Ar-H), 7.98 (d, ^3J = 8.7 Hz, 2H, Ar-H), 8.14 (d, ^3J = 8.9 Hz, 4H, Ar-H), 8.43 (s, 1H, CH=N), 8.51 (s, 1H, CH=N). **^{13}C NMR** (100.6 MHz, $CDCl_3$): δ 14.15, 17.53, 22.74, 26.04, 29.17, 29.39, 29.41, 29.60, 29.64, 29.68, 29.70, 31.97, 68.43, 110.56, 114.40, 117.82, 121.30, 122.22, 122.25, 129.92, 129.98, 130.86, 132.33, 133.87, 133.98, 150.62, 151.59, 153.52, 158.53, 158.61, 163.69, 164.53.

N,N'-Bis[4-(4-n-dodecyloxy-3-fluorobenzoyloxy)benzylidene]-1,3-phenylenediamine (**H 5e**)

Following GP2 from 4-formylphenyl 4-*n*-dodecyloxy-3-fluoro-benzoate [2] and 1,3-phenylenediamine. yield: 66%, yellow solid (DMF/ethanol); $C_{58}H_{70}O_6N_2F_2$, M_n = 929.16 g/mol.
^{1}H NMR (400 MHz, $CDCl_3$): δ 0.87 (t, ^3J = 6.8 Hz, 6H, CH_3), 1.25-1.49 (m, 36H, CH_2), 1.86 (m, 4H, $ArOCH_2\mathit{CH_2}$), 4.11 (t, ^3J = 6.5 Hz, 4H, $ArO\mathit{CH_2}CH_2$), 6.95 (m, 2H, Ar-H), 7.02 (s, 1H, Ar-H), 7.09 (m, 2H, Ar-H), 7.32 (d, ^3J = 8.7 Hz, 4H, Ar-H), 7.40 (t, ^3J = 7.8 Hz, 1H, Ar-H), 7.89 (m, 2H, Ar-H), 7.94 (s, 2H, Ar-H), 7.97 (d, ^3J = 8.7 Hz, 4H, Ar-H), 8.51 (s, 2H, CH=N).

N,N'-Bis[4-(3-chloro-4-n-dodecyloxybenzoyloxy)benzylidene]-1,3-phenylenediamine (**H 5f**)

Following GP2 from 4-formylphenyl 3-chloro-4-*n*-dodecyloxybenzoate (**1c**) and 1,3-phenylenediamine. yield: 67%, light-yellow solid (DMF/ethanol); $C_{58}H_{70}O_6N_2Cl_2$, M_n = 962.04 g/mol.
^{1}H NMR (400 MHz, $CDCl_3$): δ 0.87 (t, ^3J = 6.6 Hz, 6H, CH_3), 1.25-1.52 (m, 36H, CH_2), 1.87 (m, 4H, $ArOCH_2\mathit{CH_2}$), 4.11 (t, ^3J = 6.6 Hz, 4H, $ArO\mathit{CH_2}CH_2$), 6.98 (d, ^3J = 8.7 Hz, 2H, Ar-H), 7.15 (m, 3H, Ar-H), 7.32 (d, ^3J = 8.5 Hz, 4H, Ar-H), 7.39 (t, ^3J = 7.3 Hz, 1H, Ar-H), 7.97 (d, ^3J = 8.7 Hz, 4H, Ar-H), 8.06 (m, 2H, Ar-H), 8.20 (s, 2H, Ar-H), 8.51 (s, 2H, CH=N).

N,N'-Bis[4-(3-bromo-4-n-dodecyloxybenzoyloxy)benzylidene]-1,3-phenylenediamine (**H 5g**)

Following GP2 from 4-formylphenyl 3-bromo-4-*n*-dodecyloxybenzoate (**1d**) and 1,3-phenylenediamine. Yield: 66%, yellow solid (DMF/ethanol); $C_{58}H_{70}O_6N_2Br_2$; M_n = 1050.97 g/mol.

^{1}**H NMR** (400 MHz, $CDCl_3$): δ 0.87 (t, ^3J = 6.7 Hz, 6H, CH_3), 1.26-1.53 (m, 36H, CH_2), 1.86 (m, 4H, $ArOCH_2$*CH_2*), 4.11 (t, ^3J = 6.5 Hz, 4H, ArO*CH_2*CH_2), 6.94 (d, ^3J = 8.7 Hz, 2H, Ar-H), 7.19 (m, 3H, Ar-H), 7.32 (d, ^3J = 8.5 Hz, 4H, Ar-H), 7.39 (m, 1H, Ar-H), 7.97 (d, ^3J = 8.7 Hz, 4H, Ar-H), 8.10 (m, 2H, Ar-H), 8.38 (s, 2H, Ar-H), 8.51 (s, 2H, CH=N). ^{13}**C NMR** (100.6 MHz, $CDCl_3$): δ 14.20, 22.77, 26.00, 28.98, 29.35, 29.42, 29.60, 29.64, 29.72, 31.99, 69.59, 109.59, 111.99, 118.61, 122.01, 122.09, 122.41, 129.89, 130.02, 131.19, 133.84, 135.29, 152.79, 153.23, 159.26, 159.75, 163.33.

N,N'-Bis[4-(4-n-dodecyloxybenzoyloxy)-2-hydroxybenzylidene]-1,3-phenylenediamine (**OH 5a**)

Following GP2 from 4-formyl-3-hydroxyphenyl 4-*n*-dodecyloxy-benzoate [10] and 1,3-phenylenediamine, yield: 86%, light-yellow solid (DMF/ethanol); $C_{58}H_{72}O_8N_2$, M = 925.18 g/mol. EA: calculated: C 75.29, H 7.85, N 3.03; found: C 74.92, H 7.83, N 2.86.
^{1}**H NMR** (400 MHz, $CDCl_3$): δ 0.87 (t, ^3J = 6.8 Hz, 6H, CH_3), 1.24-1.52 (m, 36H, CH_2), 1.81 (m, 4H, $ArOCH_2$*CH_2*), 4.04 (t, ^3J = 6.5 Hz, 4H, ArO*CH_2*CH_2), 6.84 (d, ^3J = 8.4 Hz, 2H, Ar-H), 6.89 (s, 2H, Ar-H), 6.96 (d, ^3J = 9.1 Hz, 4H, Ar-H), 7.20 (m, 4H, Ar-H), 7.44 (d, ^3J = 8.3 Hz, 2H, Ar-H), 8.12 (d, ^3J = 8.9 Hz, 4H, Ar-H), 8.67 (s, 2H, CH=N).

N,N'-Bis[4-(4-n-dodecyloxybenzoyloxy)-2-hydroxybenzylidene]-2-methyl-1,3-phenylenediamine (**OH 5b**)

Following GP2 from 4-formyl-3-hydroxyphenyl 4-*n*-dodecyloxy-benzoate [10] and 2-methyl-1,3-phenylenediamine. Yield: 70%, light-yellow solid (DMF/ethanol); $C_{59}H_{74}O_8N_2$, M_n: 939.20 g/mol. EA: calculated: C 75.45, H 7.94, N 2.98; found: C 75.49, H 7.90, N 2.85.
^{1}**H NMR** (400 MHz, $CDCl_3$): δ 0.87 (t, ^3J = 6.8 Hz, 6H, CH_3), 1.24-1.54 (m, 36H, CH_2), 1.81 (m, 4H, $ArOCH_2$*CH_2*), 2.41 (s, 3H, Ar-CH_3), 4.03 (t, ^3J = 6.5 Hz, 4H, ArO*CH_2*CH_2), 6.84 (d, ^3J = 8.5 Hz, 2H, Ar-H), 6.90 (s, 2H, Ar-H), 6.96 (d, ^3J = 9.1 Hz, 4H, Ar-H), 7.02 (d, ^3J = 7.9 Hz, 2H, Ar-H), 7.31 (t, ^3J = 7.8 Hz, 1H, Ar-H), 7.44 (d, ^3J = 8.5 Hz, 2H, Ar-H), 8.12 (d, ^3J = 8.9 Hz, 4H, Ar-H), 8.57 (s, 2H, CH=N).

N,N'-Bis[4-(4-n-dodecyloxy-3-fluorobenzoyloxy)-2-hydroxybenzylidene]-2-methyl-1,3-phenylenediamine (**OH 5c**)

Following GP2 from 4-formyl-3-hydroxyphenyl 4-*n*-dodecyloxy-3-fluorobenzoate (**5b**) and 2-methyl-1,3-phenylenediamine. Yield: 76%, yellow solid (DMF/Ethanol); $C_{59}H_{72}O_8N_2F_2$, M = 975.18 g/mol. EA: calculated: C 72.66, H 7.44, N 2.87; found: C 72.24, H 7.95, N 2.49.
^{1}**H NMR** (400 MHz, $CDCl_3$): δ 0.88 (t, ^3J = 6.1 Hz, 6H, CH_3), 1.27-1.50 (m, 36H, CH_2), 1.85 (m, 4H, $ArOCH_2$*CH_2*), 2.41 (s, 3H, Ar-CH_3), 4.12 (t, ^3J = 6.3 Hz, 4H, ArO*CH_2*CH_2), 6.83 (d, ^3J = 8.3 Hz, 2H, Ar-H), 6.90 (s, 2H, Ar-H), 7.01 (d, ^3J = 8.1 Hz, 2H, Ar-H), 7.24 (s, 2H, Ar-H), 7.30 (t, ^3J = 7.9 Hz, 1H, Ar-H), 7.43 (d, ^3J = 8.5 Hz, 2H, Ar-H), 7.87 (m, 2H, Ar-H), 7.93 (d, ^3J = 8.5 Hz, 2H, Ar-H), 8.56 (s, 2H, CH=N). ^{13}**C NMR** (100.6 MHz, $CDCl_3$): δ 12.87, 14.07, 22.71, 25.95, 29.15, 29.37, 29.57, 29.62, 29.67, 29.69, 31.97, 68.79, 110.62, 112.99, 113.92, 116.67, 117.38, 117.86, 118.06, 121.88, 126.13, 127.40, 127.43, 133.21, 148.86, 150.93, 152.15, 152.25, 153.39, 159.8, 162.44, 162.76, 163.36.

N,N'-Bis[4-(4-n-dodecyloxybenzoyloxy)-2-hydroxybenzylidene]-4-methyl-1,3-phenylenediamine (**OH 5d**)

Following GP2 from 4-formyl-3-hydroxyphenyl 4-*n*-dodecyloxy-benzoate [10] and 4-methyl-1,3-phenylenediamine. Yield 70%, yellow solid (DMF/ethanol); $C_{59}H_{74}O_8N_2$; M_n = 939.20 g/mol. EA: calculated: C 75.45, H 7.94, N 2.98; found: C 75.23, H 7.95, N 2.85.
^{1}H NMR (400 MHz, $CDCl_3$): δ 0.87 (t, ^3J = 6.8 Hz, 6H, CH_3), 1.26-1.53 (m, 36H, CH_2), 1.81 (m, 4H, $ArOCH_2\mathit{CH_2}$), 2.41 (s, 3H, Ar-CH_3), 4.03 (t, ^3J = 6.5 Hz, 4H, $ArO\mathit{CH_2}CH_2$), 6.83 (m, 2H, Ar-H), 6.89 (s, 2H, Ar-H), 6.96 (d, ^3J = 8.7 Hz, 4H, Ar-H), 7.04 (s, 1H, Ar-H), 7.13 (d, ^3J = 8.0 Hz, 1H, Ar-H), 7.30 (d, ^3J = 8.3 Hz, 1H, Ar-H), 7.44 (m, 2H, Ar-H), 8.12 (d, ^3J = 8.8 Hz, 4H, Ar-H), 8.67 (s, 2H, CH=N).

N,N'-Bis[4-(4-n-dodecyloxy-3-fluorobenzoyloxy)-2-hydroxybenzylidene]-1,3-phenylenediamine (**OH 5e**)

Following GP2 from 4-formyl-3-hydroxyphenyl 4-*n*-dodecyloxy-3-fluorobenzoate (**5b**) and 1,3-phenylenediamine. Yield: 62%, light-yellow solid (DMF/ethanol). $C_{58}H_{70}O_8N_2F_2$. M_n = 961.16 g/mol. EA: calculated: C 72.47, H 7.34, N 2.92; found: C 72.30, H 7.79, N 2.43.
^{1}H NMR (400 MHz, $CDCl_3$): δ 0.87 (t, ^3J = 6.8 Hz, 6H, CH_3), 1.26-1.52 (m, 36H, CH_2), 1.86 (m, 4H, $ArOCH_2\mathit{CH_2}$), 4.11 (t, ^3J = 6.5 Hz, 4H, $ArO\mathit{CH_2}CH_2$), 6.83 (d, ^3J = 8.4 Hz, 2H, Ar-H), 6.89 (s, 2H, Ar-H), 7.02 (m, 2H, Ar-H), 7.21 (m, 4H, Ar-H), 7.45 (d, ^3J = 8.5 Hz, 2H, Ar-H), 7.93 (m, 4H, Ar-H), 8.67 (s, 2H, CH=N). **^{13}C NMR** (100.6 MHz, $CDCl_3$): δ 14.21, 22.78, 25.96, 29.09, 29.40, 29.42, 29.61, 29.65, 29.71, 29.73, 31.99, 69.55, 110.57, 113.03, 113.47, 117.06, 117.70, 117.91, 119.62, 127.40, 130.30, 133.29, 149.43, 150.60, 152.09, 154.76, 162.33, 162.51, 163.32.

N,N'-Bis[4-(3-chloro-4-n-dodecyloxybenzoyloxy)-2-hydroxybenzylidene]-1,3-phenylenediamine (**OH 5f**)

Following GP2 from 4-formyl-3-hydroxyphenyl 3-chloro-4-*n*-dodecyloxybenzoate (**5c**) and 1,3-phenylenediamine.yield: 80%, light-yellow solid (DMF/ethanol); $C_{58}H_{70}O_8N_2Cl_2$; M_n = 994.04 g/mol. EA: calculated: C 70.08, H 7.10, N 2.82, Cl 7.13; found: C 69.77, H 7.10, N 2.64, Cl 7.22.
^{1}H NMR (400 MHz, $CDCl_3$): δ 0.87 (t, ^3J = 6.8 Hz, 6H, CH_3), 1.26-1.54 (m, 36H, CH_2), 1.87 (m, 4H, $ArOCH_2\mathit{CH_2}$), 4.11 (t, ^3J = 6.5 Hz, 4H, $ArO\mathit{CH_2}CH_2$), 6.82 (d, ^3J = 8.4 Hz, 2H, Ar-H), 6.89 (s, 2H, Ar-H), 6.97 (d, ^3J = 8.7 Hz, 2H, Ar-H), 7.21 (m, 4H, Ar-H), 7.44 (d, ^3J = 8.5 Hz, 2H, Ar-H), 8.04 (d, ^3J = 8.6 Hz, 2H, Ar-H), 8.19 (s, 2H, Ar-H), 8.67 (s, 2H, CH=N).
^{13}C NMR (100.6 MHz, $CDCl_3$): δ 14.19, 22.77, 25.99, 29.00, 29.37, 29.42, 29.61, 29.64, 29.72, 29.73, 32.00, 69.54, 110.58, 112.22, 113.02, 113.84, 117.08, 119.62, 121.86, 123.14, 130.48, 132.17, 133.29, 149.45, 154.78, 158.95, 162.33, 162.53, 163.21.

N,N'-Bis[4-(3-bromo-4-n-dodecyloxybenzoyloxy)-2-hydroxybenzylidene]-1,3-phenylenediamine (**OH 5g**)

Following GP2 from 4-formyl-3-hydroxyphenyl 3-bromo-4-*n*-dodecyloxybenzoate (**5d**) and 1,3-phenylenediamine. Yield: 63%, yellow solid (DMF/ethanol). $C_{58}H_{70}O_8N_2Br_2$, M_n = 1082.97 g/mol. EA: calculated: C 64.32, H 6.52, N 2.59, Br 14.76; found: C 64.06, H 6.63, N 2.48, Br 15.32.
^{1}H NMR (400 MHz, $CDCl_3$): δ 0.87 (t, ^3J = 7.5 Hz, 6H, CH_3), 1.26-1.54 (m, 36H, CH_2), 1.87 (m, 4H, ArOCH$_2$*CH$_2$*), 4.11 (t, ^3J = 6.5 Hz, 4H, ArO*CH$_2$*CH$_2$), 6.83 (d, ^3J = 8.4 Hz, 2H, Ar-H), 6.89 (s, 2H, Ar-H), 6.94 (d, ^3J = 8.7 Hz, 2H, Ar-H), 7.21 (m, 4H, Ar-H), 7.45 (d, ^3J = 8.5 Hz, 2H, Ar-H), 8.09 (d, ^3J = 8.7 Hz, 2H, Ar-H), 8.37 (s, 2H, Ar-H), 8.67 (s, 2H, CH=N).

Compounds H 6 and OH 6 (Table 6)

N,N'-Bis[4-(4-n-dodecyloxycinnamoyloxy)benzylidene]-1,3-phenylenediamine (**H 6a**)

Following GP2 from 4-formylphenyl 4-*n*-dodecyloxycinnamate [12] and 1,3-phenylenediamine. Yield: 91%, yellow solid (DMF/ethanol); $C_{62}H_{76}O_6N_2$, M = 945.25 g/mol. EA: calculated: C 78.77, H 8.11, N 2.96; found: C 78.74, H 8.11, N 2.81.
^{1}H NMR (400 MHz, $CDCl_3$): δ 0.89 (t, ^3J = 6.8 Hz, 6H, CH_3), 1.26-1.45 (m, 36H, CH_2), 1.79 (m, 4H, ArOCH$_2$*CH$_2$*), 3.99 (t, ^3J = 6.5 Hz, 4H, ArO*CH$_2$*CH$_2$), 6.48 (d, ^3J = 15.8 Hz, 2H, Ar-CH=*CH*), 6.91 (d, ^3J = 8.7 Hz, 4H, Ar-H), 7.08 (m, 3H, Ar-H), 7.29 (d, ^3J = 8.5 Hz, 4H, Ar-H), 7.40 (m, 1H, Ar-H), 7.52 (d, ^3J = 8.9 Hz, 4H, Ar-H), 7.83 (d, ^3J = 16.0 Hz, 2H, Ar-*CH*=CH), 7.95 (d, ^3J = 8.7 Hz, 4H, Ar-H), 8.50 (s, 2H, CH=N).

N,N'-Bis[4-(4-n-dodecyloxycinnamoyloxy)benzylidene]-2-methyl-1,3-phenylenediamine (**H6b**)

Following GP2 from 4-formylphenyl 4-*n*-dodecyloxycinnamate [12] and 2-methyl-1,3-phenylenediamine. Yield: 70%, yellow solid (DMF/Ethanol); $C_{63}H_{78}O_6N_2$, M_n = 959.27 g/mol. EA: calculated: C 78.86, H 8.20, N 2.92; found: C 78.63, H 8.25, N 2.83.
^{1}H NMR (400 MHz, $CDCl_3$): δ 0.87 (t, ^3J = 6.8 Hz, 6H, CH_3), 1.25-1.48 (m, 36H, CH_2), 1.79 (m, 4H, ArOCH$_2$*CH$_2$*), 2.35 (s, 3H, CH_3), 3.99 (t, ^3J = 6.6 Hz, 4H, ArO*CH$_2$*CH$_2$), 6.48 (d, ^3J = 15.8 Hz, 2H, Ar-CH=*CH*), 6.80 (m, 2H, Ar-H), 6.91 (d, ^3J = 8.7 Hz, 4H, Ar-H), 7.20 (t, ^3J = 7.9 Hz, 1H, Ar-H), 7.28 (d, ^3J = 8.5 Hz, 4H, Ar-H), 7.52 (d, ^3J = 8.7 Hz, 4H, Ar-H), 7.83 (d, ^3J = 15.8 Hz, 2H, Ar-*CH*=CH), 7.97 (d, ^3J = 8.7 Hz, 4H, Ar-H), 8.37 (s, 1H, CH=N). **^{13}C NMR** (100.6 MHz, $CDCl_3$): δ 12.53, 14.15, 22.74, 26.06, 29.22, 29.39, 29.41, 29.61, 29.64, 29.68, 29.70, 31.97, 69.30, 114.16, 115.01, 115.22, 122.10, 122.43, 125.52, 126.64, 126.81, 129.93, 130.08, 130.17, 131.16, 134.01, 146.79, 151.76, 153.30, 158.55, 161.51, 165.35.

N,N'-Bis[4-(4-n-dodecyloxycinnamoyloxy)benzylidene]-4-methyl-1,3-phenylenediamine (**H 6c**)

Following GP2 from 4-formylphenyl 4-*n*-dodecyloxycinnamate [12] and 4-methyl-1,3-phenylenediamine. Yield: 73%, yellow solid (DMF/ethanol), $C_{63}H_{78}O_6N_2$, M_n = 959.27 g/mol. EA: calculated: C 78.86, H 8.20, N 2.92; found: C 78.62, H 8.21, N 2.89.
^{1}H NMR (400 MHz, $CDCl_3$): δ 0.87 (t, ^3J = 6.8 Hz, 6H, CH_3), 1.25-1.48 (m, 36H, CH_2), 1.79 (m, 4H, ArOCH$_2$*CH$_2$*), 2.36 (s, 3H, CH_3), 3.99 (t, ^3J = 6.5 Hz, 4H, ArO*CH$_2$*CH$_2$), 6.48 (d, ^3J = 15.9 Hz, 2H, Ar-CH=*CH*), 6.83 (m, 1H, Ar-H), 6.91 (d, ^3J = 8.1 Hz, 4H, Ar-H), 7.01 (m, 1H, Ar-H), 7.22 (s, 1H, Ar-H), 7.28 (d, ^3J = 8.7 Hz, 4H, Ar-H), 7.52 (d, ^3J = 8.8 Hz, 4H, Ar-H), 7.83 (d, ^3J = 15.8 Hz, 2H, Ar-*CH*=CH), 7.94 (d, ^3J = 8.7 Hz, 2H, Ar-H), 7.96 (d, ^3J = 8.7 Hz, 2H, Ar-H), 8.42 (s, 1H, CH=N), 8.50 (s, 1H, CH=N). **^{13}C NMR** (100.6 MHz, $CDCl_3$): δ 14.15, 17.52, 22.74, 26.06, 29.22, 29.39, 29.41, 29.61, 29.64, 29.68, 29.70, 31.97, 68.30, 109.88, 110.55, 114.15, 115.01, 117.80, 122.09, 122.12, 126.63, 129.90, 129.96, 130.09, 130.85, 130.82, 133.93, 146.79, 150.62, 151.60, 153.30, 158.52, 158.60, 161.51, 165.30, 165.35.

N,N'-Bis[4-(4-n-dodecyloxycinnamoyloxy)-2-hydroxybenzylidene]-1,3-phenylenediamine (**OH 6a**)

Following GP2 from 4-formyl-3-hydroxyphenyl 4-*n*-dodecyloxycinnamate (**9**) and 1,3-phenylenediamine. Yield: 78%, yellow solid (DMF/ethanol). $C_{62}H_{76}O_8N_2$, M_n = 977.25 g/mol. EA: calculated: C 76.20, H 7.84, N 2.87; found: C 75.92, H 7.88, N 2.71.
^{1}H NMR (400 MHz, $CDCl_3$): δ 0.88 (t, ^3J = 6.8 Hz, 6H, CH_3), 1.27-1.48 (m, 36H, CH_2), 1.78 (m, 4H, ArOCH$_2$*CH$_2$*), 4.00 (t, ^3J = 6.5 Hz, 4H, ArO*CH$_2$*CH$_2$), 6.46 (d, ^3J = 16.0 Hz, 2H, Ar-CH=*CH*), 6.81 (d, ^3J = 8.4 Hz, 2H, Ar-H), 6.86 (s, 2H, Ar-H), 6.91 (d, ^3J = 8.7 Hz, 4H, Ar-H), 7.17 (m, 2H, Ar-H), 7.43 (m, 4H, Ar-H), 7.52 (d, ^3J = 8.7 Hz, 4H, Ar-H), 7.82 (d, ^3J = 15.8 Hz, 2H, Ar-*CH*=CH), 8.66 (s, 2H, CH=N).

N,N'-Bis[4-(4-n-dodecyloxycinnamoyloxy)-2-hydroxybenzylidene]-2-methyl-1,3-phenylenediamine (**OH 6b**)

Following GP2 from 4-formyl-3-hydroxyphenyl 4-*n*-dodecyloxycinnamate (**9**) and 2-methyl-1,3-phenylenediamine. Yield: 61%, yellow solid (DMF/ethanol). $C_{63}H_{78}O_8N_2$, M_n = 991.27 g/mol. EA: calculated: C 76.33, H 7.93, N 2.83; found: C 75.99, H 7.98, N 2.69.
^{1}H NMR (400 MHz, $CDCl_3$): δ 0.85 (t, ^3J = 6.8 Hz, 6H, CH_3), 1.24-1.53 (m, 36H, CH_2), 1.78 (m, 4H, ArOCH$_2$*CH$_2$*), 2.40 (s, 3H, CH_3), 3.99 (t, ^3J = 6.5 Hz, 4H, ArO*CH$_2$*CH$_2$), 6.46 (d, ^3J = 15.8 Hz, 2H, Ar-CH=*CH*), 6.80 (d, ^3J = 8.3 Hz, 2H, Ar-H), 6.86 (s, 2H, Ar-H), 6.91 (d, ^3J = 8.9 Hz, 4H, Ar-H), 7.01 (d, ^3J = 7.9 Hz, 2H, Ar-H), 7.31 (m, 1H, Ar-H), 7.42 (d, ^3J = 8.5 Hz, 2H, Ar-H), 7.52 (d, ^3J = 8.7 Hz, 4H, Ar-H), 7.82 (d, ^3J = 16.0 Hz, 2H, Ar-*CH*=CH), 8.55 (s, 2H, CH=N).

N,N'-Bis[4-(4-n-dodecyloxycinnamoyloxy)-2-hydroxybenzylidene]-4-methyl-1,3-phenylenediamine (**OH 6c**)

Following GP2 from 4-formyl-3-hydroxyphenyl 4-*n*-dodecyloxycinnamate (**9**) and 4-methyl-1,3-phenylenediamine. Yield: 77%, yellow solid (DMF/Ethanol), $C_{63}H_{78}O_8N_2$, M = 991.27 g/mol.
^{1}H NMR (400 MHz, $CDCl_3$): δ 0.87 (t, ^3J = 6.8 Hz, 6H, CH_3), 1.25-1.52 (m, 36H, CH_2), 1.79 (m, 4H, ArOCH_2*CH_2*), 2.40 (s, 3H, CH_3), 3.99 (t, ^3J = 6.5 Hz, 4H, ArO*CH_2*CH_2), 6.46 (d, ^3J = 15.9 Hz, 2H, Ar-CH=*CH*), 6.80 (m, 2H, Ar-H), 6.86 (s, 2H, Ar-H), 6.91 (d, ^3J = 8.9 Hz, 4H, Ar-H), 7.03 (s, 1H, Ar-H), 7.12 (d, ^3J = 7.9 Hz, 1H, Ar-H), 7.30 (d, ^3J = 8.3 Hz, 1H, Ar-H), 7.43 (m, 2H, Ar-H), 7.52 (d, ^3J = 8.3 Hz, 4H, Ar-H), 7.82 (d, ^3J = 16.0 Hz, 2H, Ar-*CH*=CH), 8.66 (s, 2H, CH=N).

References

1 Kelly, S. M. *Helv. Chim. Acta* **1989**, *72*, 594-607.
2 Keith, Ch.; Dantlgraber, G.; Amaranatha Reddy, R.; Baumeister, U.; Tschierske, C. *Chem. Mater*. **2007**, *19*, 694-710.
3 Berdague, P.; Perez, F.; Coutieu, J.; Bayle, J.-P. *Bull. Soc. Chim. Fr*. **1994**, *131*, 335-343.
4 Yelamaggad, C. V.; Mathews, M.; Nagamani, S. A.; Rao, D. S. S.; Prasad, S. K.; Findeisen, S.; Weissflog, W. *J. Mater. Chem*. **2007**, *17*, 284-298.
5 Dave, J. S.; Patel, P. R. *Mol. Cryst. Liq. Cryst*. **1967**, *2*, 115-123.
6 Weissflog, W.; Naumann, G.; Kosata, B.; Schroeder, M. W.; Eremin, A.; Diele, S.; Vakhovskaya, Z.; Kresse, H.; Friedemann, R.; Rama Krishnan, A.; *J. Mater. Chem.* **2005**, *15*, 4328-4337.
7 Kosata, B.; Tamba, M. G.; Baumeister, U.; Pelz, K.; Diele, S.; Pelzl, G.; Galli, G.; Samaritani, S.; Agina, E.V.; Boiko, N. I.; Shibaev, V. P.; W. Weissflog *Chem. Mat*. **2006**, *18*, 691-701.
8 Dave, J. S.; Kurian, G.; Patel, N. R. *Indian J. Chem. A* **1980**, *19A*, 1161-1164.
9 Majumdar, K. C.; Chakravorty, S.; Pal, N. *Mol. Cryst. Liq. Cryst*. **2009**, *503*, 112-125.
10 Rao, N. V. S.; Deb, R.; Paul, M. K.; Francis, T. *Liq. Cryst*. **2009**, *36*, 977-987.
11 Guillevic, M.-A.; Light, M. E.; Coles, S. J.; Gelbrich, T.; Hursthouse, M. B.; Bruce, D. *J. Chem Soc., Dalton Trans*. **2000,** 1437-1445.
12 Enz, E.; Findeisen-Tandel, S.; Dabrowski, R.; Giesselmann, F.; Weissflog, W.; Baumeister, U.; Lagerwall, J. *J. Mater. Chem*. **2009**, *19*, 2950-2957.
13 Akutagawa, T.; Matsunaga, Y.; Yasuhara, K. *Liq. Cryst*. **1994**, *17*, 659-666.
14 Shreenivasa Murthy, H. N.; Sadashiva, B. K. *J. Mater. Chem*. **2003**, *13*, 2863-2871.
